# First-line treatment of anti-EGFR monoclonal antibody cetuximab β plus FOLFIRI versus FOLFIRI alone in Chinese patients with *RAS/BRAF* wild-type metastatic colorectal cancer: a randomized, phase 3 trial

**DOI:** 10.1038/s41392-025-02229-4

**Published:** 2025-05-07

**Authors:** Yuankai Shi, Yi Ba, Junye Wang, Jianping Xiong, Kangsheng Gu, Yigui Chen, Zhendong Zheng, Zishu Wang, Weijian Guo, Ying Cheng, Xianli Yin, Yunpeng Liu, Yuxian Bai, Enxiao Li, Qi Li, Liangjun Zhu, Wei Li, Da Jiang, Jingdong He, Jiansi Chen, Jianguo Sun, Sheng Hou

**Affiliations:** 1 National Cancer Center/National Clinical Research Center for Cancer/Cancer Hospital, Chinese Academy of Medical Sciences & Peking Union Medical College, Beijing Key Laboratory of Clinical Study on Anticancer Molecular Targeted Drugs, Beijing, PR China; 2 Peking Union Medical College Hospital, Chinese Academy of Medical Sciences & Peking Union Medical College, Beijing, PR China; 3 Tianjin Medical University Cancer Institute & Hospital, Tianjin, PR China; 4 Affiliated Hospital of Jining Medical University, Jining, PR China; 5 The First Affiliated Hospital of Nanchang University, Nanchang, PR China; 6 The First Affiliated Hospital of Anhui Medical University, Hefei, PR China; 7 Fujian Cancer Hospital, Fuzhou, PR China; 8 General Hospital of Northern Theater Command, Shenyang, PR China; 9 The First Affiliated Hospital of Bengbu Medical College, Bengbu, PR China; 10 Fudan University Shanghai Cancer Center, Shanghai, PR China; 11 Jilin Cancer Hospital, Changchun, PR China; 12 Hunan Cancer Hospital, Changsha, PR China; 13 The First Hospital of China Medical University, Shenyang, PR China; 14 Harbin Medical University Cancer Hospital, Harbin, PR China; 15 The First Affiliated Hospital of Xi’an Jiaotong University, Xi’an, PR China; 16 Shanghai General Hospital, Shanghai, PR China; 17 Jiangsu Cancer Hospital, Nanjing, PR China; 18 The First Hospital of Jilin University, Changchun, PR China; 19 The Fourth Hospital of Hebei Medical University, Shijiazhuang, PR China; 20 Huai’an First People’s Hospital, Huai’an, PR China; 21 Affiliated Tumor Hospital of Guangxi Medical University, Nanning, PR China; 22 The Second Affiliated Hospital of Army Medical University, Chongqing, PR China; 23 State Key Laboratory of Macromolecular Drugs and Large-scale Manufacturing, Taizhou Mabtech Pharmaceutical Co. Ltd, Taizhou, PR China

**Keywords:** Gastrointestinal cancer, Drug development, Tumour biomarkers, Tumour immunology, Molecular engineering

## Abstract

Cetuximab plus irinotecan, fluorouracil, and leucovorin (FOLFIRI) represents a first-line therapeutic standard for *RAS/BRAF* wild-type metastatic colorectal cancer (mCRC) patients. Despite this established approach, cetuximab β (CMAB009), as a modified antibody of cetuximab, prospectively selected for dual *RAS/BRAF* wild-type patients, has not yet been validated in the Chinese mCRC patients through phase 3 trial. In this study (ClinicalTrials.gov identifier: NCT03206151), patients with *RAS/BRAF* wild-type mCRC who were not suitable for radical resection were randomly assigned in a 1:1 ratio to receive cetuximab β plus FOLFIRI or FOLFIRI alone. The primary endpoint was blinded independent review committee-assessed progression-free survival (PFS). The secondary endpoints included overall survival (OS), objective response rate (ORR), disease control rate (DCR), surgery rate for metastasis and R0 resection rate, and safety. From January 4, 2018 to September 2, 2021, a total of 505 eligible patients were enrolled and received study treatment; the median follow-up duration was 8.7 months (95% confidence interval [CI], 7.77 to 9.29) and 5.9 months (95% CI, 5.63 to 6.65) in cetuximab β plus FOLFIRI group and FOLFIRI group, respectively. Compared to FOLFIRI alone, cetuximab β plus FOLFIRI demonstrated statistically significant improvements in median PFS (13.1 vs. 9.6 months, hazard ratio [HR], 0.639; 95% CI, 0.468 to 0.872; *P* = 0.004), median OS (28.3 vs. 23.1 months, HR, 0.729; 95% CI, 0.551 to 0.965; *P* = 0.024), and ORR (69.1% vs. 42.3%, odds ratio, 3.090; 95% CI, 2.280 to 4.189; *P* < 0.001). Cetuximab β plus FOLFIRI exhibited manageable toxicity without novel safety signals. This study demonstrated that cetuximab β plus FOLFIRI provided significant clinical benefits as a first-line treatment for patients with *RAS*/*BRAF* wild-type mCRC. Compared to FOLFIRI alone, cetuximab β plus FOLFIRI therapy led to prolonged median PFS and OS while maintaining a manageable safety profile, offering a new treatment option for this patient population.

## Introduction

Colorectal cancer (CRC) ranked as the second most prevalent malignant tumor in China, with 517,100 newly diagnosed cases in 2022. CRC was responsible for 9.3% of all cancer-related deaths, totaling 240,000 deaths, of which 142,600 were males and 97,400 were females.^1^ The therapeutic paradigm for metastatic colorectal cancer (mCRC) has evolved substantially since 2008, transitioning toward molecularly guided precision oncology. These biomarkers include mutations in the *KRAS*, *NRAS*, and *BRAF* genes,^2,3^ as well as microsatellite instability (MSI) and mismatch repair deficiency (dMMR) status.^4^ The application of these biomarkers in clinical practice has significantly improved treatment efficacy and patient survival.

Cetuximab plus irinotecan, fluorouracil, and leucovorin (FOLFIRI) remains a cornerstone regimen for wild-type *RAS/BRAF* mCRC patients. The combination of cetuximab and FOLFIRI can produce a synergistic effect, enhancing the cytotoxic impact on tumor cells. This synergistic effect may be achieved through multiple mechanisms, including alteration of the tumor microenvironment, enhancement of cell cycle arrest, induction of apoptosis, and inhibition of tumor angiogenesis. For instance, FOLFIRI can induce DNA damage in tumor cells, while cetuximab can further inhibit the repair capacity of tumor cells, thereby enhancing the efficacy of chemotherapy.^5,6^ The CRYSTAL study validated the efficacy and safety of cetuximab plus FOLFIRI as a first-line regimen for mCRC in Caucasian populations.^7^ Through two retrospective analyses, the study explored the relationships between the mutation status of *KRAS* or *BRAF* genes in tumor tissues and the clinical efficacy of cetuximab,^8^ as well as the impact of *RAS* mutations on treatment outcomes.^9^ The TAILOR study investigated the efficacy and safety of cetuximab plus leucovorin calcium, fluorouracil, and oxaliplatin (FOLFOX) in Chinese patients with *RAS* wild-type mCRC.^10^

Cetuximab β (CMAB009) is a recombinant human/mouse chimeric epidermal growth factor receptor (EGFR) monoclonal antibody (mAb). Compared to cetuximab, its amino acid sequences are identical, with minor differences in glycosylation and post-translational modifications. Unlike the SP2/0 cell-derived cetuximab, cetuximab β is produced via Chinese Hamster Ovary cell expression systems. This selection ensures that the engineered cell line for cetuximab β is devoid of the α-1,3-galactosyltransferase, which precludes the synthesis of the galactose-α-1,3-galactose (Gal (α 1–3) Gal), a structure that is highly prone to induce hypersensitivity reactions.^11,12^ Additionally, the constant region of the heavy chain in cetuximab β is characterized by a lower level of high-mannose content, which contributes to a further reduction in its immunogenicity.^13^

Cetuximab β has completed a phase 1 trial and pharmacokinetic study in advanced solid tumor patients,^14,15^ as well as an investigation into the efficacy and safety of its combination with irinotecan for second-line treatment of *KRAS* wild-type mCRC.^16^ In individuals with wild-type K*RAS* mCRC who experienced treatment failure following initial FOLFOX, cetuximab β plus irinotecan showed a markedly improved objective response rate (ORR) and extended progression-free survival (PFS) compared to irinotecan alone, with a manageable safety profile. To address the first-line applications evidence, we undertook this prospective phase 3 trial to compare the efficacy and safety of cetuximab β plus FOLFIRI with FOLFIRI alone for Chinese patients with *RAS/BRAF* wild-type mCRC. As a modified mAb of cetuximab, cetuximab β is expected to further validate the clinical benefits of EGFR mAbs in combination with chemotherapy in Chinese patients with *RAS/BRAF* wild-type mCRC through this phase 3 trial.

## Results

### Patient characteristics

**Fig. 1:**
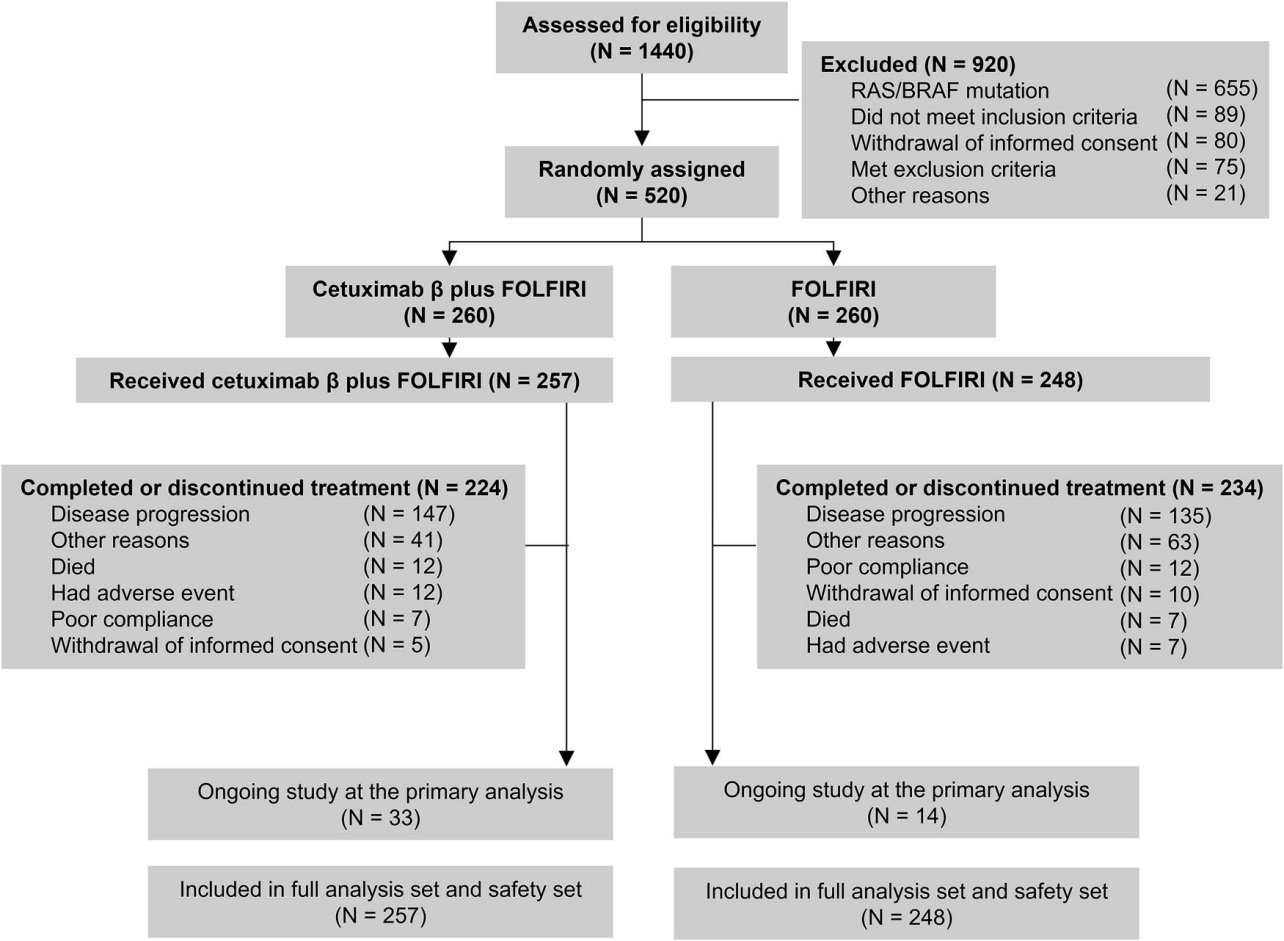
Patient disposition. FOLFIRI, irinotecan, fluorouracil, and leucovorin

From January 4, 2018, to September 2, 2021, a total of 1440 patients across 73 hospitals in China underwent eligibility screening. Predefined exclusion criteria eliminated 920 patients from this study: 655 patients had *RAS/BRAF* mutations, 89 failed to satisfy the inclusion criteria, 80 withdrew their informed consents, 75 met the exclusion criteria, and 21 were excluded for other reasons. The *RAS/BRAF* mutation rate was 45.5% (655/1440). Of 520 patients initially randomized, the full analysis set (FAS) was composed of 505 patients who were randomly assigned to the study and had received at least one dose of the study treatment. At the data cut-off date on May 6, 2022, ongoing treatment persisted in 33 patients in cetuximab β plus FOLFIRI group versus 14 patients in FOLFIRI group (Fig. 1).

The FAS population exhibited comparable age distributions between groups: median 57 years (range, 23–74) for cetuximab β plus FOLFIRI group versus median 58 years (range, 23–75) for FOLFIRI group. Tumor location analysis revealed right-sided primary tumors in 12.9% (65/505) patients and left-sided primary tumors in 85.7% (433/505) patients. Specifically, right-sided primary tumors were observed in 13.2% (34/257) of patients receiving cetuximab β plus FOLFIRI and 12.5% (31/248) of those in FOLFIRI group. Left-sided primary tumors were reported in 85.6% (220/257) of patients treated with cetuximab β plus FOLFIRI and 85.9% (213/248) of those treated with FOLFIRI. The baseline characteristics were well balanced between treatment groups (Table 1). The median follow-up time was 8.7 months (95% confidence interval [CI], 7.77 to 9.29) and 5.9 months (95% CI, 5.63 to 6.65) in cetuximab β plus FOLFIRI group and FOLFIRI group, respectively.

**Table 1:** Baseline characteristics in the FAS

	No. of Patients (%)	
Characteristic	Cetuximab β plus FOLFIRI (*n* = 257)	FOLFIRI (*n* = 248)
Sex		
Male	179 (69.6)	170 (68.5)
Female	78 (30.4)	78 (31.5)
Age, years		
Median	57	58
Range	23-74	23-75
ECOG PS score		
0	85 (33.1)	81 (32.7)
1	172 (66.9)	167 (67.3)
No. of metastatic disease sites		
1	85 (33.1)	71 (28.6)
2	95 (37.0)	83 (33.5)
3	46 (17.9)	54 (21.8)
＞ 3	31 (12.1)	40 (16.1)
Metastatic sites		
Liver	179 (69.6)	189 (76.2)
Intraperitoneal lymph nodes	94 (36.6)	102 (41.1)
Extraperitoneal lymph nodes	68 (26.5)	71 (28.6)
Lung	83 (32.3)	95 (38.3)
Peritoneum	25 (9.7)	23 (9.3)
Other	74 (28.8)	66 (26.6)
Primary tumor location		
Right-sided^a^	34 (13.2)	31 (12.5)
Left-sided^b^	220 (85.6)	213 (85.9)
Other	3 (1.2)	4 (1.6)
Previous therapy		
Neoadjuvant chemotherapy	5 (1.9)	7 (2.8)
Surgery	123 (47.9)	120 (48.4)
Radiotherapy	10 (3.9)	16 (6.5)
Adjuvant chemotherapy	62 (24.1)	39 (15.7)
Other	11 (4.3)	13 (5.2)
Primary tumor resection		
Yes	114 (44.4)	108 (43.5)
No	143 (55.6)	140 (56.5)

*ECOG* Eastern Cooperative Oncology Group, *FAS* full analysis set, *PS* performance status

^a^Right-sided: cecum, ascending colon, hepatic flexure, and transverse colon

^b^Left-sided: splenic flexure, descending colon, sigmoid colon, and rectum

### Study drug exposure

At the data cut-off date of primary analysis, median exposure periods in cetuximab β plus FOLFIRI group were 7.3 months (interquartile range, 3.7 to 11.0) for cetuximab β treatment, 5.8 months (interquartile range, 3.4 to 9.2) for irinotecan treatment, and 6.0 months (interquartile range, 3.4 to 9.3) for 5-fluorouracil treatment. FOLFIRI group had a median duration of 5.1 months (interquartile range, 2.3 to 7.3) for irinotecan treatment and 5.2 months (interquartile range, 2.4 to 7.3) for 5-fluorouracil treatment. The mean relative dose-intensity of irinotecan (0.927 vs. 0.949) and 5-fluorouracil (0.923 vs. 0.932) confirmed balanced treatment exposure between groups (Supplemental Table 1). Notably, 57.2% (147/257) of patients continued cetuximab β treatment for more than 6 months, while 19.8% (51/257) remained on cetuximab β therapy for a period surpassing 12 months.

### Efficacy

**Fig. 2:**
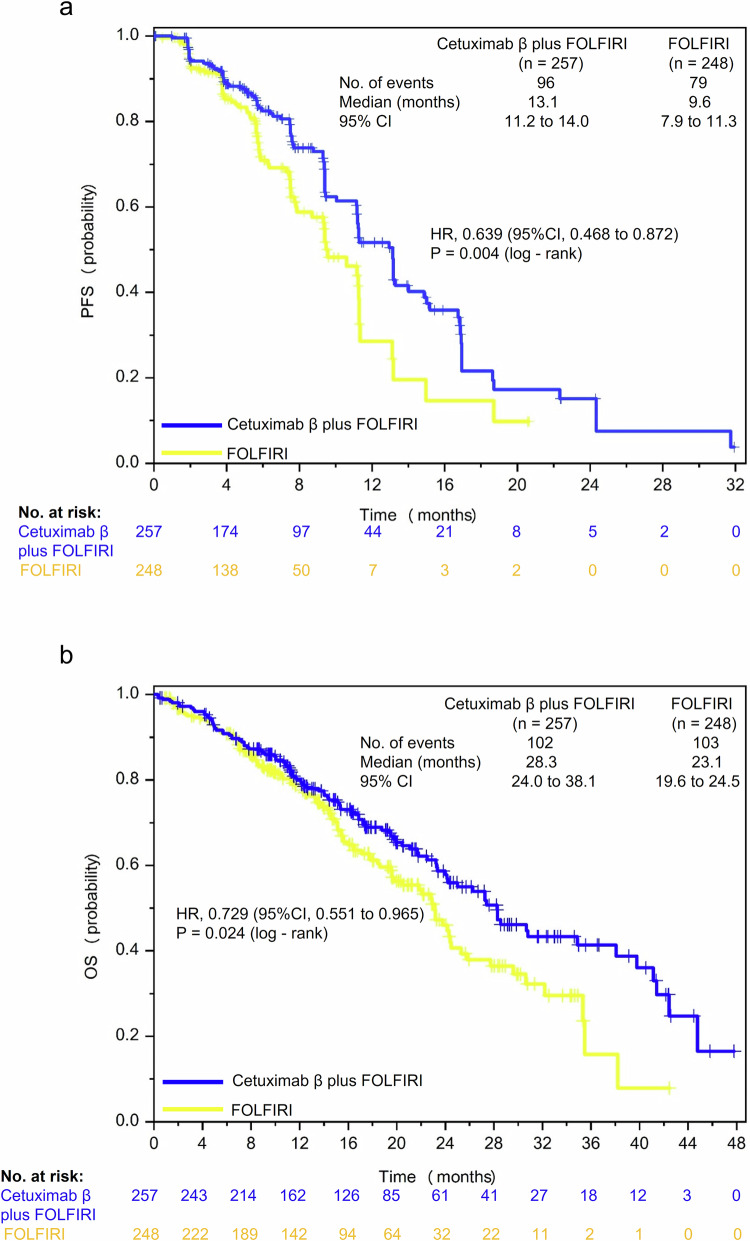
PFS and OS of patients in the two treatment groups. **a** Kaplan–Meier estimates of BIRC-assessed PFS in the FAS. **b** Kaplan–Meier estimates of OS in the FAS. Crosses denote censored patients. BIRC blinded independent review committee, CI confidence interval, FAS full analysis set, FOLFIRI irinotecan, fluorouracil, and leucovorin, HR hazard ratio, OS overall survival, PFS progression-free survival

The blinded independent review committee (BIRC)-assessed median PFS of cetuximab β plus FOLFIRI group was significantly prolonged compared to FOLFIRI group (13.1 vs. 9.6 months, hazard ratio [HR], 0.639; 95% CI, 0.468 to 0.872; *P* = 0.004; Table 2; Fig. 2). A similar trend was observed in investigator-assessed PFS, where cetuximab β plus FOLFIRI group also demonstrated superior outcomes compared to FOLFIRI group (11.1 vs. 7.5 months, HR, 0.559; 95% CI, 0.439 to 0.710; *P* < 0.001; Supplemental Table 2; Supplemental Fig. 1a). In two sensitivity analyses, the statistical outcomes were consistent with those of the primary analysis. The median BIRC-assessed PFS in the FAS, when progression of disease following the loss to follow-up of tumor assessment was considered as an event, of cetuximab β plus FOLFIRI group and FOLFIRI group was 11.5 months (95% CI, 11.2 to 13.2) and 9.4 months (95% CI, 7.9 to 10.3), respectively (HR, 0.590; 95% CI, 0.444 to 0.785; *P* < 0.001; Supplemental Fig. 1b). The median BIRC-assessed PFS in the FAS, when progression of disease or death following the loss to follow-up of tumor assessment was considered as an event, of cetuximab β plus FOLFIRI group and FOLFIRI group was 11.5 months (95% CI, 11.2 to 13.2) and 9.4 months (95% CI, 8.0 to 11.0), respectively (HR, 0.734; 95% CI, 0.584 to 0.923; *P* = 0.008; Supplemental Fig. 1c). Regarding overall survival (OS), cetuximab β plus FOLFIRI group achieved a median OS of 28.3 months (95% CI, 24.0 to 38.1), exceeding 23.1 months (95% CI, 19.6 to 24.5) observed in FOLFIRI group (HR, 0.729; 95% CI, 0.551 to 0.965; *P* = 0.024; Table 2; Fig. 2).

**Table 2:** Efficacy in the FAS

Efficacy	Cetuximab β plus FOLFIRI (*n* = 257)	FOLFIRI (*n* = 248)
PFS		
No. of events (PD or death), *n* (%)^a^	96 (37.4)	79 (31.9)
Median (95% CI), months^b^	13.1 (11.2, 14.0)	9.6 (7.9, 11.3)
HR (95% CI)	0.639 (0.468, 0.872)	
*P* value^c^	0.004	
OS		
No. of events (death), *n* (%)	102 (39.7)	103 (41.5)
Median (95% CI), months^b^	28.3 (24.0, 38.1)	23.1 (19.6, 24.5)
HR (95% CI)	0.729 (0.551, 0.965)	
*P* value^c^	0.024	
Best overall response, *n* (%)		
Complete response	0 (0.0)	3 (1.3)
Partial response	161 (69.1)	93 (41.0)
Stable disease	44 (18.9)	108 (47.6)
Progressive disease	20 (8.6)	19 (8.4)
Not evaluable	8 (3.4)	4 (1.8)
ORR, *n* (%)	161 (69.1)	96 (42.3)
95% CI	63.2, 75.0	35.9, 48.7
OR (95% CI)	3.090 (2.280, 4.189)	
* P* value^c^	<0.001	
DCR, *n* (%)	205 (88.0)	204 (89.9)
95% CI	83.8, 92.2	85.9, 93.8
OR (95% CI)	0.814 (0.477, 1.389)	
*P* value^c^	0.520	

Except for OS, all other efficacy endpoints were based on BIRC-assessed data

*BIRC* blinded independent review committee, *CI* confidence interval, *DCR* disease control rate, *FAS* full analysis set, *HR* hazard ratio, *OR* odds ratio, *ORR* objective response rate, *OS* overall survival, *PD* progressive disease, *PFS* progression-free survival

^a^Death occurring within 90 days following the last tumor response evaluation or the date of randomization

^b^Kaplan–Meier estimates

^c^*P* values were calculated with the use of the log-rank test or, in the case of ORR, the Cochran–Mantel–Haenszel test

According to BIRC assessment, the ORR of cetuximab β plus FOLFIRI group and FOLFIRI group was 69.1% (95% CI, 63.2% to 75.0%) and 42.3% (95% CI, 35.9% to 48.7%), respectively (odds ratio [OR], 3.090; 95% CI, 2.280 to 4.189; *P* < 0.001; Table 2). The investigator-assessed ORR of cetuximab β plus FOLFIRI group and FOLFIRI group was 63.1% (95% CI, 56.7% to 69.4%) and 37.2% (95% CI, 30.9% to 43.5%), respectively (OR, 2.890; 95% CI, 1.953 to 4.276; *P* < 0.001; Supplemental Table 2). The disease control rate (DCR) was similar between the two groups, with 88.0% (95% CI, 83.8% to 92.2%) in cetuximab β plus FOLFIRI and 89.9% (95% CI, 85.9% to 93.8%) in FOLFIRI group (OR, 0.814; 95% CI, 0.477 to 1.389; *P* = 0.520; Table 2). The surgery rate for metastasis with curative intent was 7.4% and 1.6% in cetuximab β plus FOLFIRI group and FOLFIRI group, respectively (*P* = 0.002). The R0 resection rate was 4.7% and 1.2% in cetuximab β plus FOLFIRI group and FOLFIRI group, respectively (*P* = 0.640).

**Fig. 3:**
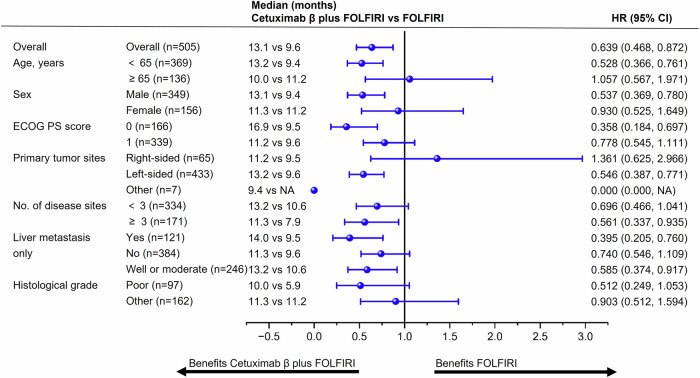
Subgroup analysis for BIRC-assessed PFS in the FAS. BIRC blinded independent review committee, CI confidence interval, ECOG Eastern Cooperative Oncology Group, FAS full analysis set, FOLFIRI irinotecan, fluorouracil, and leucovorin, HR hazard ratio, PFS progression-free survival, PS performance status

A post hoc exploratory subgroup analysis was performed for BIRC-assessed PFS based on patient demographics and disease characteristics (Fig. 3). In patients with left-sided primary tumors, cetuximab β plus FOLFIRI regimen demonstrated a notable PFS advantage over FOLFIRI, with median PFS of 13.2 months versus 9.6 months (HR, 0.546; 95% CI, 0.387 to 0.771). However, for those with right-sided primary tumors, no statistically significant PFS difference was observed between the two groups. A similar trend was noted in OS outcomes (Supplemental Fig. 2).

### Safety

Among 505 patients included in the safety set, who were randomly assigned to the study and received at least one dose of study treatment and one safety evaluation, the incidence of at least one ≥ grade 3 treatment-emergent adverse event (TEAE) was 83.3% (214/257) in cetuximab β plus FOLFIRI group, compared to 66.9% (166/248) in FOLFIRI group. The proportion of patients experiencing ≥ grade 3 treatment-related adverse events (TRAEs) was 78.6% in cetuximab β plus FOLFIRI group and 57.3% in FOLFIRI group.

The most frequently observed ≥ grade 3 TRAE was absolute neutrophil count decreased, occurring in 56.8% (146/257) of patients receiving cetuximab β plus FOLFIRI and 34.7% (86/248) of those receiving FOLFIRI. The skin reaction rates in cetuximab β plus FOLFIRI group and FOLFIRI group were: dermatitis, 45.5% vs. 2.4%; dermatitis acneiform, 21.0% vs. 0%; palmoplantar pustulosis, 11.7% vs. 1.6%. No grade 4 skin reactions were reported. Additionally, notable inter-group differences (≥10%) were found in aspects such as alanine aminotransferase increased, weight decreased, and hypomagnesemia, all of which were more prevalent in cetuximab β plus FOLFIRI group (Table 3).

**Table 3:** Incidence > 10% TRAEs in the SS

TRAEs	No. of Patients (%)			
	Cetuximab β plus FOLFIRI (*n* = 257)		FOLFIRI (*n* = 248)	
	Any Grade	≥Grade 3	Any Grade	≥Grade 3
ANC decreased	218 (84.8)	146 (56.8)	154 (62.1)	86 (34.7)
WBC count decreased	212 (82.5)	84 (32.7)	154 (62.1)	41 (16.5)
ALT increased	91 (35.4)	3 (1.2)	48 (19.4)	0
AST increased	76 (29.6)	4 (1.6)	53 (21.4)	2 (0.8)
Weight decreased	62 (24.1)	1 (0.4)	32 (12.9)	0
PLT count decreased	48 (18.7)	6 (2.3)	33 (13.3)	3 (1.2)
Blood bilirubin increased	49 (19.1)	1 (0.4)	31 (12.5)	2 (0.8)
Lymphocyte count decreased	40 (15.6)	10 (3.9)	27 (10.9)	5 (2.0)
Blood ALP increased	28 (10.9)	1 (0.4)	22 (8.9)	2 (0.8)
Nausea	128 (49.8)	4 (1.6)	127 (51.2)	1 (0.4)
Diarrhoea	125 (48.6)	26 (10.1)	107 (43.1)	31 (12.5)
Vomiting	77 (30.0)	4 (1.6)	97 (39.1)	10 (4.0)
Constipation	40 (15.6)	0	37 (14.9)	1 (0.4)
Abdominal pain	32 (12.5)	2 (0.8)	37 (14.9)	0
Stomatitis	44 (17.1)	8 (3.1)	13 (5.2)	3 (1.2)
Decreased appetite	104 (40.5)	5 (1.9)	97 (39.1)	1 (0.4)
Hypoalbuminemia	47 (18.3)	1 (0.4)	37 (14.9)	0
Hypokalemia	50 (19.5)	11 (4.3)	18 (7.3)	6 (2.4)
Hypomagnesemia	46 (17.9)	5 (1.9)	5 (2.0)	0
Hyponatremia	30 (11.7)	5 (1.9)	15 (6.0)	3 (1.2)
Dermatitis	117 (45.5)	16 (6.2)	6 (2.4)	1 (0.4)
Alopecia	51 (19.8)	0	43 (17.3)	0
Dermatitis acneiform	54 (21.0)	3 (1.2)	0	0
Palmoplantar pustulosis	30 (11.7)	1 (0.4)	4 (1.6)	0
Anemia	118 (45.9)	13 (5.1)	123 (49.6)	18 (7.3)
Fatigue	86 (33.5)	5 (1.9)	63 (25.4)	4 (1.6)
Pyrexia	26 (10.1)	1 (0.4)	12 (4.8)	1 (0.4)
Cholinergic syndrome	11 (4.3)	0	27 (10.9)	1 (0.4)
Paronychia	40 (15.6)	8 (3.1)	0	0
Hypersensitivity	2 (0.8)	0	1 (0.4)	0
Composite categories				
Cetuximab β-induced infusion-related reaction	33 (12.8)	1 (0.4)	NA	NA
Chemotherapy-induced infusion-related reaction	24 (9.3)	1 (0.4)	10 (4.0)	1 (0.4)

*ALP* alkaline phosphatase, *ALT* alanine aminotransferase, *ANC* absolute neutrophil count, *AST* aspartate aminotransferase, *PLT* platelet, *SS* safety set, *TRAEs* treatment-related adverse events, *WBC* white blood cell

There were 12.8% (33/257) of patients who experienced cetuximab β-induced infusion-related reactions, which were defined as allergic reactions occurring at any time or symptoms such as sinus tachycardia, hyperhidrosis, fever, respiratory distress, blood pressure changes, and myocardial ischemia occurring on the first day of administration. 9.3% (24/257) and 4.0% (10/248) of patients in cetuximab β plus FOLFIRI group and FOLFIRI group had chemotherapy-induced infusion-related reactions, respectively. Hypersensitivity reactions are also a manifestation of infusion-related reactions, with 0.8% (2/257) and 0.4% (1/248) in cetuximab β plus FOLFIRI group and FOLFIRI group, respectively, and no ≥grade 3 hypersensitivity reactions were reported (Table 3). Based on this study of 505 patients, the risk ratio for grade 1–4 hypersensitivity reactions with cetuximab β was 1.93 (95% CI, 0.18 to 21.28).

### Immunogenicity

A total of 241 patients underwent anti-drug antibody (ADA) testing, with a post-treatment ADA positivity rate of 5.4% (13/241). Among all 36 ADA-positive samples, only 2 were positive for neutralizing antibody (NAb), both occurring at baseline, resulting in a post-administration NAb positivity rate of 0%. Among the 13 ADA-positive patients, 8 developed new ADA positivity or experienced a treatment-induced increase, most occurring at week 4. The adverse events (AEs) that occurred in these eight patients were sporadic, with no suspected AEs attributed to immune abnormalities, and no drug allergies. This suggests that the formation of ADA has no impact on safety.

## Discussion

This study achieved its primary endpoint, with a statistically significant difference in BIRC-assessed PFS between the two treatment groups. The investigator-assessed PFS and other sensitivity analyses confirmed the robustness of this positive outcome. In addition, the OS and ORR were also superior in cetuximab β plus FOLFIRI group compared to FOLFIRI group. Several randomized controlled trials (RCTs) have confirmed that the therapeutic efficacy of FOLFOX and FOLFIRI is comparable, and they can be interchanged in clinical practice.^17,18^ Although this study only utilized the FOLFIRI regimen, it is reasonable to infer that cetuximab β plus FOLFOX would likely exhibit similar anti-tumor effects and manageable AEs. Cetuximab β for mCRC is not limited to first-line treatment, our previous study has demonstrated clinical benefits from cetuximab β combined with irinotecan in second-line therapy and its use as monotherapy in third-line treatment for *KRAS* wild-type mCRC.^16^ Additional RCTs have indicated that in patients with initially unresectable liver metastases of *RAS* wild-type mCRC, cetuximab plus chemotherapy (FOLFOX or FOLFIRI) yields a higher resection rate compared to chemotherapy alone or to bevacizumab plus chemotherapy regimens.^19,20^ This enhanced resection rate may be associated with a greater early tumor shrinkage and depth of response. Consequently, for patients with initially unresectable liver metastases from left-sided *RAS* wild-type mCRC, EGFR mAb plus chemotherapy should be considered as a preferred option with greater potential benefit.^21,22^

The therapeutic horizon for mCRC is evolving towards biomarker-driven strategies, with the combination of EGFR mAbs and specific mutational inhibitors offering new treatment options. In addition to agents for *BRAF* V600E mutation,^23–25^ recent findings have altered the perception that *KRAS* G12C mutation is undruggable. In an expanded cohort of a multicenter, single-arm study, 94 patients with locally advanced or metastatic CRC harboring *KRAS* G12C mutation, who had previously received chemotherapy with fluoropyrimidines, oxaliplatin, or irinotecan, achieved an ORR of 34% treated with adagrasib plus cetuximab.^26,27^ Another randomized, open-label, phase 3 trial among patients with chemotherapy-refractory *KRAS* G12C mutated mCRC demonstrated an ORR of 26.4% treated with sotorasib plus panitumumab, which is substantially higher than that of the investigator-selected control group with 0%.^28^ Given that cetuximab β shares an identical amino acid sequence with cetuximab, it will present a promising future in the therapeutic landscape of mCRC.

Compared with the phase 1 study and the phase 3 study of cetuximab β for second-line treatment of mCRC,^14–16^, no new or unexpected safety profile occurred. Cetuximab β plus FOLFIRI was associated with an increased incidence and severity of hematological toxicities; the rate of hepatic dysfunction also increased, though the severity did not notably escalate. However, these AEs were generally manageable. Regarding the analysis of infusion-related reactions in this study, we first compiled all AEs defined as infusion-related reactions from 4 cetuximab studies (CRYSTAL, Study CA225-025, BONNER, and EXTREME). These AEs were then compared with those observed in this study one by one, and a comprehensive statistical analysis was performed. This led to the determination of an infusion-related reaction incidence rate of 12.8%, which is relatively conservative. The CRYSTAL study, conducted between 2004 and 2005, reported an infusion-related reaction incidence of 2.5%.^7–9^ Differences in clinical practice, increased understanding of the target, and variations in the frequency of examinations may have contributed to the discrepancy in infusion-related reaction incidence rates between the CRYSTAL study and this study. Of course, comparing infusion-related reactions between two drugs within the same randomized controlled trial would be more scientific and persuasive.

Hypersensitivity reactions are a known safety feature associated with cetuximab.^11^ Due to the absence of Gal (α 1–3) Gal structure, the incidence of hypersensitivity reactions for cetuximab β is significantly decreased, with any grade being 0.8% and ≥grade 3 being 0. A meta-analysis of hypersensitivity reactions of cetuximab included 6047 patients, with a risk ratio of grade 1–4 hypersensitivity reaction of 5.47 (95% CI, 3.80 to 7.87).^29^ In this study, the risk ratio for grade 1–4 hypersensitivity reactions with cetuximab β was 1.93 (95% CI, 0.18 to 21.28), indicating that the glycosylation modification indeed contributes to improved safety profile.

This study has some limitations. At the time of design of this study, cetuximab had not yet been approved by China National Medical Products Administration as first-line treatment for mCRC until November 13, 2019. Therefore, FOLFIRI was selected as control group of this study. Cetuximab β has an identical amino acid sequence to cetuximab. The primary consideration was to compare it with cetuximab rather than bevacizumab. Therefore, bevacizumab combined with chemotherapy was not selected as control group. Further study is needed to compare the efficacy and safety between cetuximab β and other EGFR mAbs, as well as bevacizumab. Pembrolizumab in the KEYNOTE-177 study^30,31^ and nivolumab plus ipilimumab regimen in CheckMate-8HW study^32^ also disclosed their efficacy and safety data after the conduct of this study and were subsequently approved for first-line treatment of mCRC. Therefore, patients were not mandatorily required to undergo detection of mismatch repair (MMR) status during screening, and patients with dMMR mCRC were also included in this study. Since dMMR occurs in only ~4–5% of all mCRC patients,^31^ it can be inferred that this small subset of patients would not have a reversing impact on this study’s efficacy or safety outcomes.

In conclusion, this study demonstrated that cetuximab β plus FOLFIRI provided significant clinical benefits as a first-line treatment for patients with *RAS/BRAF* wild-type mCRC. Compared to FOLFIRI alone, cetuximab β plus FOLFIRI therapy led to prolonged median PFS and OS while maintaining a manageable safety profile, offering a new treatment option for the patient population.

## Materials and methods

### Patients

This study (ClinicalTrials.gov identifier: NCT03206151) was an open-label, multicenter, randomized, parallel-group, phase 3 trial for Chinese patients diagnosed with histologically confirmed *RAS*/*BRAF* wild-type mCRC. Eligible patients included those who were not suitable for radical resection and had not have received systemic chemotherapy previously or those who finished chemotherapy ≥12 months before the detection of disease metastasis. There was at least one measurable lesion as defined by the Response Evaluation Criteria in Solid Tumors (RECIST) version 1.1. Additionally, all patients needed to have an Eastern Cooperative Oncology Group (ECOG) performance status (PS) score of 0 or 1 and demonstrated adequate hematologic, hepatic, and renal function. Key exclusion criteria encompassed the presence of *RAS*/*BRAF* mutation, symptomatic brain or leptomeningeal metastases, as well as any prior exposure to mAb or signal transduction inhibitors targeting EGFR.

The study adhered to the ethical principles outlined in the Declaration of Helsinki and complied with all relevant regulatory requirements. The protocol and all amendments received approval from the ethics committees of all participating hospitals, including the two leading hospitals, specifically the National Cancer Center/Cancer Hospital, Chinese Academy of Medical Sciences & Peking Union Medical College (Approval No. 17-031/1286), and Tianjin Medical University Cancer Institute & Hospital (Approval No. E2017105). Ethical approval details are available in the Supplementary Material. Prior to enrollment, all patients provided written informed consent.

### *RAS*/*BRAF* mutation testing

Tumor specimens that were formaldehyde-fixed and embedded in paraffin were forwarded to a designated central facility (Teddy Clinical Research Laboratory (Shanghai) Limited) for *RAS* and *BRAF* mutation testing. Samples were provided in the form of paraffin blocks or as 3–5 µm thick sections affixed to glass slides. Only tissue samples with a tumor cell proportion of ≥10% were selected for DNA extraction. The *RAS*/*BRAF* wild-type was defined as the absence of specific oncogenic mutations in the *RAS* gene family (*KRAS*, *NRAS*) and the *BRAF* gene. In this study, the specific oncogenic mutations include mutations in codons 12, 13, and 61 of exons 2, 3, and 4 of the *RAS* genes, as well as the V600 mutation in exon 15 of the *BRAF* gene. Mutation screening for *KRAS*/*NRAS*/*BRAF* genes was conducted using the amplification refractory mutation system in combination with fluorescent polymerase chain reaction technology.

### Study design and treatment

This study utilized a dynamic randomization method based on the Interactive Web Response System to randomly assign 520 patients in a 1:1 ratio to the cetuximab β plus FOLFIRI group and the FOLFIRI group. The randomization considered two balancing factors of ECOG PS (0 vs. 1) and hospital, ensuring that the difference between the two groups did not exceed 4 overall, and did not exceed 2 within each ECOG PS and hospital. The specific algorithm was as follows: if the overall or any balancing factor level’s group difference exceeded the set threshold, the allocation probability was adjusted (0.9:0.1) for random assignment; if all balancing factor levels’ group differences were within the set range, patients were assigned with a higher probability to the group with fewer patients (0.9:0.1) or with equal probability (0.5:0.5) in case of a tie.

Each treatment cycle spanned 14 days, and therapy was continued until disease progression, death, unacceptable toxicity, patient withdrawal of consent, initiation of a new anti-cancer regimen, or other protocol-defined reasons. All enrolled patients received an intravenous (IV) infusion of irinotecan at 180 mg/m^2^, followed by leucovorin at 400 mg/m^2^ IV and a bolus IV dose of 5-fluorouracil at 400 mg/m^2^ on the first day of each cycle. This was followed by a continuous IV infusion of 5-fluorouracil at a dose of 2400 mg/m^2^ over a period of 46 to 48 h. In cetuximab β plus FOLFIRI group, cetuximab β was administered on both day 1 and day 8 of each cycle, with an initial loading dose of 400 mg/m^2^, followed by weekly maintenance dose of 250 mg/m^2^.

### Outcomes

The primary endpoint was PFS assessed by BIRC, defined as the time from randomization to the first radiologically confirmed disease progression or death as a result of any cause. Secondary efficacy endpoints included OS, ORR, DCR, the surgery rate for metastasis, the R0 resection rate, and safety. A blinded review of imaging and clinical data for the primary endpoint of PFS and the secondary endpoint of ORR was carried out by a BIRC. While the BIRC review of image data served as the main statistical analysis, investigator assessments were used for on-study decisions and sensitivity analysis. Both the BIRC and investigators evaluated tumor responses in accordance with RECIST version 1.1. Radiographic assessments were performed at baseline and subsequently at eight-week intervals until disease progression. Patients were followed up for survival outcomes every three months.

Safety assessments included the frequency and classification of AEs, as well as laboratory evaluations, vital signs, physical examinations, and electrocardiograms. The severity of AEs was categorized according to the National Cancer Institute Common Terminology Criteria for Adverse Events version 4.03.

### Statistical analysis

To achieve 80% power for detecting differences between the two treatment groups at a two-sided significance level of *α* = 0.05, the study aimed to observe 339 events for the primary endpoint. This calculation was based on an expected median PFS of 11.4 months in cetuximab β plus FOLFIRI group and 8.4 months in FOLFIRI group, referencing findings from the CRYSTAL trial. A total of 410 patients were required to detect this absolute difference in PFS. Accounting for an anticipated 20% dropout rate, the final enrollment target was set at 512 patients, with 256 allocated to each group.

For time-to-event endpoints such as PFS and OS, the Kaplan-Meier method was employed to estimate medians and corresponding 95% CIs for both groups. The stratified log-rank test was utilized to assess the differences in efficacy between the treatment groups. The stratified Cox proportional hazard regression model was used to estimate the HR, with the stratification factors of ECOG PS and the geographical region of clinical trial center in the model. The Cochran-Mantel-Haenszel test was applied to compare ORR and DCR between groups.

## Supplementary information

Supplementary Materials

Study Protocol

Statistical Analysis Plan

## Data Availability

Dataset of this study could be obtained from corresponding authors with a reasonable request.
