# Supplementary material for: First-line treatment of anti-EGFR monoclonal antibody cetuximab β plus FOLFIRI versus FOLFIRI alone in Chinese patients with RAS/BRAF wild-type metastatic colorectal cancer: a randomized, phase 3 trial
Source: Signal Transduct Target Ther. 2025 May 7;10:147. doi: 10.1038/s41392-025-02229-4 (PMC12056184; doi:10.1038/s41392-025-02229-4)
Supplement: Supplementary file 1 — Supplementary Materials [file 41392_2025_2229_MOESM1_ESM.docx]

Supplementary Materials for

First-line Treatment of Anti-EGFR Monoclonal Antibody Cetuximab β Plus FOLFIRI Versus FOLFIRI Alone in Chinese Patients with *RAS/BRAF* Wild-type Metastatic Colorectal Cancer: A Randomized, Phase 3 Trial

Yuankai Shi1, Yi Ba, Junye Wang, Jianping Xiong, Kangsheng Gu, Yigui Chen, Zhendong Zheng, Zishu Wang, Weijian Guo, Ying Cheng, Xianli Yin, Yunpeng Liu, Yuxian Bai, Enxiao Li, Qi Li, Liangjun Zhu, Wei Li, Da Jiang, Jingdong He, Jiansi Chen, Jianguo Sun and Sheng Hou

Correspondence to: [syuankai@cicams.ac.cn](mailto:syuankai@cicams.ac.cn); [bayi@pumch.cn](mailto:bayi@pumch.cn).

**This PDF file includes:**

Materials and Methods

Supplementary Text

Figure S1 to S2

Table S1 to S2

**Other Supplementary Materials for this manuscript include the following:**

Study Protocol

Statistical Analysis Plan

**Materials and Methods**

Ethics committees of all participating hospitals and approval numbers

- National Cancer Center/Cancer Hospital, Chinese Academy of Medical Sciences & Peking Union Medical College: 17-031/1286.

- Tianjin Medical University Cancer Institute & Hospital: E2017105.

- The First Affiliated Hospital of Anhui Medical University: PJ2017-12-02.

- The First Affiliated Hospital of Bengbu Medical University: [2017] 068.

- Peking University Third Hospital: (2017) 105-01.

- The First Affiliated Hospital to Army Medical University: YW201732.

- The Second Affiliated Hospital to Army Medical University: 2017-004-01.

- Army Medical Center of PLA: (2018) 08.

- Fujian Cancer Hospital: 2017-033-01.

- Fujian Medical University Union Hospital: 2017YW040-02.

- Fudan University Shanghai Cancer Center: 1707174-9.

- The First Affiliated Hospital with Nanjing Medical University: 2017-MD-172.

- Jiangsu Cancer Hospital: 2017-018.

- Chinese PLA General Hospital: C2017-054-01.

- Liaoning Cancer Hospital & Institute: 20171115.

- The 900 Hospital of the Joint Logistic Support Force of the PLA: IEC-2017-137.

- The Affiliated Hospital of Qingdao University: QYFYEC 2018-051-01.

- Shanxi Cancer Hospital: (2017) 11.

- Shanghai Sixth People's Hospital: 2018-031-(1).

- Shanghai General Hospital: [2017] 28.

- Xiangya Hospital of Central South University: 201712148.

- The Second Xiangya Hospital of Central South University: (2018) 001.

- The Sixth Affiliated Hospital, Sun Yat-sen University: 2018ZSLYEC-045.

- Affiliated Cancer Hospital and Institute of Guangzhou Medical University: [2017] 20.

- The First Affiliated Hospital of Nanchang University: [2017] 063.

- The Fourth Hospital of Hebei Medical University: 2017102.

- Harbin Medical University Cancer Hospital: 2018-08.

- Hunan Cancer Hospital: 2017 [77].

- Tongji Hospital Tongji Medical College of HUST: [2017] 153.

- Union Hospital Tongji Medical College HUST: [2017] 154.

- The First Hospital of Jilin University: 18Y030-001.

- The Second Hospital of Jilin University: (2017) 017-1.

- Jilin Cancer Hospital: 201709-030-02.

- General Hospital of Northern Theater Command: (2017) 60.

- The First Affiliated Hospital of Xi'an Jiaotong University: 2018 (5).

- The First Affiliated Hospital of Air Force Medical University: YS20181026-1.

- The Second Affiliated Hospital Zhejiang University School of Medicine: (2017) 257.

- Zhejiang Cancer Hospital: IRB- [2017] 167.

- The First Hospital of China Medical University: 2017YL059.

- Baoji Central Hospital: 2018-7.

- General Hospital of Ningxia Medical University: 2019-YW-005.

- Guizhou Provincial People’s Hospital: (2019) 09.

- Affiliated Hospital of Jining Medical University: (2019) JYFY-2.

- Inner Mongolia Medical University Cancer Hospital: YW201906.

- Qinghai Provincial People’s Hospital: PJ 2018-007-01.

- Central Hospital Affiliated to Shangdong First Medical University: 2019-126-02.

- Hubei Cancer Hospital: [2019] 64.

- Taizhou Hospital of Zhejiang Province: 201933-01.

- Affiliated Hospital of Chengde Medical University: LL2019-009.

- Guangxi Medical University Cancer Hospital: KS2019 (111).

- The First People’s Hospital of Yunnan Province: KHLL2019-020.

- Henan Cancer Hospital: 2019102811.

- The First Affiliated Hospital of Zhengzhou University: L19YШ151-001.

- Chongqing University Cancer Hospital: CZLS2020077-B.

- Zhongnan Hospital of Wuhan University: 2019037.

- Pingxiang People’s Hospital: 2019D043-E02.

- Sichuan Provincial People’s Hospital: 2020-28-1.

- Huai'an First People's Hospital: YL-P-2020-003-01.

- Xiangya Hospital Zhuzhou Central South University: ZZCHGCPEC2020016-02.

- Affiliated Hospital of Zunyi Medical University: 2020-030.

- Affiliated Hospital of Qinghai University: 2020LLPJ-027.

- Affiliated Hospital of Guangdong Medical University: PJ2020-073.

- The First Hospital of Jiaxing: 2020-019.

- Gansu Provincial Hospital: 2020 (34).

- Baoding No.2 Central Hospital: 2021 (3).

- Weifang People’s Hospital: 2020-039-02.

- Yuncheng Central Hospital: 2020-YW-042.

- Beijing Yuhe Integrated Traditional Chinese and Western Medicine Rehabilitation Hospital: 2020-003.

- Hebei Petro China Central Hospital (Oncology): IRB2020-046-01.

- Hebei Petro China Central Hospital (General Surgery): IRB2020-046-05.

- The Second People’s Hospital of Neijiang: 2020-022-001.

- Zibo Bashan Wanjie Hospital: 2020-01-B003.

- The Second Hospital of Anhui Medical University: YW2020-147 (F1).

**Supplementary Text**

*RAS/BRAF* gene

The *RAS* (Rat Sarcoma) gene is one of the most critical oncogenes, with approximately 40.4% of patients with mCRC exhibiting *RAS* gene mutations. The *RAS* family comprises three genes: *KRAS*, *HRAS*, and *NRAS*. The *BRAF* (B-Rapidly Accelerated Fibrosarcoma) gene is a member of the RAF protein family, which encodes serine/threonine protein kinases, and plays a pivotal role in the mitogen-activated protein kinase (MAPK) signaling pathway, regulating cell growth, proliferation, and differentiation.

**
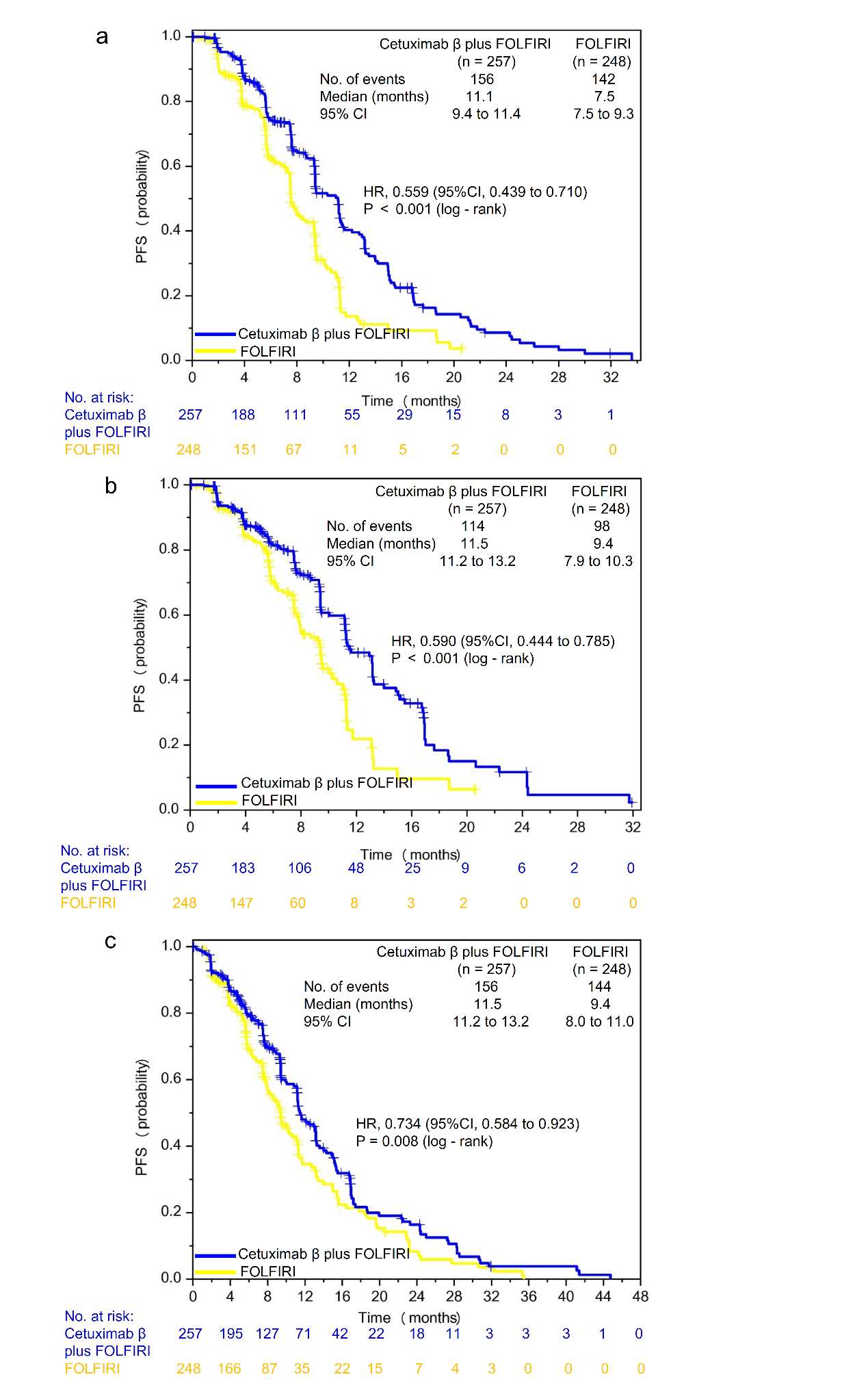
**

**Figure. S1. Sensitivity analyses of PFS.**

**a**, Kaplan-Meier estimates of investigator-assessed PFS in the FAS. **b,** Kaplan-Meier estimates of BIRC-assessed PFS in the FAS when progression of disease following the loss to follow-up of tumor assessment was considered as an event. **c,** Kaplan-Meier estimates of BIRC-assessed PFS in the FAS when progression of disease or death following the loss to follow-up of tumor assessment was considered as an event. Crosses denote censored patients. BIRC, blinded independent review committee; CI, confidence interval; FAS, full analysis set; FOLFIRI, irinotecan, fluorouracil, and leucovorin; HR, hazard ratio; PFS, progression-free survival.


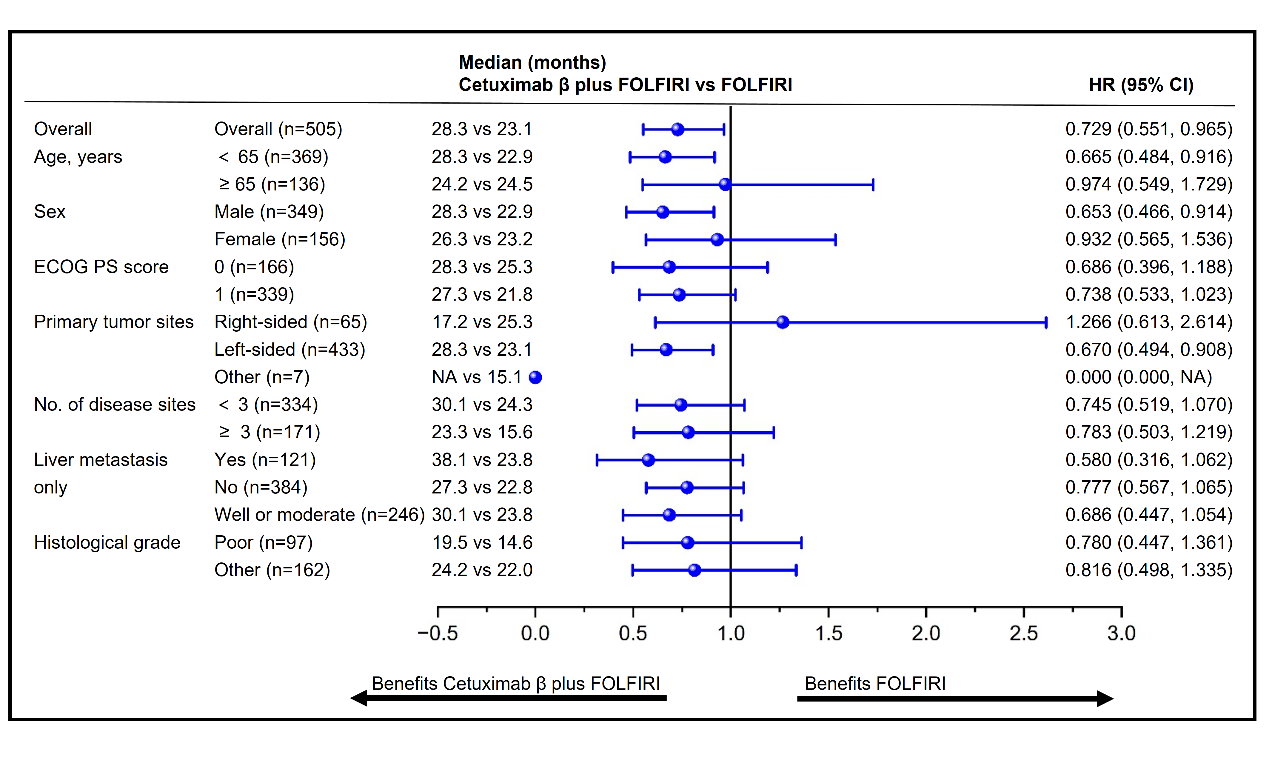


**Figure. S2. Subgroup analysis for OS in the FAS.**

CI, confidence interval; ECOG, Eastern Cooperative Oncology Group; FAS, full analysis set; FOLFIRI, irinotecan, fluorouracil, and leucovorin; HR, hazard ratio; OS, overall survival; PS, performance status.

**Table S1. Study drug exposure**

|  | Cetuximab β | | Irinotecan | | 5-Fluorouracil | |
| --- | --- | --- | --- | --- | --- | --- |
|  | Cetuximab β plus FOLFIRI  (n=257) | FOLFIRI  (n=248) | Cetuximab β plus FOLFIRI  (n=257) | FOLFIRI  (n=248) | Cetuximab β plus FOLFIRI  (n=257) | FOLFIRI  (n=248) |
| Duration of treatment, median (Q1, Q3), months | 7.3 (3.7, 11.0) | NA | 5.8 (3.4, 9.2) | 5.1 (2.3, 7.3) | 6.0 (3.4, 9.3) | 5.2 (2.4, 7.3) |
| RDI, mean (SD), % | 90.4 (11.4) | NA | 92.7 (12.1) | 94.9 (13.1) | 92.3 (12.2) | 93.2 (9.8) |
| RDI, n (%)  ＜60%  ≥60% to ＜80%  ≥80% to ＜90%  ≥90%  Missing | 3 (1.2)  40 (15.6)  58 (22.6)  156 (60.7)  0 (0) | NA | 1 (0.4)  35 (13.6)  41 (16.0)  179 (69.6)  1 (0.4) | 2 (0.8)  23 (9.3)  37 (14.9)  186 (75.0)  0 (0) | 4 (1.6)  31 (12.1)  45 (17.5)  176 (68.5)  1 (0.4) | 3 (1.2)  27 (10.9)  34 (13.7)  184 (74.2)  0 (0) |

FOLFIRI, irinotecan, fluorouracil, and leucovorin; NA, not applicable; Q1, first quantile; Q3, third quantile; RDI, relative dose intensity; SD, standard deviation.

**Table S2. Investigator-assessed PFS and ORR in the FAS**

| Efficacy | Cetuximab β plus FOLFIRI  (n=257) | FOLFIRI  (n=248) |
| --- | --- | --- |
| PFS | | |
| No. of events (PD or death), n (%)^1^ | 156 (60.7) | 142 (57.3) |
| Median (95% CI), months^2^ | 11.1 (9.4, 11.4) | 7.5 (7.5, 9.3) |
| HR (95% CI) | 0.559 (0.439, 0.710) |  |
| P value^3^ | <0.001 |  |
| Best overall response, n (%) | | |
| Complete response | 2 (0.9) | 0 (0.0) |
| Partial response | 138 (62.2) | 84 (37.2) |
| Stable disease | 61 (27.5) | 112 (49.6) |
| Progressive disease | 15 (6.8) | 29 (12.8) |
| Not evaluable | 6 (2.7) | 1 (0.4) |
| ORR, n (%) | 140 (63.1) | 84 (37.2) |
| 95% CI | 56.7, 69.4 | 30.9, 43.5 |
| OR (95% CI) | 2.890 (1.953, 4.276) |  |
| P value^3^ | <0.001 |  |

1 Death occurring within 90 days following the last tumor response evaluation or the date of randomization.

2 Kaplan-Meier estimates.

3 P values were calculated with the use of log-rank test or, in the case of ORR, Cochran–Mantel–Haenszel test.

CI, confidence interval; FAS, full analysis set; FOLFIRI, irinotecan, fluorouracil, and leucovorin; HR, hazard ratio; OR, odds ratio; ORR, objective response rate; PD, progressive disease; PFS, progression-free survival.
