# Supplementary material for: First-line treatment of anti-EGFR monoclonal antibody cetuximab β plus FOLFIRI versus FOLFIRI alone in Chinese patients with RAS/BRAF wild-type metastatic colorectal cancer: a randomized, phase 3 trial
Source: Signal Transduct Target Ther. 2025 May 7;10:147. doi: 10.1038/s41392-025-02229-4 (PMC12056184; doi:10.1038/s41392-025-02229-4)
Supplement: Supplementary file 2 — Study Protocol [file 41392_2025_2229_MOESM2_ESM.docx]

**Clinical Research Protocol**

| **DRUG** | CMAB009 |
| --- | --- |
| **STUDY NUMBER** | 009mCRCⅢP |
| **PROTOCOL TITLE** | A Phase III Open-Label, Randomized Controlled, Multicenter, Prospective Clinical Study of Recombinant Anti-EGFR Human-Mouse Chimeric Monoclonal Antibody Injection (CMAB009) Combined with FOLFIRI Chemotherapy Regimen versus FOLFIRI Chemotherapy Alone as First-Line Treatment for RAS/BRAF Wild-Type Metastatic Colorectal Cancer |
| **SPONSOR** | Taizhou Mabtech Pharmaceutical Co., Ltd. |
| **PRINCIPAL INVESTIGATOR** | Professor Yuankai Shi  Professor Yi Ba |
| **VERSION NUMBER** | 2.4 |
| **VERSION DATE** | 24 February 2022 |

| CONFIDENTIALITY STATEMENT |
| --- |
| All the information and materials contained in this document are the property of Taizhou Mabtech Pharmaceutical Co., Ltd., and are permitted to be reviewed by research team members, ethics committee members, and regulatory authorities under the premise of confidentiality. |

# Table of Content

[List of Abbreviations 5](#_Toc178517027)

[1 Synopsis 7](#_Toc178517028)

[2 Study Flowchart 15](#_Toc178517029)

[3 Introduction 19](#_Toc178517030)

[3.1 Nonclinical Studies 22](#_Toc178517031)

[3.2 Phase I Clinical Study 24](#_Toc178517032)

[3.3 Phase II/III Clinical Study 25](#_Toc178517033)

[3.4 Known and Potential Risks and Benefits to the Study Population 27](#_Toc178517034)

[4 Study Objectives 27](#_Toc178517035)

[5 Study Design 28](#_Toc178517036)

[5.1 Overall Study Design and Plan 28](#_Toc178517037)

[5.1.1 Pre-screening 28](#_Toc178517038)

[5.1.2 Screening Period 29](#_Toc178517039)

[5.1.3 Treatment Period 29](#_Toc178517040)

[5.1.4 Assessment Visits Every 8 Weeks 30](#_Toc178517041)

[5.1.5 Final Tumor Assessment (FTA) Visit 30](#_Toc178517042)

[5.1.6 End of Trial (EOT) Visit 30](#_Toc178517043)

[5.1.7 Long-term Follow-up Period 31](#_Toc178517044)

[5.1.8 Scientific Rationale for Study Design 31](#_Toc178517045)

[5.2 Selection of Study Population 32](#_Toc178517046)

[5.2.1 Diagnostic Criteria 32](#_Toc178517047)

[5.2.2 Definition of First-line Treatment Population 32](#_Toc178517048)

[5.2.3 Inclusion Criteria 32](#_Toc178517049)

[5.2.4 Exclusion Criteria 33](#_Toc178517050)

[5.3 Randomization 34](#_Toc178517051)

[5.4 Criteria for Subject Withdrawal 35](#_Toc178517052)

[5.4.1 Withdrawal from the Study 35](#_Toc178517053)

[5.4.2 Discontinuation of Study Treatment 35](#_Toc178517054)

[5.5 Premature Termination of Study Treatment 36](#_Toc178517055)

[5.6 Definition of Study Completion 37](#_Toc178517056)

[6 Study Medication 37](#_Toc178517057)

[6.1 Anti- EGFR Monoclonal Antibody 37](#_Toc178517058)

[6.1.1 Packaging of Anti-EGFR Monoclonal Antibody 37](#_Toc178517059)

[6.1.2 Preparation of Anti-EGFR Monoclonal Antibody Prior to Use 37](#_Toc178517060)

[6.1.3 Administration Schedule of Anti-EGFR Monoclonal Antibody 38](#_Toc178517061)

[6.1.4 Dose Adjustment of Anti-EGFR Monoclonal Antibody 38](#_Toc178517062)

[6.1.5 Precautions 39](#_Toc178517063)

[6.2 FOLFIRI Chemotherapy Regimen 41](#_Toc178517064)

[6.2.1 Composition of the FOLFIRI Chemotherapy Regimen 41](#_Toc178517065)

[6.2.2 FOLFIRI Administration Schedule 42](#_Toc178517066)

[6.2.3 Dose Adjustments for the FOLFIRI Regimen 42](#_Toc178517067)

[6.2.4 Precautions 45](#_Toc178517068)

[6.3 Medication Adherence 46](#_Toc178517069)

[6.4 Drug Management 46](#_Toc178517070)

[6.5 Concurrent Treatments 47](#_Toc178517071)

[6.5.1 Prohibited Concurrent Treatments 47](#_Toc178517072)

[6.5.2 Permitted Concurrent Treatments 47](#_Toc178517073)

[6.6 Management of Drug Overdose 48](#_Toc178517074)

[6.7 Resection of Metastatic Lesions 48](#_Toc178517075)

[6.8 Other Study Precautions 49](#_Toc178517076)

[7 Research Procedures and Evaluation 50](#_Toc178517077)

[7.1 Evaluation Schedule 50](#_Toc178517078)

[7.1.1 Pre-screening Visit 50](#_Toc178517079)

[7.1.2 Screening Visit 50](#_Toc178517080)

[7.1.3 Treatment Visit 52](#_Toc178517081)

[7.2 Clinical Observation Indicators During the Study 54](#_Toc178517082)

[7.3 Efficacy Assessment 55](#_Toc178517083)

[7.4 Safety Evaluation Criteria 63](#_Toc178517084)

[7.5 Adverse Events 63](#_Toc178517085)

[7.6 Pharmacokinetics 69](#_Toc178517086)

[7.7 Immunogenicity 70](#_Toc178517087)

[8 Data Quality Assurance 71](#_Toc178517088)

[8.1 Data Recording 71](#_Toc178517089)

[8.2 Data Monitoring 72](#_Toc178517090)

[9 Statistical Analysis 73](#_Toc178517091)

[9.1 Sample Size 73](#_Toc178517092)

[9.2 Endpoint Indicators 73](#_Toc178517093)

[9.3 Statistical Analysis Plan 74](#_Toc178517094)

[9.4 Analysis Datasets 75](#_Toc178517095)

[9.5 Statistical Analysis Methods 76](#_Toc178517096)

[9.6 Number of Clinical Trial Cases per Disease and the Rationale for Determination 78](#_Toc178517097)

[9.7 Statistical Procedures for All Data, Including Missing, Unused, or Erroneous Data (Including Dropouts and Withdrawals) and Unreasonable Data 78](#_Toc178517098)

[9.8 Procedures for Reporting Deviations from the Original Statistical Plan 79](#_Toc178517099)

[9.9 Exclusion of Special Information and the Rationale When Verifying Assumptions (If Applicable) 79](#_Toc178517100)

[10 Ethical Principles 79](#_Toc178517101)

[10.1 Investigator's Responsibilities 79](#_Toc178517102)

[10.2 Ethical Committee Approval 79](#_Toc178517103)

[10.3 Informed Consent 80](#_Toc178517104)

[10.4 Confidentiality of Subject Information 80](#_Toc178517105)

[11 Study Management 80](#_Toc178517106)

[11.1 Protocol Amendments 80](#_Toc178517107)

[11.2 Registration Documents 81](#_Toc178517108)

[11.3 Trial-Related Items 81](#_Toc178517109)

[11.4 Case Report Form Management 81](#_Toc178517110)

[11.5 Original Data and Subject Documentation 82](#_Toc178517111)

[11.6 Research Center Documentation and Archiving 83](#_Toc178517112)

[11.7 Use and Publication of Information 83](#_Toc178517113)

[12 References 84](#_Toc178517114)

[Appendix 86](#_Toc178517115)

[Appendix 1 Staging of Rectal and Colon Cancer (TNM Staging System) 86](#_Toc178517116)

[Appendix 2 The Common Terminology Criteria for Adverse Events (CTCAE) Version 4.03 by the National Cancer Institute (NCI) 88](#_Toc178517117)

[Appendix 3 Performance Status Scale (Eastern Cooperative Oncology Group) 91](#_Toc178517118)

[Appendix 4 New response evaluation criteria in solid tumors: Revised RECIST guideline (version 1.1) 92](#_Toc178517119)

[Appendix 5 EORTC Quality of Life Questionnaire - Core Questionnaire (QLQ-C30) 95](#_Toc178517120)

# List of Abbreviations

| Abbreviation | Full Name |
| --- | --- |
| 5-HT3 | 5-hydroxy tryptamine 3 |
| 5-FU | 5-fluorouracil |
| ADCC | Antibody Dependent Cell Mediated Cytotoxicity |
| ADR | Adverse Drug Reaction |
| ADA | Anti-drug antibody |
| AE | Adverse Event |
| AJCC | American Joint Committee on Cancer |
| AKP | Alkline phosphatase |
| ALT | Alanine aminotransferase |
| ANC | Absolute neutrophil count |
| AST | Asparate aminotransferase |
| BSA | Body surface area |
| CA19-9 | carbohydrate antigen 19-9 |
| CBR | Clinical benefit rate |
| CEA | carcino-embryonic antigen |
| CL | Clearance rate |
| CR | Complete remission |
| Cr | Creatinine |
| CRF | Case Report Form |
| CSCO | Chinese Society of Clinical Oncology |
| CT | Computed Tomography |
| CTC | Common toxicity criteria |
| DCR | Disease Control Rate |
| DNA | Deoxyribose Nucleic Acid |
| DOR | Duration of response |
| ECOG | Eastern Cooperative Oncology Group |
| ECT | Emission Computed Tomography |
| EDC | Electronic data capture |
| EGF | Epidermal Growth Factor |
| EGFR | Epidermal Growth Factor Receptor |
| EOT | End of treatment |
| FAS | Full Analysis Set |
| FDA | Food and Drug Administration |
| FTA | Final tumor assessment |
| GCP | Good Clinical Practice |
| G-CSF | Granulocyte colony-stimulating factor |
| Hb | Hemoglobin |
| HBV | hepatitis B virus |
| HIV | Human Immunodeficiency Virus |
| ITT | Intention to Treat |
| IRaC | Independent radiology review committee |
| LOCF | Last Observation Carry Forward |
| LV | leucovorin |
| mCRC | metastatic Colorectal Cancer |
| MedDRA | Medical Dictionary for Drug Regulatory Activities |
| MRI | Magnetic Resonance Imaging |
| MTD | Maximum Tolerated Dose |
| NCCN | National Comprehensive Cancer Network |
| NMPA | National Medical Products Administration |
| NYHA | New York Heart Association |
| ORR | Objective Response Rate |
| OS | Overall survival |
| PD | Progression of disease |
| PFS | Progression free survival |
| PK | Pharmacokinetic |
| PKPS | Pharmacokinetics Parameter Set |
| PLT | Platelets |
| PPS | Per-protocol set |
| PR | Partial remission |
| QOL | Quality of life |
| RECIST | Response Evaluation Criteria In Solid Tumors |
| SAE | Serious adverse event |
| SAS | Statistical analysis system |
| SCCHN | Squamous Cell Carcinoma of the Head and Neck |
| SS | Safety set |
| SUSAR | Suspected Unexpected Serious Adverse Reaction |
| TGF-α | Transforming growth factor-α |
| TTR | Time To Response |
| TBIL | Total bilirubin |
| UICC | International Union Against Cancer |
| WBC | White blood cell |

# 1 Synopsis

| **Sponsor** | Taizhou Mabtech Co., Ltd. |
| --- | --- |
| **Study Design** | An open-label, randomized controlled, multicenter, prospective Phase III clinical trial |
| **Study Title** | A phase III open-label, randomized controlled, multicenter, prospective clinical study of recombinant anti-EGFR human-mouse chimeric monoclonal antibody injection (CMAB009) combined with FOLFIRI chemotherapy regimen versus FOLFIRI chemotherapy alone as first-line treatment for RAS/BRAF wild-type metastatic colorectal cancer |
| **Study Objective** | To evaluate the efficacy and safety of the combination of recombinant anti-EGFR human-mouse chimeric monoclonal antibody injection (CMAB009) with the FOLFIRI (irinotecan, 5-fluorouracil, leucovorin) chemotherapy regimen as a first-line treatment for patients with RAS/BRAF wild-type, metastatic colorectal cancer, compared to the FOLFIRI regimen alone |
| **Indication** | First-line treatment for RAS/BRAF wild-type metastatic colorectal cancer |
| **Primary Efficacy Endpoint** | Progression-Free Survival (PFS) |
| **Secondary Efficacy Endpoints** | 1. Objective Response Rate (ORR) 2. One-year Overall Survival, Two-year Overall Survival, and Overall Survival (OS) 3. Disease Control Rate (DCR) 4. Clinical Benefit Rate (CBR) 5. Duration of Response (DOR) 6. Time to Response (TTR) 7. Quality of Life (EORTC-QLQ-C30) 8. Rate of Radical Hepatic Metastasectomy |
| **Other Secondary Endpoints** | 1. Pharmacokinetics of CMAB009 2. Immunogenicity Study of CMAB009 (ADA Detection) |
| **Safety Endpoints** | 1. Monitoring of Vital Signs, Physical Examination, and Laboratory Test Indicators 2. Recording of Serious Adverse Events/Adverse Events and Their Incidence After Informed Consent |
| **Study Procedure** | Eligible subjects will be stratified by ECOG performance status and study site, and then randomly assigned in a 1:1 ratio according to the order of enrollment, to receive either the recombinant anti-EGFR human-mouse chimeric monoclonal antibody injection (CMAB009) in combination with the FOLFIRI chemotherapy regimen or the FOLFIRI chemotherapy regimen alone. Treatment will be administered in cycles of 2 weeks. Tumor efficacy will be assessed every 8 weeks during the treatment period based on the Response Evaluation Criteria in Solid Tumors (RECIST version 1.1). Treatment will be discontinued and follow-up initiated once progressive disease (PD) is reached or if the criteria for study withdrawal are met. Quality of life will be assessed using the EORTC QLQ-C30, with assessments conducted at baseline prior to treatment, and then every 8 weeks thereafter until the start of follow-up.  After treatment completion, subjects will be followed up every 3 months for survival status, adverse events (AEs) present at the end of treatment, and skin reactions. |
| **Planned Enrollment** | The study plans to enroll 512 subjects with mCRC. The group treated with recombinant anti-EGFR human-mouse chimeric monoclonal antibody injection (CMAB009) in combination with the FOLFIRI chemotherapy regimen (Group A) and the group treated with the FOLFIRI chemotherapy regimen alone (Group B) are designed in a 1:1 ratio. Considering a 20% dropout rate, the actual required enrollment is 256 subjects for the experimental group and 256 subjects for the control group. |
| **Planned Study Duration** | The study is set to commence in December 2017 and is expected to last for 4-5 years.  Recruitment Period: December 2017 to March 2021 (39 months) |
| **Study Centers** | Approximately 60 centers |
| **Evaluation Schedule** | **Pre-screening Visit:**   - Signing of the written informed consent form - Subject number assignment - Diagnosis of tumor - Archival tumor specimens required for RAS/BRAF testing   **Screening Visit:**   - Inclusion and exclusion criteria - Demographic data - Medical history collection - Diagnosis of the primary tumor - Concurrent treatments and current medical interventions - Assessment of previous treatments - Tumor markers (CEA and CA19-9) - Baseline tumor assessment with CT or MRI scans - EORTC QLQ-C30 questionnaire - Physical examination - Vital signs - ECOG score - 12-lead electrocardiogram (ECG) - Routine blood test, routine urine test, hepatic and renal function, electrolyte examination - HIV antibody test - Hepatitis B panel test and/or HBV-DNA test - Pregnancy test (if applicable) - Random grouping - Assessment of adverse events - Collection of blood samples for ADA analysis (applicable only to Group A)   Acceptable recent results of routine blood, urine, hepatic and renal function, electrolytes, and ECG within 7 days prior to randomization as baseline assessment.  Acceptable baseline tumor evaluation using CT or MRI imaging within 4 weeks prior to the first administration of medication.  **Treatment Visit:**  **Day 1 of Each Treatment Cycle**   - Weight measurement and calculation of BSA (within 3 days prior to administration) - Administration of anti-EGFR monoclonal antibody (Group A only) - Administration of chemotherapy (all subjects) - 12-lead electrocardiogram (ECG) (tested once every 2 weeks for the first three treatment cycles, then once every 4 weeks; on the day of administration or within 3 days prior) - Routine blood and urine tests, hepatic and renal function, and electrolytes (within 3 days prior to administration) - Concurrent medications and current medical interventions - Assessment of adverse events - PK blood sample collection before administration (for subjects selected for PK testing)   **Day 8 of Each Treatment Cycle (Group A only)**   - Administration of anti-EGFR monoclonal antibody (Group A only) - Routine blood test (within 3 days prior to administration) - Concurrent medications and current medical interventions - Assessment of adverse events - PK blood sample collection before administration (for subjects selected for PK testing)   **Evaluation Visit Every 8 Weeks:**   - Weight measurement - CT or MRI scans for tumor efficacy assessment - EORTC QLQ-C30 questionnaire - CEA and CA19-9 - Vital signs - Physical examination - 12-lead electrocardiogram (ECG) - Routine blood and urine tests, hepatic and renal function, and electrolytes - Concurrent medications and current medical interventions - Assessment of adverse events - ECOG score   **Final Tumor Assessment Visit:**   - CT or MRI scans for tumor efficacy assessment - EORTC QLQ-C30 questionnaire - CEA and CA19-9 - Vital signs - Physical examination - 12-lead electrocardiogram (ECG) - Routine blood and urine tests, hepatic and renal function, and electrolytes - Concurrent medications and current medical interventions - Assessment of adverse events - ECOG score   **End-of-Trial Visit:**   - Items included in the end-of-trial assessment: completion date, last treatment date, subject's status at study end, reason for treatment discontinuation, and whether the disease progressed at the EOT visit - Concurrent medications and current medical interventions - Collection of blood samples for ADA analysis (Group A only) - Assessment of adverse events   **Follow-up:**   - Collection of tumor imaging data - Subject's survival status - Subsequent anti-tumor treatments - Skin toxicity still present at the EOT visit - Assessment of adverse events |
| **Inclusion Criteria** | 1. Age between 18 and 75 years, regardless of gender; 2. Histologically confirmed adenocarcinoma of the colon or rectum; 3. First occurrence of metastatic lesions (unresectable for cure); 4. Tumor tissue with wild-type RAS/BRAF genes; 5. At least one measurable lesion according to RECIST 1.1 criteria confirmed by CT or MRI (outside of a radiation therapy field); 6. ECOG performance status of 0 to 1; 7. Life expectancy of more than 3 months with a residence conducive to follow-up; 8. Subjects of childbearing potential must undergo effective medical contraception (for both male and female subjects until 90 days after the last study drug administration); 9. Recovery from any toxicities related to previous treatments before study enrollment; 10. Willingness to sign an informed consent form. |
| **Exclusion Criteria** | 1. Radiation therapy or surgery (except for diagnostic biopsy) within 30 days before the start of treatment; 2. Organ function levels as follows:  - Hematopoietic system: White blood cells (WBC) <3.0×10^9^/L; Neutrophils (ANC) <1.5×10^9^/L; Platelets (PLT) <100×10^9^/L; Hemoglobin (Hb) <90g/L; - Liver: Total bilirubin (TBIL) >1.5 times the upper limit of normal reference range; Alanine aminotransferase and aspartate aminotransferase (ALT and AST) >2.5 times the upper limit of normal reference range (for those without liver metastasis) or >5 times the upper limit of normal reference range (for those with liver metastasis); - Kidneys: Serum creatinine (Cr) >1.5 times the upper limit of normal or creatinine clearance <50mL/min;  1. Previous adjuvant or neoadjuvant chemotherapy for CRC with a chemotherapy-free interval of less than 12 months from the discovery of disease recurrence or metastasis; 2. Previous exposure to anti-EGFR monoclonal antibodies, EGFR tyrosine kinase inhibitors, or other EGFR-targeted therapies (e.g., cetuximab, nimotuzumab, panitumumab, etc.); 3. Known hypersensitivity or allergic reactions to any component of the study treatment; 4. History of organ transplantation, autologous/allogeneic stem cell transplantation; 5. Other concurrent antineoplastic treatments (forbidden concomitant treatments detailed in Section 6.5.1); 6. Symptomatic brain and/or leptomeningeal metastases; 7. History of other malignant tumors within the last 5 years, except for carcinoma in situ of the cervix, basal cell carcinoma of the skin, or squamous cell carcinoma; 8. Participation in other clinical trials of antineoplastic drugs within 30 days before enrollment; 9. Receiving chronic systemic immunotherapy or hormone therapy other than physiological replacement therapy; 10. Any unstable systemic disease:   Including active infection, uncontrolled hypertension, unstable angina, onset of angina within the last 3 months, congestive heart failure (New York Heart Association [NYHA] Class ≥ II), myocardial infarction within 6 months before enrollment, severe arrhythmias requiring medication, liver, kidney, or metabolic diseases;   1. Acute or subacute intestinal obstruction, or a history of inflammatory bowel disease; 2. Subjects with severe bone marrow failure; 3. Any disease, metabolic disorder, or medical examination or laboratory findings that suggest the patient has contraindications to the study drug or is at high risk for treatment complications; 4. Known or self-reported human immunodeficiency virus (HIV) infection; 5. Hepatitis B virus DNA positive (copy number greater than 103); 6. Pregnancy or lactation; 7. Known alcohol or drug addiction, or other poor health conditions or mental states that may affect protocol compliance and evaluation of trial endpoints, as determined by the investigator, making the subject unsuitable for the study; 8. Lack of legal capacity or limited legal capacity. |
| **Study Medication** | **Experimental Group:**  Recombinant anti-EGFR human-mouse chimeric monoclonal antibody injection combined with FOLFIRI chemotherapy  Recombinant anti-EGFR human-mouse chimeric monoclonal antibody injection (CMAB009):  The initial dose is 400 mg/m^2^, administered intravenously over at least 120 minutes; the maintenance dose is 250 mg/m^2^, administered intravenously over at least 60 minutes, once weekly.  FOLFIRI chemotherapy regimen:  Irinotecan 180 mg/m^2^, administered intravenously over 30 to 90 minutes, on Day 1; Leucovorin (LV) 400 mg/m^2^, should be administered immediately after the infusion of Irinotecan, with a maximum interval not exceeding 30 minutes, with the same infusion time, on Day 1; 5-Fluorouracil (5-FU), 400 mg/m^2^, administered by intravenous bolus or rapid intravenous infusion, on Day 1; followed by a continuous intravenous infusion of 1,200 mg/m^2^/day × 2 days (total 2,400 mg/m^2^, recommended infusion over 46 to 48 hours), to be repeated every 2 weeks.  **Control Group:**  FOLFIRI chemotherapy alone  FOLFIRI chemotherapy regimen:  Irinotecan 180 mg/m^2^, administered intravenously over 30 to 90 minutes, on Day 1; Leucovorin (LV) 400 mg/m^2^, should be administered immediately after the infusion of Irinotecan, with a maximum interval not exceeding 30 minutes, with the same infusion time, on Day 1; 5-Fluorouracil (5-FU), 400 mg/m^2^, administered by intravenous bolus or rapid intravenous infusion, on Day 1; followed by a continuous intravenous infusion of 1,200 mg/m^2^/day × 2 days (total 2,400 mg/m^2^, recommended infusion over 46 to 48 hours), to be repeated every 2 weeks. |
| **Estimated Duration of Treatment per Subject** | Subjects will receive treatment until disease progression, intolerable toxicity, or withdrawal of informed consent. Consequently, the duration of treatment may vary among subjects |
| **Statistical Methods** | Statistical analysis will be conducted using SAS 9.4 or later version statistical analysis software.  All statistical tests in this study will be two-sided, and a P-value less than 0.05 will be considered to indicate a statistically significant difference. Descriptive statistics will be the primary method of analysis, utilizing measures such as mean, standard deviation, median, minimum, and maximum values for continuous variables; counts and percentages for categorical variables.   1. Baseline Analysis   Baseline analysis will utilize the Full Analysis Set (FAS) dataset. Inter-group comparisons of baseline indicators such as demographic characteristics and medical history will be performed using t-tests or Wilcoxon rank-sum tests for continuous data, and Fisher's exact test for categorical data. Adverse events will be coded using Medical Dictionary for Regulatory Activities (MedDRA) terminology.   1. Analysis of Enrollment and Excluded Cases   The analysis will primarily be descriptive, detailing the enrollment and completion of cases at each center, as well as the distribution of dropouts and excluded cases. The demographic distribution of the study population at each center will also be described in tables.   1. Efficacy Analysis   PFS: analysis of PFS between the two groups will utilize the Log-rank test, stratified by center and ECOG score as covariates. The Kaplan-Meier method will be utilized to estimate the median Progression-Free Survival (PFS) and its 95% confidence interval (CI), with the corresponding survival curves plotted. The Cox proportional hazards model will be employed to estimate the hazard ratio (HR) and its 95% CI.  For ORR, one-year overall survival rate, two-year overall survival rate, DCR, and CBR, a Logistic regression model will be used for analysis, with covariates (center, ECOG score) adjusted to calculate the odds ratio and its 95% confidence interval for each group.  For DOR and OS, the survival function will be estimated using the Kaplan-Meier method, and survival curves will be plotted. The Log-rank test will be performed between the two groups. The Cox proportional hazards model will be used to calculate the hazard ratio and its 95% confidence interval between treatment groups, with covariates (center, ECOG score) specified.  For TTR, only subjects who have achieved a response and have no censored data will be included. The analysis will be conducted as a continuous variable using a linear regression model, with covariates (center, ECOG score) adjusted to calculate the difference and its 95% confidence interval between the two groups.  For Quality of Life Evaluation scores, comparisons between the two groups will be made using t-tests or Wilcoxon rank-sum tests.   1. Safety Analysis   The Safety Set (SS) dataset will be used, with a focus on descriptive statistical analysis. Adverse events, laboratory tests, and vital signs will be listed and described, along with the determination of their association with the trial medication. Where necessary, inter-group comparisons will be made using t-tests or Fisher's exact test.   1. Other Analyses   Corresponding statistical analyses will be conducted based on PK/PD and ADA data. |

# 2 Study Flowchart

|  | **Pre-treatment** | | **Chemotherapy (14 days per cycle)** | | **Evaluation Visit (very 8 weeks) ^2^** | **FTA ^3^** | **EOT ^4^** | **Follow-up (every 3 months)** |
| --- | --- | --- | --- | --- | --- | --- | --- | --- |
|  | **Pre-screening ^1^** | **Screen** |  |  |  |  |  |  |
|  | **>-14 d** | **—14 d** | **D1** | **D8** |  |  |  |  |
| **Informed consent** | **√** |  |  |  |  |  |  |  |
| **RAS/BRAF testing** | **√** |  |  |  |  |  |  |  |
| **Diagnosis of tumor** | **√** |  |  |  |  |  |  |  |
| **Subject number assignment** | **√** |  |  |  |  |  |  |  |
| **Demographic data** |  | **√** |  |  |  |  |  |  |
| **Inclusion and exclusion criteria** |  | **√** |  |  |  |  |  |  |
| **Medical history and previous treatments** |  | **√** |  |  |  |  |  |  |
| **Physical examination** |  | **√** |  |  | **√** | **√** |  |  |
| **Vital signs** |  | **√** |  |  | **√** | **√** |  |  |
| **ECOG score** |  | **√** |  |  | **√** | **√** |  |  |
| **Routine blood test ^5^** |  | **√** | **√** | **√** | **√** | **√** |  |  |
| **Routine urine test ^5^** |  | **√** | **√** |  | **√** | **√** |  |  |
| **Hepatic and renal function ^5^** |  | **√** | **√** |  | **√** | **√** |  |  |
| **Hepatitis B panel test and/or HBV-DNA test** |  | **√** |  |  |  |  |  |  |
| **HIV antibody test** |  | **√** |  |  |  |  |  |  |
| **Electrolyte test** **^5^** |  | **√** | **√** |  | **√** | **√** |  |  |
| **ECG ^6^** |  | **√** | **√** |  | **√** | **√** |  |  |
| **Pregnancy test (if applicable)** |  | **√** |  |  |  |  |  |  |
| **Tumor assessment (radiography)** |  | **√** |  |  | **√** | **√** |  | **√^16^** |
| **EORTCQLQ-C30 questionnaire** |  | **√** |  |  | **√** | **√** |  |  |
| **Randomization** |  | **√** |  |  |  |  |  |  |
| **Weight and BSA ^7^** |  |  | **√** |  | **√** |  |  |  |
| **Administration of anti-EGFR monoclonal antibody ^8^** |  |  | **√** | **√** |  |  |  |  |
| **Administration of FOLFIRI chemotherapy ^9^** |  |  | **√** |  |  |  |  |  |
| **CEA and CA19-9 test ^10^** |  | **√** |  |  | **√** | **√** |  |  |
| **Blood samples for ADA ^11^** |  |  | **√** |  |  |  | **√** |  |
| **Blood samples for PK ^12^** |  |  | **√** | **√** |  |  |  |  |
| **Assessment of adverse events ^13^** |  | **√** | **√** | **√** | **√** | **√** | **√** |  |
| **Concurrent medications and current medical interventions ^14^** |  | **√** | **√** | **√** | **√** | **√** | **√** |  |
| **Survival status** |  |  |  |  |  |  |  | **√** |
| **Subsequent anti-tumor treatments** |  |  |  |  |  |  |  | **√** |
| **Skin toxicity still present at the EOT visit ^15^** |  |  |  |  |  |  |  | **√** |

Note: Cranial CT or MRI, and bone ECT should be performed in subjects suspected of having brain or bone metastases; routine blood tests, hepatic and renal function tests, and imaging examinations of the thorax, abdomen, and pelvis may be appropriately increased if deemed necessary by the investigator.

The treatment start window for each cycle is ±3 days; the visit start window for each visit cycle is ±3 days.

Recent results of routine blood tests, routine urine tests, hepatic and renal function tests, electrolytes, and ECG within 7 days prior to randomization are acceptable for baseline evaluation.

CT or MRI imaging within 4 weeks prior to the first administration of medication is acceptable for baseline tumor evaluation.

1: In the pre-screening process, tumors must be confirmed as metastatic colorectal cancer and both RAS/BRAF genotypes must be wild-type to proceed to the screening phase.

2: Evaluation visit intervals are conducted every 8 weeks until study withdrawal.

3: The final tumor assessment (FTA) visit is conducted after all study treatments have been completed.

4: The end-of-trial (EOT) visit must be conducted before the initiation of new anti-tumor treatment or at least 30 days after the final tumor assessment.

5: Within 3 days prior to administration.

6: Electrocardiogram (ECG) examinations are conducted once every 2 weeks during the first 3 cycles and once every 4 weeks thereafter (on the day of administration or within 3 days prior to administration).

7: Body weight is measured and BSA (Body Surface Area) is calculated on the first day of each treatment cycle. If a subject's body weight increases or decreases by more than 5% compared to the weight at the last dose adjustment, drug dosage adjustment is required; otherwise, no dosage adjustment is needed (within 3 days prior to administration).

8: Only applicable to Group A, the investigator must closely monitor the subject's condition during and for at least 1 hour after the infusion process.

9: Chemotherapy continues on the second day of the treatment cycle.

10: Tumor marker tests are conducted in conjunction with the evaluation visit, i.e., once every 8 weeks.

11: Only applicable to Group A, blood samples are collected before research treatment on the first week, and at weeks 4, 8, 16, 32, and the end-of-study visit, totaling 6 blood collections.

12: Only applicable to specific subjects, the flowchart does not indicate a clear blood collection time point. For detailed information, refer to Section 7.6.

13: Adverse events need to be continuously recorded until symptom resolution or outcome is predictable.

14: Concurrent medications and current medical interventions need to be continuously recorded until the end of the study.

15: Skin toxicity present at the EOT visit will be followed up for a maximum of 6 months.

16: During the follow-up period, if a subject discontinues treatment for reasons other than disease progression or withdrawal of informed consent, efforts should be made to collect tumor imaging data for tumor assessment until disease progression or initiation of other anti-tumor treatments (whichever occurs first).

# 3 Introduction

Colorectal cancer is one of the most prevalent malignant tumors, with nearly 1.4 million new cases annually worldwide and nearly 700,000 patient deaths ^[1]^. In China, the incidence and mortality rates of colorectal cancer rank fifth among all malignant tumors, and the incidence shows a continuous increasing trend ^[2]^. In the early stages of the disease, surgery alone can cure the condition, but due to the usually non-specific early symptoms, it is difficult to diagnose, with approximately 20% of patients being diagnosed for the first time with stage IV metastatic colorectal cancer (mCRC). Overall, the five-year survival rate for colorectal cancer patients is as high as 65%, but the five-year survival rate for patients with metastatic colorectal cancer (mCRC) is less than 15% ^[3]^.

Similar to other epithelial cell-derived malignant tumors, which account for about 70%, colorectal cancer tumor cells overexpress a protein called epidermal growth factor receptor (EGFR) ^[4]^. Typically, it can be activated by a series of endogenous factors such as epidermal growth factor (EGF) or TGF-α, activating a series of downstream cascade enzymatic signal transduction pathways, transmitting growth signals to the nucleus, promoting the proliferation, migration, invasion of tumor cells, and neovascularization, and inhibiting tumor cell apoptosis ^[5-7]^. Tumor patients with overexpressed EGFR often have a poorer prognosis ^[8]^. Therefore, it can be speculated that if there are drugs that can block the activity of EGFR, it will inhibit its signal transduction, thereby playing a multifaceted role in anti-tumor activity, and can also synergistically enhance the anti-tumor efficacy of chemotherapy and radiotherapy.

Anti-EGFR human-mouse chimeric monoclonal antibody (Cetuximab) is an example of such a targeted drug, which binds to the extracellular domain of EGFR, thereby blocking cellular signal transduction and slowing the growth rate of tumors. Additionally, as Cetuximab is an IgG1 antibody, it can also induce antibody-dependent cell-mediated cytotoxicity (ADCC), leading to the death of tumor cells ^[11]^. In the early clinical application phase of monoclonal antibodies targeting EGFR, one of the routine detection items for selecting patients for medication is the detection of EGFR expression by immunohistochemistry. However, previous clinical basic research has shown that the expression of EGFR in tumor tissue does not have a clear correlation with the clinical efficacy of Cetuximab ^[12]^. The mutational status of RAS genes, which are related to the downstream signal transduction pathway of EGFR, has attracted increasing interest among researchers, as mutations in RAS genes can lead to poor efficacy of Cetuximab in the treatment of metastatic colorectal cancer ^[13-14]^.

In December 2003, the anti-epidermal growth factor receptor (EGFR) human-mouse chimeric monoclonal antibody (trade name Erbitux^®^) was first launched in Switzerland. On February 12, 2004, the U.S. FDA also approved the marketing application of this product, indicated for use in combination with irinotecan for the treatment of EGFR-expressing, irinotecan-refractory metastatic colorectal cancer (mCRC). Subsequently, Erbitux^®^ was approved in over 50 countries and regions, including Canada and Australia, for the treatment of EGFR-expressing mCRC that has failed irinotecan chemotherapy and is intolerant to irinotecan. Erbitux^®^ has also been approved in several countries, including the United States, for the treatment of squamous cell carcinoma of the head and neck (SCCHN) either as a single agent or in combination with radiotherapy. In 2009, the FDA added new information to the product label of Erbitux^®^, stating that it is ineffective for colorectal cancer patients with KRAS mutations. In July 2012, the FDA approved a KRAS gene detection kit as a companion diagnostic for Erbitux^®^, and also approved Erbitux^®^ in combination with FOLFIRI (irinotecan + leucovorin + 5-fluorouracil) as a first-line treatment for KRAS wild-type, EGFR-expressing mCRC.

In December 2005, China's National Medical Products Administration (NMPA) approved the launch of Erbitux^®^ in China (Chinese trade name: 爱必妥^®^, import registration number: S20050095), indicated for the treatment of EGFR-expressing mCRC that has failed irinotecan chemotherapy, either as a single agent or in combination with irinotecan. In September 2019, the NMPA approved爱必妥^®^ in combination with FOLFOX (oxaliplatin + leucovorin + 5-fluorouracil) or FOLFIRI as a first-line treatment for RAS gene wild-type mCRC patients. 爱必妥^®^ in combination with platinum-based chemotherapy and 5-fluorouracil for the first-line treatment of recurrent and/or metastatic SCCHN was approved by the FDA in November 2011 and by the NMPA in March 2020.

An early large-scale clinical trial (CRYSTAL ^[15]^) was conducted in 1,198 subjects, randomly divided into a 1:1 ratio of FOLFIRI plus cetuximab group (599 cases) and FOLFIRI control group (599 cases). The results showed a difference in median progression-free survival (mPFS) between the two groups, but it was not satisfactory: 8.9 months vs. 8.0 months, P=0.048, while the 1-year disease-free survival rate and objective response rate showed a more significant difference between the two groups: 34% vs. 23%, 46.9% vs. 38.7% (P=0.0038). However, the trial did not screen for KRAS gene mutations at the initial design stage. Some research results have shown that mutations in codons 12 and 13 of KRAS are closely related to its efficacy ^[16-17]^. Subsequently, Professor Van Cutsen analyzed the KRAS mutation status (codons 12 and 13) and clinical efficacy in tumor tissue specimens of 1,063 subjects in the CRYSTAL trial ^[18]^, and the clinical characteristics and treatment outcomes of the selected subjects were similar to the entire Intent-to-Treat (ITT) population, of which 666 (62.7%) were wild-type and 397 (37.3%) were mutant-type. Wild-type patients treated with cetuximab plus FOLFIRI showed significantly better efficacy than the FOLFIRI group alone: objective response rate (ORR) 57.3% vs. 39.7%, mPFS 9.9 months vs. 8.4 months, median overall survival (mOS) 23.5 months vs. 20.0 months, with significant differences between the two groups (P<0.05). For mutant-type KRAS, the combination of cetuximab and chemotherapy did not enhance the efficacy of chemotherapy. The reason is that after the KRAS gene mutation, the Ras protein does not need to be activated by the upstream EGFR and can remain active continuously, so using cetuximab to block EGFR cannot effectively inhibit the activation of the downstream Ras protein. Therefore, cetuximab is ineffective when KRAS is mutated. KRAS mutation can be considered as one of the important factors to predict the efficacy of cetuximab. Given that 30-45% of colorectal cancer subjects carry the KRAS mutation gene, experts recommend KRAS gene mutation testing as an important screening check before cetuximab treatment to select the most effective subjects and save unnecessary medical expenses.

With further research, in 2015, Professor Van Cutsen further examined the benefit of RAS gene wild-type subjects in 666 KRAS wild-type patients ^[18]^. The results showed ^[19]^: patients with RAS gene wild-type (KRAS exons 2, 3, 4, and NRAS exons 2, 3, 4) treated with cetuximab plus FOLFIRI showed significantly better efficacy than the FOLFIRI group alone: ORR 66.3% vs. 38.6%, mPFS 11.4 months vs. 8.4 months, mOS 28.4 months vs. 20.2 months, with significant differences between the two groups (P<0.05), while in the mutant-type population, there was no difference between the two treatment groups, and there was even a trend of decline. However, there was no significant difference in adverse reactions between wild-type and mutant-type. The National Comprehensive Cancer Network (NCCN) 2017 v1 recommended that patients should consider the mutation status of the RAS gene when receiving cetuximab treatment, and only patients with RAS (KRAS/NRAS) wild-type could benefit from the treatment. Professor Van Cutsen retrospectively analyzed whether BRAF mutation in KRAS wild-type patients in the CRYSTAL trial ^[20]^, and the results showed that, under the premise of KRAS wild-type, patients with BRAF wild-type had more significant benefits in mPFS. The mPFS of the (Cetuximab+FOLFIRI) group and the FOLFIRI group were 10.9 months and 8.8 months, respectively, P=0.0013. However, for patients with BRAF mutant-type, the mPFS of the (Cetuximab+FOLFIRI) group and the FOLFIRI group were 8.0 months and 5.6 months, respectively, P=0.87. Another retrospective study showed that, in patients with KRAS wild-type and BRAF mutant-type, this genotype had no significant effect on ORR (P=0.063), but it could significantly shorten PFS (P<0.001) and OS (P<0.001) ^[21]^. Mutations in other gene sites such as PIK3CA and PTEN ^[22-23]^ still need to be verified by large-sample trials.

Since the launch of 爱必妥^®^ in China, its outstanding performance in the treatment of colorectal cancer has been recognized by a large number of doctors and subjects, but its data in combination with FOLFIRI are relatively limited, and no large-scale clinical trials have been conducted in China. At the same time, there is also a need for more affordable domestic anti-EGFR monoclonal antibodies targeting the same point to be put on the market, providing patients with more and more beneficial choices.

The anti-EGFR monoclonal antibody (CMAB009) developed by our company, its main component is a recombinant protein prepared by DNA recombinant technology and CHO eukaryotic expression system. It is a recombinant human-mouse chimeric monoclonal antibody targeting the epidermal growth factor receptor (EGFR), belonging to the IgG1 kappa antibody, composed of the Fv region of the mouse anti-human EGFR monoclonal antibody (mab225) and the constant regions of the human IgG1 heavy and light chains, with a molecular weight of about 152kDa. In June 2007, the State Food and Drug Administration approved the phase III clinical trial of the anti-EGFR monoclonal antibody (code name "CMAB009") developed by our company according to the new drug requirements (drug clinical research approval number: 2007L02499), for KRAS gene wild-type metastatic colorectal cancer patients who are ineffective to oxaliplatin and fluorouracil class drugs, the product shows clear efficacy and good tolerance. Due to the change of production site, it is now necessary to carry out bridging experiments to further verify its effectiveness and safety.

## 3.1 Nonclinical Studies

**Primary Pharmacodynamic Studies:** In vitro studies have shown that CMAB009 can inhibit the proliferation of the human epidermoid carcinoma cell line A431 and the human renal carcinoma cell line ACHN (both express EGFR) mediated by low concentrations of EGF. It can also inhibit the proliferation inhibition effect of A431 mediated by high concentrations of EGF, with inhibitory activity consistent with that of Erbitux^®^. CMAB009 can mediate the ADCC effect of peripheral blood mononuclear cells on A431 cells, with activity consistent with that of Erbitux^®^. The affinity constant was determined by fluorescence competitive binding assay, with the results showing that the EC50 value of CMAB009 is 10.94 µg/mL, and the affinity constant is 0.52 nM; the EC50 value of Erbitux^®^ is 10.92 µg/mL, and the affinity constant is 0.39 nM, indicating that the antigen affinity of the two is essentially the same. In vivo experimental results showed that the tumor growth rate of the treated animals in both CMAB009 and Erbitux^®^ groups was halted, with some animals experiencing tumor regression. By the end of the observation period, all animals were alive, with no statistical difference between the two groups.

**General Pharmacology Studies:** The intravenous administration of CMAB009 at doses of 30, 100, and 300 mg/kg via the tail vein had no significant effect on the general behavior of ICR mice; the 30 mg/kg dose had no effect on the spontaneous activity of mice, while the 100 and 300 mg/kg doses had certain effects. A single intravenous infusion of this product at doses of 10, 30, and 100 mg/kg had certain effects on the respiratory rate and amplitude, systolic and diastolic blood pressure, heart rate, rhythm, and electrocardiogram of anesthetized crab-eating macaques, but there were no significant differences compared to before administration, and all fluctuations were within the normal permissible range, thus these changes were considered to have no particular physiological significance.

**In Vivo Pharmacokinetic Studies in Animals:**

①Single-dose administration: Following a single intravenous dose (vd) of 7.0, 21.0, and 63.0 mg•kg^-1^ of CMAB009, the time to reach peak serum antigen concentration (T_max_, actual value) was 2.0±1.7, 1.7±2.0, and 1.0±0.0 hours, respectively. The mean peak serum antigen concentrations (C_max_) for each single-dose group were 75±13 μg•mL^-1^, 271±152 μg•mL^-1^ (P<0.05 compared to the low-dose group), and 676±121 μg•mL^-1^ (P<0.01 compared to both the low and medium-dose groups). The AUC_(0-120 h)_ after doses of 7.0, 21.0 (first time), and 63.0 mg•kg^-1^ were 2174±66 μg•h•mL^-1^, 7012±2480 μg•h•mL^-1^ (P<0.05 compared to the low-dose group), and 27003±1994 μg•h•mL^-1^ (P<0.01 compared to both the low and medium-dose groups), respectively, with a significant increase in AUC as the dose increased. The ratio of low, medium, and high doses was 1:3:9, and the increase in AUC_(0-120 h)_ was 1:3.2:12.4.

②Multiple-dose administration: In cynomolgus monkeys, a weekly dose of 21.0 mg•kg^-1^ of CMAB009 for four consecutive administrations showed that the pharmacokinetic parameters and the comparison between the first and fourth administrations indicated that the trough concentration (C_min_) before the fourth dose was significantly higher than before the first dose (32.11±18.07 µg•mL^-1^ vs 0.49±0.19 µg•mL^-1^, P<0.05). Except for the first sampling point after administration, there was no statistically significant difference in blood concentrations at other time points compared to the same time points after the first injection. There were no statistically significant differences in pharmacokinetic parameters, and the AUC_(0-120 h)_ after the first and fourth injections were 7012±2480 g•h•mL^-1^ and 5503±1473 g•h•mL^-1^, respectively (P=0.2081). No drug accumulation was observed in the body after four consecutive weekly doses of 21.0 mg•kg^-1^. The calculated terminal half-life after the fourth dose in the continuous administration group was 122.7±38.1 hours, which was not statistically significant compared to the single-dose groups at low and high doses.

**Preclinical Toxicology Studies:**

①Acute toxicity test: The maximum tolerated dose (MTD) in rats via tail vein injection (i.v.) was >1000 mg/kg (equivalent to 100 times the recommended clinical dose for humans); the MTD in mice after i.v. administration was >2500 mg/kg (equivalent to 250 times the recommended clinical dose for humans).

②Subchronic toxicity test: After 13 weeks of continuous intravenous infusion, the main toxic effects on crab-eating macaques were pathological changes in the skin and other squamous epithelia, as well as bacteremia due to local infections at the lesion sites, and secondary infections in the kidneys, liver, etc. These changes were all related to CMAB009 and were reversible, with alleviation and gradual recovery after discontinuation of the drug, mainly due to the amplified pharmacological effects and their secondary outcomes. The safe dose of CMAB009 for crab-eating macaques was 24 mg/kg. The target organs for toxic effects were the skin and other squamous epithelial tissues, indicating that during clinical trials, close observation of the skin, upper digestive tract, upper respiratory tract, and liver and kidney functions of the subjects should be made, and secondary infections due to local lesions should be prevented.

③Immunotoxicity and/or immunogenicity: The cross-reactivity of CMAB009 with normal human tissues showed that a large number of positive cells were seen only in human skin tissues (epidermis, sweat gland ducts), while the antigens in other organs were negative. After 13 weeks of weekly intravenous infusion (v.d.) in crab-eating macaques, some monkeys in the low-dose group had anti-anti-EGFR rcmAb antibodies in their serum. Analysis by cell proliferation inhibition neutralization test showed that the antibodies in the serum were non-neutralizing. Using immunohistochemistry to detect the cross-reactivity of CMAB009 with tissue sections of normal monkey lymphoid organs, no positive staining cells were found in all tested tissues (including lymph nodes, thymus, and spleen); moreover, the antibody did not cross-react with other important organs of the animal (heart, liver, lungs, kidneys, gastrointestinal tract), but had a significant cross-reactivity with the animal's skin epidermal cells.

④Special safety tests such as hemolysis and local irritation: The hemolysis test results showed that the solution in the test tube did not cause hemolysis or aggregation within 3 hours, meeting the requirements of the hemolysis test. In crab-eating macaques, focal inflammatory cell infiltration in the venous wall and patchy congestion and edema in the subcutaneous tissue adjacent to the blood vessels at the site of intravenous injection indicated that this product had certain irritancy to the blood vessels of crab-eating macaques via i.v. administration.

## 3.2 Phase I Clinical Study

**Study Design:** From March to December 2008, the Cancer Hospital of the Chinese Academy of Medical Sciences undertook a Phase I clinical study of CMAB009, with the aim of evaluating the drug's tolerability and pharmacokinetics in humans. The target population for the Phase I clinical trial included subjects with advanced tumors such as colorectal cancer, squamous cell carcinoma of the head and neck, gastric cancer, non-small cell lung cancer, and pancreatic cancer, who had failed conventional chemotherapy. The trial included both single-dose and multiple-dose experiments, with subjects who completed the single-dose trial potentially entering the multiple-dose trial after a washout period if they met the inclusion criteria. The single-dose trial was divided into low, medium, and high dose groups, with doses of 100mg/m^2^, 250mg/m^2^, and 400mg/m^2^, respectively, enrolling 3, 6, and 6 subjects. The multiple-dose group was divided into Groups A and B: the initial doses were 250mg/m^2^ and 400mg/m^2^, respectively, both followed by a 3-week maintenance dose of 250mg/m^2^, with 7 and 8 subjects enrolled in each group. Subjects who completed the multiple-dose trial and did not show disease progression were offered compassionate use of the drug at a dosage of 250mg/m^2^/week until disease progression (PD).

**Enrollment:** A total of 18 subjects were enrolled in the Phase I clinical study, with 15 receiving multiple doses (3 subjects only completed the single-dose trial due to disease progression), and 8 received compassionate use of the drug [3 with partial response (PR), 5 with stable disease (SD)]. The enrolled subjects were colorectal cancer subjects (n=10), lung cancer subjects (n=7), and gastric cancer subjects (n=1) who had failed 3 to 5 prior chemotherapy regimens.

**Safety Results:** The safety results of the Phase I clinical study indicated that CMAB009 was well-tolerated in humans, with mild adverse reactions, primarily infusion reactions (Grade I–II), such as fever, chills, and skin toxicity-related rash, paronychia (Grade I–II). Laboratory tests (hematologic, hepatic, renal, and cardiac function) showed no clinically significant changes except for one case with increased transaminases.

**Pharmacokinetic Results:** The concentration of CMAB009 in serum was determined using a flow cytometry competition method. Pharmacokinetic parameters were calculated using the 3P97 practical pharmacokinetic statistical program.

After intravenous administration of CMAB009 at doses of 100mg/m^2^, 250mg/m^2^, and 400mg/m^2^, the mean C_max_ values were 48.15±10.43, 127.98±36.32, and 208.77±30.21μg/ml, respectively, the mean t_1/2_ values were 47.47±6.61, 78.42±6.23, and 83.58±15.86 hours, respectively, the mean CL values were 34.81±7.55, 17.63±6.18, and 14.68±2.93 ml/h.m^2^, respectively, the mean AUC_0-t_ values were 2939.01±569.47, 15525.30±4797.02, and 26615.95±4827.53 μg.h/ml, respectively, and the mean AUC_0-∞_ values were 2955.60±571.57, 15564.81±4812.12, and 28177.37±5647.10μg.h/ml. Statistical analysis showed that after a single intravenous dose of CMAB009, the C_max_ increased proportionally with the dose within the range of 100 mg/m^2^ to 400 mg/m^2^; the increase in AUC_0-t_ and AUC_0-∞_ was greater than the dose increase ratio. The clearance rate dropped by half from 100 mg/m^2^ to 250 mg/m^2^, and remained almost unchanged from 250 mg/m^2^ to 400mg/m^2^, consistent with the conclusion reported abroad that the clearance of cetuximab reaches saturation within the dose range of 200 mg/m^2^ to 400mg/m^2^ ^[24]^.

In the multiple-dose trial, subjects received initial doses of 250 mg/m^2^ (Group A) and 400 mg/m^2^ (Group B), followed by a 3-week maintenance dose of 250 mg/m^2^. The mean t_1/2τ4_ values for Groups A and B were 106.6±33.5 and 108.±15.5 hours, respectively, the mean AUC_0-tτ4_ values were 14021.9±3860.0 and 16970.9±2779.8 μg.h/ml, respectively, and the mean AUC_0-tτ1_ values were 7355.3±1795.6 and 12407.2±3910.7 μg.h/ml. The average peak concentrations for Groups A and B were 118.76±26.49 and 155.54±25.28 μg/ml, respectively, and the average trough concentrations were 37.54±18.31 and 52.72±14.57 μg/ml, respectively. The results showed that an initial dose of 400 mg/m^2^ with a maintenance dose of 250 mg/m^2^ was more likely to reach a steady state, thus we recommend a dosage of an initial dose of 400 mg/m^2^ with a maintenance dose of 250 mg/m^2^ for the upcoming Phase II/III clinical trials until disease progression (PD) or intolerance.

**Efficacy Assessment:** A preliminary assessment of tumor efficacy was conducted for the subjects in the Phase I trial ^[25]^. Among the 18 enrolled subjects, there were 0 cases of complete response (CR), 2 cases of partial response (PR), and the objective response rate (CR+PR) was 11.1%, which is similar to similar products abroad.

In summary, CMAB009 has good safety, mild adverse reactions, and has achieved encouraging efficacy results in a small sample trial. The pharmacokinetic results confirm that the domestic anti-EGFR monoclonal antibody is essentially consistent with similar foreign products, providing a favorable predictive basis for the confirmation of safety and efficacy in the upcoming large-sample Phase II/III clinical trials.

## 3.3 Phase II/III Clinical Study

**Study Design:** From May 2009 to December 2012, this study was commissioned by the Cancer Hospital of the Chinese Academy of Medical Sciences as the leading unit to conduct a randomized, open-label, parallel-controlled, multicenter Phase II/III clinical study. The purpose was to further investigate the efficacy and safety of CMAB009 in combination with irinotecan chemotherapy for synchronous or sequential treatment of KRAS wild-type, metastatic colorectal cancer that had failed treatment with oxaliplatin and fluorouracil. The dosing regimen for CMAB009 was an initial dose of 400 mg/m^2^, administered intravenously over 120 minutes or more; a maintenance dose of 250 mg/m^2^, administered intravenously over 60 minutes or more, once a week; the recommended dose of irinotecan was 180 mg/m^2^, administered intravenously over 90 minutes, once every 2 weeks, with a 2-week treatment cycle. Tumor assessments were conducted every 6 weeks. After reaching PD or other withdrawal criteria, subjects entered the follow-up period with telephone follow-ups for survival status every 4 weeks.

**Enrollment:** The study planned to enroll 495 subjects, with a 2:1 ratio between the experimental group and the control group, i.e., 330 in the experimental group and 165 in the control group. Actual enrollment: 512 subjects, with 342 in the experimental group and 170 in the control group.

**Efficacy Evaluation:** The objective response rate (ORR) in subjects with KRAS gene-positive, metastatic colorectal cancer treated with the combination of CMAB009 and irinotecan was significantly improved compared to irinotecan alone, with 112/337 (33.2%) in the "CMAB009+irinotecan" group and 21/164 (12.8%) in the irinotecan group, representing an increase of nearly 20%. Additionally, subjects who experienced tumor progression after treatment with irinotecan and then received CMAB009 monotherapy also showed responses, with an ORR of 13.9%. This indicates that even as a third-line treatment, CMAB009 can still provide a certain degree of therapeutic effect. In terms of secondary efficacy endpoints, the median progression-free survival (PFS) time in the "CMAB009+irinotecan" group was 169 days (95% CI, 153 to 183), while in the irinotecan group, it was 95 days (95% CI, 85 to 116), with P<0.0001, indicating a significant extension of progression-free survival. Furthermore, subjects who experienced tumor progression after treatment with irinotecan and then received CMAB009 monotherapy also showed an extension of PFS by 84 days. In other secondary endpoints, the disease control rate (DCR), clinical benefit rate (CBR), and duration of response (DOR) in the "CMAB009+irinotecan" group were all superior to the irinotecan alone group. The PPS analysis results were consistent with the FAS analysis results.

**Safety Evaluation:** Similar to previous studies based on irinotecan treatment, the proportion of gastrointestinal disorders was still the main part, with incidence rates of 65.7% and 66.7% in the "CMAB009+irinotecan" group and the irinotecan group, respectively. Diarrhea was the primary issue, with other conditions such as nausea, constipation, and abdominal pain also occurring at higher rates. The combination with the antibody moderately increased the risk of leukopenia (50% vs 39.4%), neutropenia (30.5% vs 19.4%), and infection (20.1% vs 12.1%). The infusion reactions due to the combination with the antibody were slightly higher (42% vs 33.9%). The adverse events caused by CMAB009 were most notably skin tissue-related toxicities. The overall incidence rate was higher in the "CMAB009+irinotecan" group than in the irinotecan group, with rates of 71.6% and 15.3%, respectively, and rash being the most common (66.9% vs 5.5%). The incidence of rash with CMAB009 monotherapy was 49.6%. This is consistent with the skin-related adverse events reported with cetuximab.

In conclusion, the recombinant human-mouse chimeric monoclonal antibody injection (CMAB009) in combination with irinotecan for the treatment of KRAS wild-type, metastatic colorectal cancer that had failed treatment with oxaliplatin and fluorouracil, has definite efficacy and good safety. It also has certain efficacy and good safety as a third-line treatment for patients who progressed on irinotecan. Therefore, CMAB009 has significant efficacy and good safety. However, due to the change in the manufacturing site, the sponsor decided to conduct a new Phase III clinical trial to further verify the safety and efficacy of CMAB009 after the change in the manufacturing site, in order to provide sufficient basis for the final drug registration application to be approved.

## 3.4 Known and Potential Risks and Benefits to the Study Population

The completed Phase II/III clinical study of the recombinant anti-EGFR human-mouse chimeric monoclonal antibody injection (CMAB009) has shown that interstitial lung disease, infusion reactions, and hypersensitivity reactions are significant known risks associated with CMAB009; spontaneous pneumothorax is considered a significant potential risk. Serious adverse events (SAEs), treatment discontinuation, withdrawal, or death due to the aforementioned risks have not occurred in large numbers, and patient symptoms have been alleviated or controlled to varying degrees after the investigators managed the conditions according to the relevant requirements of the protocol.

Furthermore, clinical data from marketed recombinant anti-EGFR human-mouse chimeric monoclonal antibodies indicate that these antibodies are well-tolerated, with only a small portion of subjects discontinuing treatment due to adverse reactions, and most adverse reactions are controllable or relieved after management.

Overall, under the usage conditions specified in the clinical trial protocol and the designed dosages, the benefit-risk ratio of CMAB009 is favorable.

# 4 Study Objectives

To evaluate the efficacy and safety of the combination of recombinant anti-EGFR human-mouse chimeric monoclonal antibody injection (CMAB009) with the FOLFIRI (irinotecan, 5-fluorouracil, leucovorin) chemotherapy regimen as a first-line treatment for patients with RAS/BRAF wild-type, metastatic colorectal cancer, compared to the FOLFIRI regimen alone.

# 5 Study Design

## 5.1 Overall Study Design and Plan

This open-label, randomized, controlled, multicenter Phase III clinical trial aims to evaluate the combination therapy of "anti-EGFR monoclonal antibody + FOLFIRI" (Group A) compared to FOLFIRI monotherapy (Group B) as first-line treatment for patients with RAS/BRAF wild-type, metastatic colorectal cancer. The primary endpoint is progression-free survival (PFS), assessed according to RECIST version 1.1. Secondary efficacy endpoints include objective response rate (ORR), one-year survival rate, two-year survival rate, overall survival time (OS), disease control rate (DCR), clinical benefit rate (CBR), duration of response (DOR), time to response (TTR), quality of life assessed by the EORTC-QLQ-C30 questionnaire, and the rate of curative surgical resection of liver metastases. Standard safety endpoints include adverse events, vital signs, physical examination, laboratory test indicators, and drug exposure. Other endpoints include the immunogenicity of the anti-EGFR monoclonal antibody and pharmacokinetics.

This study will be conducted at approximately 60 centers, and eligible subjects will be randomly assigned in a 1:1 ratio to the following treatment groups. The overall study design is illustrated in the figure below:


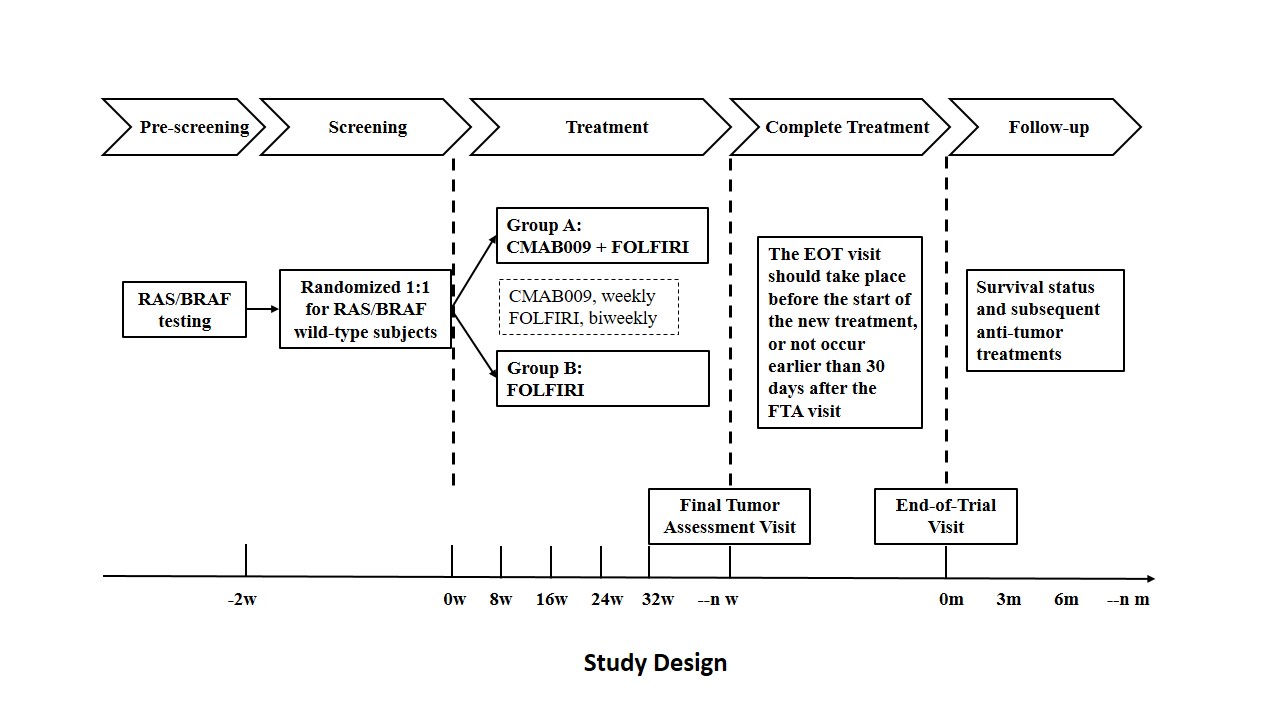


### 5.1.1 Pre-screening

Pre-screening will be arranged after the signing of the written informed consent form. The primary purpose of this period is to assess the RAS/BRAF status of the tumor tissue.

### 5.1.2 Screening Period

Only subjects with RAS/BRAF wild-type status will be eligible to enter the screening period. The screening period extends from the date of obtaining the genetic testing report at each center until random assignment, not exceeding 14 days. The objective is to complete all baseline assessments (including tumor measurements) and ensure that subjects meet the inclusion criteria and do not meet the exclusion criteria before random assignment.

### 5.1.3 Treatment Period

Within 72 hours after randomization, subjects in Groups A and B will commence study treatment until disease progression is confirmed by computed tomography (CT) or magnetic resonance imaging (MRI), the individual subject experiences intolerable AEs, or the subject withdraws informed consent.

A treatment cycle in this study is 14 days, determined by the chemotherapy interval:

**Group A:** A treatment cycle includes anti-EGFR monoclonal antibody and irinotecan/leucovorin (LV) on Day 1 and 5-fluorouracil (5-FU) on Days 1 and 2, anti-EGFR monoclonal antibody on Day 8, followed by a follow-up to Day 14 of the cycle;

**Group B:** A treatment cycle includes irinotecan/leucovorin (LV) on Day 1 and 5-fluorouracil (5-FU) on Days 1 and 2, followed by a follow-up to Day 14 of the cycle;

Anti-EGFR monoclonal antibody or chemotherapy may be delayed due to toxicity. If the treatment is delayed due to toxicity related to anti-EGFR monoclonal antibody therapy, the 14-day cycle of chemotherapy remains unchanged. A maximum of four consecutive weekly anti-EGFR monoclonal antibody infusions (not exceeding 28 days) can be omitted. If the delay exceeds the aforementioned time limit, subjects should withdraw from anti-EGFR monoclonal antibody therapy but may continue to receive chemotherapy until disease progression.

If chemotherapy is delayed due to toxicity from any chemotherapeutic drug (5-FU/LV or irinotecan), other chemotherapy should continue. If a subject experiences toxicity from both chemotherapeutic agents, the overall chemotherapy should be postponed. However, in any of the above cases, the 7-day per week infusion of anti-EGFR monoclonal antibody should be maintained. A maximum of two consecutive cycles of chemotherapy (5-FU/LV or irinotecan) administration (not exceeding 28 days) can be omitted. The aforementioned treatment delays must not exceed 28 days during the study period; otherwise, subjects must withdraw from chemotherapy but may continue to receive anti-EGFR monoclonal antibody therapy until disease progression.

If permanent discontinuation of both anti-EGFR monoclonal antibody and chemotherapy occurs due to toxicities from both treatments, subjects are still required to participate in the study (i.e., continue to undergo efficacy assessments every 8 weeks) until disease progression.

Once further tumor assessment visits cannot be conducted due to the subject's withdrawal of informed consent or other reasons, investigators should make every effort to obtain the final tumor measurement data; if possible, a final tumor assessment (FTA) should be conducted on the subject, and other required information should be collected.

No other anti-neoplastic treatment should be administered to subjects before disease progression. After disease progression, all study medication treatments should be discontinued (if still being administered).

Treatment for Groups A and B will continue until disease progression is confirmed by CT or MRI, or the subject experiences intolerable AEs or withdraws informed consent. If treatment with the anti-EGFR monoclonal antibody is discontinued before PD (see above), subjects should continue chemotherapy until PD and undergo assessment visits every 8 weeks, with efficacy evaluated through CT or MRI scans. If treatment with a component of chemotherapy is discontinued before PD (see above), subjects may continue the other component of chemotherapy until PD and undergo assessment visits every 8 weeks, with efficacy evaluated through CT or MRI scans.

No other anti-neoplastic treatment should be received before confirmed PD. Once any other anti-neoplastic treatment is received before PD, a final tumor assessment visit must be conducted.

### 5.1.4 Assessment Visits Every 8 Weeks

Each treatment cycle involves multiple therapeutic or study assessment-related visits. Additionally, regardless of whether the anti-EGFR monoclonal antibody or chemotherapy is delayed, subjects should undergo an evaluation visit every 8 weeks post-randomization. These assessment visits include tumor assessments based on CT or MRI, completion of the EORTC-QLQ-C30 questionnaire, evaluation of AEs, ECOG performance status, concomitant medications, vital signs, physical examination, and laboratory tests. Details of the assessments conducted at each visit are provided in Section 7.1.4 and the study flowchart.

### 5.1.5 Final Tumor Assessment (FTA) Visit

Once disease progression is identified in a subject (e.g., during a scheduled treatment assessment visit), a final tumor assessment (FTA) should be conducted. If progression is detected outside of a planned visit, the FTA should be performed as soon as possible. At this time, information on disease progression will be collected to facilitate the study of the primary endpoint. If disease progression is determined during a scheduled every 8-week assessment visit, a dedicated FTA visit is not required; the last visit at the time of disease progression is considered the FTA visit.

### 5.1.6 End of Trial (EOT) Visit

The EOT visit should not occur earlier than 30 days after the FTA visit. However, if another anti-neoplastic treatment has commenced, the EOT visit must take place before the start of the new treatment.

### 5.1.7 Long-term Follow-up Period

During the follow-up period, if a subject discontinues treatment due to reasons other than disease progression or withdrawal of informed consent, every effort should be made to collect tumor imaging data for tumor assessment until disease progression or the initiation of another anti-neoplastic treatment (whichever occurs first).

After the EOT visit, all subjects will undergo follow-up assessments every 3 months (90±3 days) to collect information on vital status and subsequent anti-neoplastic treatments until death or loss to follow-up. For any skin reactions that persist at the time of the EOT visit, further monitoring should be conducted to determine the final outcome and the date of resolution of the aforementioned AEs (up to 6 months). Details of subject AE monitoring are provided in Section 7.5.8.

### 5.1.8 Scientific Rationale for Study Design

**Scientific Basis for the Dosing Regimen:** The pharmacokinetic results from the Phase I clinical study of CMAB009 indicated that, during multiple dosing, a weekly administration of 400 mg/m² as the initial dose and 250 mg/m² as the maintenance dose is more likely to achieve steady-state compared to a fixed dose of 250 mg/m². This regimen is consistent with the administration of Erbitux^®^. In the Phase II/III clinical trials, the use of this regimen in combination with irinotecan chemotherapy for patients with metastatic colorectal cancer who had failed previous treatments demonstrated significant efficacy and good tolerability. The NCCN guidelines recommend both weekly and biweekly regimens for cetuximab in combination with chemotherapy, with the biweekly regimen of 500 mg/m² every two weeks showing better compatibility with FOLFIRI or FOLFOX chemotherapy in clinical practice. Although the amino acid sequence of CMAB009 is identical to that of Erbitux^®^, the safety of the 500 mg/m² every two weeks regimen has not been validated in prior studies. Therefore, in the Phase III clinical trial, CMAB009 will continue to use the initial dose of 400 mg/m² and the maintenance dose of 250 mg/m², administered weekly.

**Rationale for Control Group Selection:** In the NCCN and Chinese Society of Clinical Oncology (CSCO) guidelines (v.2020), both FOLFIRI chemotherapy in combination with cetuximab and FOLFIRI monotherapy are recommended as first-line treatments for patients with unresectable metastatic colorectal cancer. At the time this study was initiated, the NMPA had not yet approved the use of Erbitux^®^ in combination with FOLFIRI chemotherapy for first-line treatment of metastatic colorectal cancer. Therefore, the control group was selected as FOLFIRI chemotherapy, which is also a standard treatment, designed as an efficacy study.

**Rationale for Open-label Design and Measures to Control Bias:** In the Phase II/III clinical trial, the most notable adverse reaction associated with CMAB009 was skin toxicity, with the incidence of rash in the experimental group being 4-5 times higher than that in the control group (71.6% vs 15.3%). Skin toxicity is the most commonly reported adverse reaction in the safety information for Erbitux^®^, and the severity of rash has been positively correlated with treatment efficacy. Skin toxicity may even serve as a clinical surrogate marker for the efficacy of cetuximab, although the exact mechanism of this relationship remains unclear. Given the differences in skin toxicity between treatment groups, this study is designed as an open-label trial. The primary endpoint, progression-free survival (PFS), and the secondary endpoint, objective response rate (ORR), will be assessed through independent imaging with blinded review to control for potential bias in efficacy evaluation.

## 5.2 Selection of Study Population

### 5.2.1 Diagnostic Criteria

The diagnostic criteria for metastatic colorectal cancer are defined by the 2010 revision of the TNM staging system jointly established by the International Union Against Cancer (UICC) and the American Joint Committee on Cancer (AJCC) corresponding to Stage IV (see Appendix 1).

### 5.2.2 Definition of First-line Treatment Population

Subjects who have the first appearance of metastatic lesions (unresectable for cure), have not received chemotherapy, or have previously received adjuvant chemotherapy or neoadjuvant chemotherapy for colorectal cancer (CRC), with a chemotherapy-free interval of ≥12 months from the discovery of disease recurrence or metastasis.

### 5.2.3 Inclusion Criteria

Only subjects who meet all of the following criteria are eligible for enrollment:

1. Age between 18 and 75 years, regardless of gender;
2. Histologically confirmed adenocarcinoma of the colon or rectum;
3. First occurrence of metastatic lesions (unresectable for cure);
4. Tumor tissue with wild-type RAS/BRAF genes;
5. At least one measurable lesion according to RECIST 1.1 criteria confirmed by CT or MRI (outside of a radiation therapy field);
6. ECOG performance status of 0 to 1;
7. Life expectancy of more than 3 months with a residence conducive to follow-up;
8. Subjects of childbearing potential must undergo effective medical contraception (for both male and female subjects until 90 days after the last study drug administration);
9. Recovery from any toxicities related to previous treatments before study enrollment;
10. Willingness to sign an informed consent form.

### 5.2.4 Exclusion Criteria

Subjects who meet any of the following criteria are not eligible to participate in the trial:

1. Radiation therapy or surgery (except for diagnostic biopsy) within 30 days before the start of treatment;
2. Organ function levels as follows:

Hematopoietic system: White blood cells (WBC) <3.0×10^9^/L; Neutrophils (ANC) <1.5×10^9^/L; Platelets (PLT) <100×10^9^/L; Hemoglobin (Hb) <90g/L;

Liver: Total bilirubin (TBIL) >1.5 times the upper limit of normal reference range; Alanine aminotransferase and aspartate aminotransferase (ALT and AST) >2.5 times the upper limit of normal reference range (for those without liver metastasis) or >5 times the upper limit of normal reference range (for those with liver metastasis);

Kidneys: Serum creatinine (Cr) >1.5 times the upper limit of normal or creatinine clearance <50mL/min;

1. Previous adjuvant or neoadjuvant chemotherapy for CRC with a chemotherapy-free interval of less than 12 months from the discovery of disease recurrence or metastasis;
2. Previous exposure to anti-EGFR monoclonal antibodies, EGFR tyrosine kinase inhibitors, or other EGFR-targeted therapies (e.g., cetuximab, nimotuzumab, panitumumab, etc.);
3. Known hypersensitivity or allergic reactions to any component of the study treatment;
4. History of organ transplantation, autologous/allogeneic stem cell transplantation;
5. Other concurrent antineoplastic treatments (forbidden concomitant treatments detailed in Section 6.5.1);
6. Symptomatic brain and/or leptomeningeal metastases;
7. History of other malignant tumors within the last 5 years, except for carcinoma in situ of the cervix, basal cell carcinoma of the skin, or squamous cell carcinoma;
8. Participation in other clinical trials of antineoplastic drugs within 30 days before enrollment;
9. Receiving chronic systemic immunotherapy or hormone therapy other than physiological replacement therapy;
10. Any unstable systemic disease:

Including active infection, uncontrolled hypertension, unstable angina, onset of angina within the last 3 months, congestive heart failure (New York Heart Association [NYHA] Class ≥ II), myocardial infarction within 6 months before enrollment, severe arrhythmias requiring medication, liver, kidney, or metabolic diseases;

1. Acute or subacute intestinal obstruction, or a history of inflammatory bowel disease;
2. Subjects with severe bone marrow failure;
3. Any disease, metabolic disorder, or medical examination or laboratory findings that suggest the patient has contraindications to the study drug or is at high risk for treatment complications;
4. Known or self-reported human immunodeficiency virus (HIV) infection;
5. Hepatitis B virus DNA positive (copy number greater than 10^3^);
6. Pregnancy or lactation;
7. Known alcohol or drug addiction, or other poor health conditions or mental states that may affect protocol compliance and evaluation of trial endpoints, as determined by the investigator, making the subject unsuitable for the study;
8. Lack of legal capacity or limited legal capacity.

## 5.3 Randomization

Only subjects with wild-type RAS/BRAF status in tumor tissue are eligible for randomization. Subjects who withdraw informed consent prior to randomization are not permitted to participate in the randomization process. Subjects who do not meet the inclusion criteria or meet the exclusion criteria after signing the informed consent form are also ineligible for randomization. Once a patient has signed the informed consent form, the reasons for not participating in randomization must be documented in the Case Report Form (CRF).

Upon completion of the screening period assessments, all eligible subjects will be randomly assigned to Group A and Group B in a 1:1 ratio. Randomization will be conducted based on a central randomization system, with the stratification factors being the study site and ECOG performance status (two levels, ECOG performance status of 0 and 1). The designated person in charge at the participating center logs into the randomization system, enters the subject's relevant information, and the system will then display the group assignment for that case. Subsequently, this person will notify the investigator of the group to which the subject has been assigned for the study treatment. The randomization number of the enrolled case must be recorded in the electronic Case Report Form (eCRF), and each subject can only participate in one randomization assignment.

## 5.4 Criteria for Subject Withdrawal

### 5.4.1 Withdrawal from the Study

Subjects may freely withdraw from the study at any time without any reason. Once informed consent is withdrawn, subjects must be withdrawn from the study.

If a subject is unable to participate in regular study assessments, the investigator must ascertain the reasons and circumstances as completely and accurately as possible.

Should a subject prematurely withdraw from the study, a Follow-up or End-of-Treatment visit should be conducted according to the study schedule, focusing on the most relevant assessments if possible. Under any circumstances, the corresponding sections of the Case Report Form (CRF) must be completed.

Upon withdrawal from the study, the subject's assigned subject identification code remains unchanged.

### 5.4.2 Discontinuation of Study Treatment

#### 5.4.2.1 Discontinuation of All Study Treatments

In the event of any of the following occurrences during this trial, subjects will be discontinued from all treatments but will continue to receive survival follow-up:

1. Lack of compliance by the subject;
2. Intolerability of an adverse event (AE) that the subject and/or investigator deems necessary to discontinue treatment;
3. Use of other concurrent anticancer treatments that the investigator or sponsor believes may affect the outcome assessment;
4. Tumor disease progression in the subject;
5. Occurrence of certain comorbidities, complications, or special physiological changes that the investigator judges to be unsuitable for continued participation in this clinical study;
6. Pregnancy during the treatment period.

#### 5.4.2.2 Discontinuation of CMAB009 Treatment

In the event of any of the following occurrences during this trial, subjects will be discontinued from anti-EGFR monoclonal antibody treatment but will remain in the study to receive chemotherapy:

1. Consecutive interruptions of anti-EGFR monoclonal antibody infusion for more than 28 days;
2. Occurrence of an AE that the subject and/or investigator deems necessary to discontinue anti-EGFR monoclonal antibody treatment;
3. Any Grade 4 or above intolerable toxicity related to anti-EGFR monoclonal antibody;
4. Intolerable infusion reactions of Grade 3 or higher related to anti-EGFR monoclonal antibody (excluding fever);
5. Diagnosis of interstitial lung disease during the study period;

In the event of specific toxicities, the dose of anti-EGFR monoclonal antibody may be adjusted or treatment may be delayed (see 6.1.4).

#### 5.4.2.3 Discontinuation of Chemotherapy Treatment

In the event of any of the following occurrences during this trial, subjects will be discontinued from chemotherapy but will remain in the study to receive anti-EGFR monoclonal antibody treatment:

1. Intolerable toxicity such as Grade 3 nausea or Grade 4 vomiting despite prophylactic medication;
2. Any clear Grade 4 intolerable drug-related toxicity;
3. Grade 1 cardiac toxicity (if related only to 5-FU/LV, discontinue 5-FU/LV treatment);
4. The subject voluntarily withdraws and the investigator deems it appropriate for the subject to discontinue chemotherapy while maintaining monoclonal antibody treatment.

If possible, subjects who discontinue treatment before disease progression should still undergo efficacy assessments every 8 weeks until disease progression.

In the event of specific toxicities, the dose of chemotherapy may be adjusted or treatment may be delayed (see 6.2.3).

## 5.5 Premature Termination of Study Treatment

Under any of the following circumstances, the sponsor may propose to terminate the study prematurely:

1. New information leads to an unfavorable risk-benefit assessment of the study drug, such as evidence of the ineffectiveness of the study treatment;
2. The sponsor deems that continuation is not feasible from either a medical or ethical standpoint;
3. The sponsor discontinues the study drug;
4. There is evidence to suggest that the expected study objectives have been achieved prematurely.

The entire study may be suspended or discontinued at the request of the health regulatory authority.

## 5.6 Definition of Study Completion

The study will be concluded after at least 80% of the randomized subjects have expired or been lost to follow-up. We will still provide the medication to subjects who continue to receive treatment.

# 6 Study Medication

## 6.1 Anti- EGFR Monoclonal Antibody

Drug Name: Recombinant Anti-EGFR Human-Mouse Chimeric Monoclonal Antibody Injection

Code Number: CMAB009

Specification: 100mg/10ml per vial

Production Batch Number: See outer packaging of the pharmaceutical product

Shelf Life: Tentatively established at 3 years

Storage: Store at 2-8°C, protected from light

Manufacturing Company: Taizhou Mabtech Pharmaceutical Co., Ltd.

### 6.1.1 Packaging of Anti-EGFR Monoclonal Antibody

The investigational drug is packaged in vials within a carton. Each small box contains one vial, and each large box contains ten vials. Both small and large box labels display information including the drug name, specification, dosage, storage, production batch number, manufacturing date, expiration date, and manufacturing company, with the notation "For Clinical Trial Use Only."

### 6.1.2 Preparation of Anti-EGFR Monoclonal Antibody Prior to Use

The preparation process must strictly adhere to aseptic techniques. The recombinant anti-EGFR human-mouse chimeric monoclonal antibody injection should be diluted in 0.9% sterile saline solution to a total volume of 500 ml. As the drug does not contain any antimicrobial preservatives or bacteriostatic agents, it must be used immediately upon opening. **If immediate use is not possible, it may be stored at room temperature for up to 8 hours.** The recombinant anti-EGFR human-mouse chimeric monoclonal antibody injection must not be mixed with other drugs or administered through the same intravenous line. If concurrent intravenous administration of other drugs with the recombinant anti-EGFR human-mouse chimeric monoclonal antibody injection is necessary, a second intravenous line must be used.

### 6.1.3 Administration Schedule of Anti-EGFR Monoclonal Antibody

The initial dose of the anti-EGFR monoclonal antibody is 400 mg/m^2^, administered intravenously over at least 120 minutes; the maintenance dose is 250 mg/m^2^, administered intravenously over at least 60 minutes, once weekly. At the end of the infusion, the infusion line must be flushed with saline to ensure complete injection of any residual drug. The drug is continued until disease progression (PD) or until other trial withdrawal criteria are met.

**Prior to the first administration of the anti-EGFR monoclonal antibody, subjects must receive pretreatment with antihistamines and corticosteroids to reduce the risk of infusion reactions. It is also recommended to use antihistamines and corticosteroids as prophylactic medication before subsequent maintenance dose infusions of the anti-EGFR antibody. All premedication should be recorded on the CRF.**

The total infusion time for the anti-EGFR monoclonal antibody must not exceed 240 minutes.

The dose of the anti-EGFR monoclonal antibody is calculated based on the subject's body surface area (BSA) and adjusted accordingly for each treatment cycle. BSA should be calculated using the Mosteller formula ^[26]^: BSA (m^2^) =$\sqrt{height (cm) \times weight (Kg) / 3600}$. There is no upper limit for the use of the anti-EGFR monoclonal antibody for BSA values >2. The actual dose of the anti-EGFR monoclonal antibody administered each time (with an allowable error of **±10 mg**), date, and start and end times must be accurately recorded.

The infusion of the anti-EGFR monoclonal antibody must be completed 1 hour before the administration of FOLFIRI.

### 6.1.4 Dose Adjustment of Anti-EGFR Monoclonal Antibody

- Non-hematological toxicity: All non-hematological toxicities are graded according to the National Cancer Institute (NCI) Common Terminology Criteria for Adverse Events (CTC AE V4.03), see Appendix 2. Non-hematological toxicities graded ≥ Grade 4 must be discontinued immediately.
- Dose adjustments for infusion reactions and skin toxicity are detailed in sections 6.1.5 ② and ③.
- For subjects undergoing curative surgery for metastatic lesions (see section 6.7), treatment may be delayed for 4-10 weeks.
- Dose reduction of the anti-EGFR monoclonal antibody is permanent. If the administration of the anti-EGFR monoclonal antibody is interrupted for more than 28 consecutive days (except for subjects undergoing curative surgery for metastatic lesions), the subject must be withdrawn from treatment with the anti-EGFR monoclonal antibody but may continue in the study until disease progression. Subjects permanently withdrawn from treatment with the anti-EGFR monoclonal antibody may continue to receive chemotherapy, and if treatment with the anti-EGFR monoclonal antibody is delayed, chemotherapy should not be delayed. Similarly, subjects permanently withdrawn from chemotherapy may continue to receive treatment with the anti-EGFR monoclonal antibody. If treatment is interrupted for more than 28 days, the study treatment should be discontinued. In special circumstances, the investigator may consult with the sponsor to allow subjects to continue treatment with the anti-EGFR monoclonal antibody.

### 6.1.5 Precautions

**①Monitoring during drug administration**

The first use of this drug should be conducted under the guidance of an experienced physician, and the subject's vital signs (temperature, respiration, blood pressure, and heart rate/pulse) as well as facial complexion, sweating, or headache should be closely monitored before, during, and one hour after infusion to detect early signs of infusion reactions.

Some infusion reactions may occur in subsequent treatment phases, and even if no infusion reaction of any grade occurs after the first use, subsequent drug administration should still be carried out under the supervision of a physician.

**②Management of infusion reactions**

Despite prophylaxis with antihistamines, infusion reactions may still occur during drug administration. In the event of an infusion reaction, the investigator should refer to the National Cancer Institute (NCI) Common Terminology Criteria for Adverse Events (CTC AE V4.03) (see Appendix 2) and Table 1 for management, closely monitoring the subject until all symptoms have completely resolved.

**Table 1 Management of infusion reactions**

| **Grade** | **Symptom** | **Adjustment to CMAB009** | **Therapeutic Interventions** |
| --- | --- | --- | --- |
| Grade I/II | 1. Pyrexia ≤ 40.0°C 2. Chills 3. Chest tightness 4. Rash 5. Nausea 6. Dyspnea | 1. Discontinuation of CMAB009 may not be necessary. 2. The infusion rate may be reduced to 50%, and it is recommended to maintain this adjusted rate throughout the subsequent infusion process. | In the subsequent treatment process, strict monitoring is mandatory |
| Grade III/IV | 1. Pyrexia ＞ 40.0°C 2. Rapidly developing symptoms of airway obstruction (Bronchospasm, stridor, hoarseness) 3. Urticaria 4. Hypotension | Discontinue CMAB009 therapy immediately and permanently | Close monitoring and appropriate symptomatic treatment, such as epinephrine, corticosteroids, intravenous antihistamines, and oxygen therapy |

**③Dermatological Toxicity**

Subjects may experience dermatological reactions, primarily manifesting as acneiform rash, often occurring on the face, upper chest, and back, and typically appearing within the first two weeks of treatment. Once dermatological toxicity occurs, it can be graded according to the criteria outlined in Table 2. In the event that a subject exhibits dermatological toxicity of Grade III or higher, the investigator should make corresponding dose adjustments as per the standards listed in Table 3.

During the trial, ample attention should also be given to the condition of skin inflammation and infection. In the case of severe skin infection, in addition to adjusting the dosage of this medication, treatment with topical or oral antibiotics may be selected, although the use of topical corticosteroids is not recommended.

**Table 2 Grading Criteria for Dermatological Toxicity**

| **Grade*** | **Clinical Manifestations** |
| --- | --- |
| Grade I | Maculopapular rash, papules, or erythema without associated symptoms |
| Grade II | Maculopapular rash, papules, or erythema with associated symptoms or that are pruritic;  localized desquamation or affected areas involving less than 50% of the body surface area |
| Grade III | Generalized erythema with symptoms, or maculopapular rash, papules, or vesicular eruptions;  or desquamation affecting more than 50% of the body surface area |

*Assessed and categorized based on the CTCAE V2.0 standards

**Table 3 Standards for Managing Dermatological Toxicity Grade III and Above**

| **Occurrence of Dermatotoxicity** | **Drug Administration** | **Outcome** | **Dosage Adjustment** |
| --- | --- | --- | --- |
| 1^st^ time | Delay | Return to ≤ Grade II Toxicity Reaction | Maintenance of a Dose of 250mg/m^2^/week |
|  |  | No Improvement | Discontinuation |
| 2^nd^ time | Delay | Return to ≤ Grade II Toxicity Reaction | Reduction to a Dose of 200mg/m^2^/week |
|  |  | No Improvement | Discontinuation |
| 3^rd^ time | Delay | Return to ≤ Grade II Toxicity Reaction | Reduction to a Dose of 150mg/m^2^/week |
|  |  | No Improvement | Discontinuation |
| 4^th^ time | Discontinuation |  |  |

**④Respiratory Abnormalities:** There have been individual case reports of interstitial lung disease (ILD) with an unclear relationship to anti-EGFR monoclonal antibodies. In the event of a diagnosis of ILD, treatment with the anti-EGFR monoclonal antibody should be discontinued, and subjects should receive appropriate therapy.

**⑤Electrolyte Imbalances:** Progressive hypomagnesemia, potentially leading to severe magnesium deficiency, may occur during the course of treatment. When it arises, appropriate treatment should be administered. Treatment with the anti-EGFR monoclonal antibody may continue unless the investigator deems it poses a safety risk. Hypomagnesemia is reversible upon discontinuation of the anti-EGFR monoclonal antibody treatment.

**⑥Other:** The anti-EGFR monoclonal antibody must not be mixed with any other intravenous medications. For Group A subjects, the FOLFIRI chemotherapy regimen must be administered at least one hour after the completion of the anti-EGFR monoclonal antibody infusion, using a separate infusion line.

## 6.2 FOLFIRI Chemotherapy Regimen

### 6.2.1 Composition of the FOLFIRI Chemotherapy Regimen

①Irinotecan

②Leucovorin

③5-Fluorouracil

The FOLFIRI chemotherapy drugs are procured by the sponsor from marketed products, with dosage forms, packaging, and labeling based on actual purchases. Detailed information of all FOLFIRI chemotherapy drugs used by all subjects will be recorded (such as trade name, manufacturer, batch number, etc.).

### 6.2.2 FOLFIRI Administration Schedule

Irinotecan 180 mg/m^2^, administered intravenously over 30 to 90 minutes, on Day 1; Leucovorin (LV) 400 mg/m^2^, should be administered immediately after the infusion of Irinotecan, with a maximum interval not exceeding 30 minutes, with the same infusion time, on Day 1; 5-Fluorouracil (5-FU), 400 mg/m^2^, administered by intravenous bolus or rapid intravenous infusion, on Day 1; followed by a continuous intravenous infusion of 1,200 mg/m^2^/day × 2 days (total 2,400 mg/m^2^, recommended infusion over 46 to 48 hours), to be repeated every 2 weeks. Each 14-day period constitutes a cycle.

Doses are calculated based on the subject's body surface area (BSA) and adjusted accordingly for each treatment cycle. The actual dose of each infusion (with an allowable error of **±10 mg**), date, and start and end times must be accurately recorded.

### 6.2.3 Dose Adjustments for the FOLFIRI Regimen

A complete blood count and hepatorenal function tests should be performed within three days prior to each chemotherapy session. In the event of toxicity reactions of the grades indicated below, drug doses must be adjusted in accordance with Table 4. Following the occurrence of toxicity reactions as described in Tables 5 or 6, dosing adjustments should be made according to the guidelines provided within Tables 5 or 6, respectively. Grading of adverse reactions is based on the Common Terminology Criteria for Adverse Events (CTCAE 4.03) established by the National Cancer Institute (NCI), as detailed in Appendix 2. Chemotherapy should not be postponed due to the toxicity associated with the anti-EGFR monoclonal antibody.

Group A subjects are to receive the FOLFIRI regimen on Day 1 of each treatment cycle. Should a delay occur and the subject has not recovered more than three days beyond the scheduled treatment time, the regimen should be postponed to the next administration of CMAB009 in combination, with a corresponding adjustment to the subsequent FOLFIRI infusion window.

**Table 4 FOLFIRI Dose Adjustment**

|  | - **Initial Dose (mg/m^2^)** | - **Dose Reduction-1** | **Dose Reduction-2** |
| --- | --- | --- | --- |
| Irinotecan | 180 | 150 | 120 |
| Leucovorin | 400 | 320 | 240 |
| 5-Fluorouracil Bolus Injection | 400 | 320 | 240 |
| 5-Fluorouracil Infusion | 2400 | 2000 | 1500 |

**Table 5 Chemotherapy Dose Adjustment Criteria for Hematological Toxicity**

| **Hematological Toxicity** | **Grade 2** | **Grade 3-4** |
| --- | --- | --- |
| Neutropenia | - Not Applicable | - No Dose Reduction Unless Grade 4 Neutropenia > 7 Days |
| Febrile Neutropenia and Neutropenic Sepsis | Not Applicable | - 1^st^ time: Irinotecan Reduced by One Dose Level*; - 2^nd^ time: 5-FU Reduced by One Dose Level; - 3^rd^ time: Discontinue Chemotherapy; |
| Thrombocytopenia | Full Dose | 1^st^ time: 5-FU (Bolus Injection and Infusion) Reduced by One Dose Level;  2^nd^ time: Irinotecan Reduced by One Dose Level;  3^rd^ time: Discontinue Chemotherapy; |

*Prior to confirming a reduction in chemotherapy dose after the first occurrence of the event, initiate G-CSF treatment

**Table 6 Standards for Chemotherapy Dose Adjustment for Non-hematological Toxicity**

| **Non-hematological Toxicity** | **Grade 2** | **Grade 3** | **Grade 4** |
| --- | --- | --- | --- |
| Diarrhea | Full Dose | 1^st^ time: Irinotecan Reduced by One Dose Level;  2^nd^ time: 5-FU (Bolus Injection and Infusion) Reduced by One Dose Level;  3^rd^ time: Discontinue Chemotherapy; | |
| Stomatitis | Full Dose | 5-FU (Bolus Injection and Infusion) Reduced by One Dose Level | 5-FU (Bolus Injection and Infusion) Reduced by Two Dose Level |
| Palmar-Plantar Erythrodysesthesia Syndrome | Full Dose | 5-FU (Bolus Injection and Infusion) Reduced by One Dose Level | Not Applicable |
| Elevated Bilirubin | Postpone the Next Infusion Until Recovery to ≤ Grade 1 (1.5xULN)  1^st^ time: Full Dose;  2^nd^ time: Irinotecan Reduced by One Dose Level;  3^rd^ time: Irinotecan Reduced by Two Dose Level;  4^th^ time: Permanently Discontinue Irinotecan  Postpone for 4 Weeks if Persistent Grade 2 (＞1.5xULN and ≤3xULN)  1^st^ time: Irinotecan Reduced by Two Dose Level;  2^nd^ time: Permanently Discontinue Irinotecan | Postpone the Next Infusion Until Recovery to ≤ Grade 2 (3xULN)  Postpone for 4 Weeks if Persistent Grade 3 (＞3xULN and ≤10xULN), Permanently Discontinue Irinotecan | Permanently Discontinue FOLFIRI |
| Elevated Transaminases | Full Dose | Postpone the Next Infusion Until Recovery to ≤ Grade 2  Postpone for 4 Weeks if Persistent Grade 3, Permanently Discontinue Irinotecan | Permanently Discontinue FOLFIRI |
| Elevated Alkaline Phosphatase | Full Dose | Postpone the Next Infusion Until Recovery to ≤ Grade 2  Postpone for 4 Weeks if Persistent Grade 3, Permanently Discontinue Irinotecan | Permanently Discontinue FOLFIRI |
| Hypersensitivity Reactio | If Caused by Irinotecan, Discontinue Irinotecan | | |

Dose adjustments are permanent, with the administered dose always being modified based on the dose from the previous cycle. Should more severe toxicity occur, or should the subject fail to meet the criteria for chemotherapy during a maximum treatment delay period of two cycles (28 days), the subject will be withdrawn from chemotherapy.

If a subject is unable to receive chemotherapy due to toxicity, the administration may be postponed by one week. If, after a one-week delay, the subject still cannot receive chemotherapy, the administration may be further postponed by an additional week. Once the treatment delay exceeds two cycles (28 days), the subject will discontinue chemotherapy. Even with treatment delays, tumor assessments should proceed as originally scheduled.

For both Group A and Group B subjects, the following criteria must be met before initiating the next cycle of chemotherapy with the FOLFIRI regimen on Day 1 of each treatment cycle:

- Neutrophil count ≥ 1.5 × 10^9^/L;
- Platelet count ≥ 75 × 10^9^/L;
- Chemotherapy-related toxicities such as oral mucositis or diarrhea have recovered to ≤ Grade 1;
- Other chemotherapy-related toxicities have recovered to ≤ Grade 2.

### 6.2.4 Precautions

**①Prophylactic Antiemetics:** Irinotecan is commonly associated with the induction of nausea and vomiting. Prophylactic use of antiemetic drugs should be administered prior to each treatment. Examples include dexamethasone and 5-hydroxytryptamine 3 (5-HT3) receptor antagonists (palonosetron, dolasetron, granisetron, ondansetron, tropisetron, etc.).

**②Monitoring During Medication Use:** A complete blood count and hepatorenal function tests should be conducted before treatment and prior to each cycle of chemotherapy. Subjects presenting with abnormalities in blood counts or hepatorenal function are at significant risk and should be closely monitored, with adjustments in drug dosage or discontinuation of the medication as necessary.

**③Abnormalities in Bilirubin:** When bilirubin levels in subjects exceed 1.0 to 1.5 times the upper limit of normal, the risk of severe neutropenia increases. For these individuals, the frequency of complete blood cell count testing should be increased.

**④Delayed Diarrhea:** There is a risk of delayed diarrhea occurring any time from 24 hours after irinotecan administration until the next cycle of chemotherapy, especially for subjects who have previously received abdominal or pelvic radiotherapy. Diarrhea can be life-threatening, and subjects must be informed to start drinking large amounts of electrolyte-rich fluids and to initiate anti-diarrheal treatment immediately upon the first episode of loose stools: loperamide at a dose of 2 mg every 2 hours, with the initial dose doubled, and continued until 12 hours after the last loose stool, but not to exceed 48 hours of continuous use. Subjects with severe diarrhea or diarrhea lasting more than 24 hours should seek medical attention promptly. After the onset of diarrhea, a lactose-free, alcohol-free, and low-fat diet is advised. It is recommended to eat small, frequent meals.

**⑤Varicella and Herpes Zoster: 5-Fluorouracil should be contraindicated** in subjects with varicella or herpes zoster.

**⑥Hand-Foot Syndrome:** Subjects treated with 5-FU may develop hand-foot syndrome, characterized by erythema and swelling of the palms and soles. Mild hand-foot syndrome is painless, but 10-15% of patients may experience pain accompanied by blisters, ulcers, fissures, and desquamation. Vitamin B6 may be helpful.

**⑦Cardiac Toxicity:** Typical cardiac toxicity symptoms in subjects treated with 5-FU are ischemic pain occurring after intravenous bolus injection or the start of continuous infusion, accompanied by characteristic electrocardiogram (ECG) changes. Asymptomatic ECG changes may also be observed. There have been reports of myocardial infarction. Therefore, if subjects present with such symptoms or have unexplained cardiac events following 5-FU treatment, 5-FU therapy must be discontinued.

**⑧Other:** During medication use, due to the inclusion of 5-FU in the FOLFIRI regimen, alcohol consumption or concomitant use of aspirin-like drugs should be avoided to reduce the possibility of gastrointestinal bleeding.

## 6.3 Medication Adherence

All intravenous infusions must be administered by qualified personnel under observation. Any interruptions in administration, failure to deliver the full dose for any reason, or other such occurrences should be meticulously documented in the medical record and the Case Report Form (CRF).

Non-adherence is defined as a subject missing more than two consecutive infusions of anti-EGFR monoclonal antibody or chemotherapy for non-medical reasons. Non-adherence typically leads to the discontinuation of anti-EGFR monoclonal antibody or chemotherapy, at which point the investigator must consult with the sponsor to determine whether to discontinue the subject's treatment based on the specific circumstances.

## 6.4 Drug Management

1. The investigational drugs, appropriately packaged, are shipped by the sponsor to each clinical trial center. Each center should designate a person responsible for receiving, storing, and managing the drugs according to requirements and maintaining appropriate records (such as temperature records for drug storage, distribution/recovery records, etc.). **Freezing of anti-EGFR monoclonal antibodies is strictly prohibited.**
2. The investigational drugs can only be prescribed by the responsible, authorized investigator.
3. The investigational drugs should be distributed only to the subjects according to the protocol and used only at the trial centers.
4. Investigators must return all unused drugs to the sponsor, who is responsible for the destruction.
5. Drugs past their expiration date must not be used and should be closely monitored by inspectors. Expired drugs should be returned to the sponsor in a timely manner for destruction.

At the end of this trial, it must be ensured that the transportation records of the investigational drugs match the usage and destruction/return records. Any discrepancies should be explained.

## 6.5 Concurrent Treatments

Any medical interventions or changes in concomitant medications required during the study must be recorded in the corresponding section of the CRF, indicating the name, dose, timing, and indication for each medication.

### 6.5.1 Prohibited Concurrent Treatments

1. Oncology Medications: During the subject's participation in this study and throughout the research process, subjects will not simultaneously receive any anti-cancer treatments other than the investigational drugs of this clinical trial, including cytotoxic drugs, radiotherapy, other chemotherapies, biological therapies, immunosuppressants, prophylactic immunoenhancers, or any other investigational drugs with proven anti-cancer effects in clinical use.
2. Live Virus Vaccine Administration.

If the use of the aforementioned drugs is necessary during the study (e.g., for treatment of adverse events), the subject must discontinue study treatment and/or the study and inform the sponsor.

### 6.5.2 Permitted Concurrent Treatments

Investigators will record all concomitant medications used by subjects within 30 days prior to randomization and during the study in the corresponding section of the CRF.

1. Standard treatment for existing diseases, medical and/or surgical complications, and palliative care (excluding palliative radiotherapy), with all medications recorded.
2. Prophylactic medications for infusion reactions, and concomitant medications for adverse events as deemed necessary by the investigator.
3. The use of granulocyte colony-stimulating factors (G-CSF) is permitted when deemed necessary by the investigator during any treatment cycle, following the guidelines of the American Society of Clinical Oncology (ASCO).
4. Bisphosphonate treatment may be initiated during the study only under the following circumstances: on the one hand, for patients with confirmed new bone metastases or clear evidence of progression of existing bone metastases, and after confirmed disease progression; on the other hand, for patients where the treatment purpose is not for prevention or treatment of bone metastases, the purpose of treatment (e.g., anti-osteoporosis) should be recorded in the CRF.
5. Other: Prophylactic antiemetics, antidiarrheal treatments, antibiotic treatments, and basic medications for hypertension, etc., as deemed appropriate for concomitant use by the investigator.

## 6.6 Management of Drug Overdose

Drug overdose is defined as any dose exceeding the highest daily dose specified in the clinical study protocol. Any overdose treatment must be recorded in the investigational drug section of the CRF. There is no known antidote or pharmacological treatment for overdose of anti-EGFR monoclonal antibodies. Investigators should handle overdose based on their clinical experience, inform the sponsor promptly, consult on management, and file with the ethics committee. In the event of a serious adverse event, an SAE report should be filed.

## 6.7 Resection of Metastatic Lesions

Metastasectomy may be performed during the study when tumor regression has reached an optimal timing for surgical resection according to surgical standards. Resection of liver metastases has been shown to prolong survival in colorectal cancer patients, with a 5-year survival rate of 22-40% ^[27-29]^.

Surgery for metastatic lesions may be conducted if the following conditions are met:

- The surgery must be performed at least 2 weeks after the last study drug administration.
- Efficacy must be confirmed (i.e., a CT or MRI indicating effectiveness, followed by another CT or MRI at least 4 weeks later to confirm effectiveness).
- The purpose of the surgery is curative.

Surgical treatment must not cause the total interruption of study drug administration to exceed 10 weeks. For safety considerations, study treatment may not be resumed until at least 4 weeks after surgery.

Postoperative pathological results are defined as follows:

- R0 = No residual tumor after surgery (complete resection of all lesions).
- R1 = Incomplete resection of metastases, with microscopic residual tumor.
- R2 = Incomplete resection of metastases, with macroscopic residual tumor.

For subjects who achieve complete resection (R0) after surgery, it is permissible to continue study treatment under the same protocol for no more than 6 months. Even after treatment cessation, subjects should continue to be followed up every 8 weeks until disease progression. Only upon discovering progression should the subject then be followed up for survival every 3 months. Subjects who do not achieve complete resection (R1 or R2) after surgery should continue study treatment under the same protocol until disease progression.

The best response for the aforementioned subjects is the best response obtained before surgery. Postoperative subjects are no longer suitable for efficacy evaluation. To monitor disease progression, a new CT or MRI scan (using the same technology as the baseline scan) should be performed 4-6 weeks after surgery and before the resumption of study treatment, followed by scans every 8 weeks.

## 6.8 Other Study Precautions

After the study, all subjects should receive further treatment according to local clinical practices.

The investigational drug is prohibited for any purpose other than this study, and patients not included in this study should not use the study drug. If the study drug is used, the sponsor assumes no responsibility.

Palliative radiotherapy may be used for pain control or other non-curative purposes, such as treatment of bone metastases. Lesions within the radiation field should not be used for efficacy evaluation.

The following must be recorded on the CRF:

- All concomitant medications used by subjects within 30 days prior to signing the informed consent and during the study, including the name, dose, duration of use, and indication for each medication.
- The start and end dates of adjuvant chemotherapy received before enrollment in this study, treatment dosage, and protocol.
- All previous surgeries or treatment measures for the primary tumor (date, primary location).
- The dates, indications, records of any diagnostic, therapeutic, or surgical procedures performed during the study, and any clinical findings.
- Any changes in concomitant medications taken at the start of the clinical study, including the type of medication, dosage, duration of use, and indication.

# 7 Research Procedures and Evaluation

## 7.1 Evaluation Schedule

The research inspection items and time windows are detailed in the research flowchart.

Prior to the screening and enrollment of subjects: Investigators should explain the informed consent to the target patients who visit the clinic. Only after the patients fully understand the trial process and agree to participate in the trial, they should sign the informed consent form. Screening should be conducted according to the inclusion and exclusion criteria, and only those who meet the inclusion criteria and do not meet the exclusion criteria are eligible. The qualified subjects after screening will then undergo a baseline examination, including: demographic data, medication history, medical history, vital signs, physical examination, clinical indicators, laboratory tests, imaging, and electrocardiograms, etc. Subjects who pass all examinations will be assigned a drug number in the order of enrollment and will be randomly allocated to Group A or Group B to begin the trial.

### 7.1.1 Pre-screening Visit

The following content must be conducted and recorded before the screening visit:

- Signing of the written informed consent form
- Subject number assignment
- Diagnosis of tumor: date, histology, location of the lesion in the colon/rectum, and TNM staging at the time of diagnosis
- Archival tumor specimens required for RAS/BRAF testing

Note: The RAS/BRAF genotyping test is completed at this stage. Specimens for genetic testing are sent from each clinical trial center to the designated central laboratory for centralized testing. **Only patients with wild-type KRAS/NRAS are considered RAS wild-type patients, and those with both RAS/BRAF genotypes wild-type are eligible to enter the screening phase.**

### 7.1.2 Screening Visit

The following items must be executed and recorded:

Tests completed within 2 weeks prior to enrollment

- Inclusion and exclusion criteria
- Demographic data, including date of birth, gender, and ethnicity
- Medical history collection (relevant past diseases other than CRC)
- Diagnosis of the primary tumor (must record the date of diagnosis, histology, location of the lesion in the colon/rectum, and TNM staging at the time of diagnosis)
- Concurrent treatments and current medical interventions
- Assessment of previous treatments (including chemotherapy, adjuvant chemotherapy, radiotherapy, and surgery)
- Tumor markers, including Carcinoembryonic Antigen (CEA) and Carbohydrate Antigen 19-9 (CA19-9)
- Baseline tumor assessment with CT or MRI scans (tumor assessments conducted within 28 days prior to the first drug administration are allowed; assessments beyond 28 days cannot be used in this study)
- EORTC QLQ-C30 questionnaire
- Physical examination, with particular attention to the description of peripheral lymph nodes and palpable masses
- Vital signs (temperature, respiration, heart rate/pulse, and blood pressure [systolic and diastolic under resting conditions])
- ECOG score
- 12-lead electrocardiogram (ECG)
- Routine blood test
- Routine urine test
- Hepatic and renal function
- Electrolyte examination
- HIV antibody test
- Hepatitis B panel test and/or HBV-DNA test
- Pregnancy test (if applicable, premenopausal subjects undergo this test)
- Random grouping
- Assessment of adverse events

### 7.1.3 Treatment Visit

#### 7.1.3.1 Day 1 of Each Treatment Cycle

Each treatment cycle lasts for 14 days, including two administrations of anti-EGFR monoclonal antibody on Day 1 and Day 8. The following steps will be carried out on the first day.

- Weight measurement and calculation of BSA (within 3 days prior to administration)
- Administration of anti-EGFR monoclonal antibody (Group A only)
- Administration of chemotherapy (all subjects)
- 12-lead electrocardiogram (ECG) (tested once every 2 weeks for the first three treatment cycles, then once every 4 weeks; on the day of administration or within 3 days prior)
- Routine blood and urine tests, hepatic and renal function, and electrolytes (within 3 days prior to administration)
- Concurrent medications and current medical interventions
- Assessment of adverse events
- PK blood sample collection before administration (for subjects selected for PK testing)

Additionally, selected subjects will also undergo PK blood sample collection at non-treatment times, details are in section 7.6.

#### 7.1.3.2 Day 8 of Each Treatment Cycle (Group A only)

On Day 8 of each treatment cycle, the following steps will be carried out:

- Administration of anti-EGFR monoclonal antibody (Group A only)
- Routine blood test (within 3 days prior to administration)
- Concurrent medications and current medical interventions
- Assessment of adverse events
- PK blood sample collection before administration (for subjects selected for PK testing)

Additionally, selected subjects will also undergo PK blood sample collection at non-treatment times, details are in section 7.6.

#### 7.1.4 Evaluation Visit Every 8 Weeks

- Weight measurement
- CT or MRI scans for tumor efficacy assessment
- EORTC QLQ-C30 questionnaire
- CEA and CA19-9
- Vital signs
- Physical examination
- 12-lead electrocardiogram (ECG)
- Routine blood and urine tests, hepatic and renal function, and electrolytes
- Concurrent medications and current medical interventions
- Assessment of adverse events
- ECOG score

#### 7.1.5 Final Tumor Assessment Visit

- CT or MRI scans for tumor efficacy assessment
- EORTC QLQ-C30 questionnaire
- CEA and CA19-9
- Vital signs
- Physical examination
- 12-lead electrocardiogram (ECG)
- Routine blood and urine tests, hepatic and renal function, and electrolytes
- Concurrent medications and current medical interventions
- Assessment of adverse events
- ECOG score

#### 7.1.6 End-of-Trial Visit

The following assessments are conducted at the end-of-study visit:

- Items included in the end-of-trial assessment: completion date, last treatment date, subject's status at study end, reason for treatment discontinuation, and whether the disease progressed at the EOT visit.
- Concurrent medications and current medical interventions
- Collection of blood samples for ADA analysis (Group A only)
- Assessment of adverse events

#### 7.1.7 Follow-up

Follow-up will be conducted every 3 months via telephone, and the items to be assessed are as follows:

- Collection of tumor imaging data
- Subject's survival status
- Subsequent anti-tumor treatments
- Skin toxicity still present at the EOT visit (follow-up for up to 6 months)
- Follow-up of any study drug-related AEs until the AE is stable or the outcome is known.

## 7.2 Clinical Observation Indicators During the Study

#### 7.2.1 Clinical Observation Indicators

1. Physical examination, with particular attention to the description of peripheral lymph nodes and palpable masses;
2. Vital signs (temperature, respiration, blood pressure, and heart rate/pulse).

#### 7.2.2 Laboratory Examination Indicators

1. Complete blood count: Indicators measured include red blood cell (RBC) count, total and differential white blood cell (WBC) count, hemoglobin (Hb), and platelet (PLT) count.
2. Hepatic and renal function tests: Indicators measured include aspartate aminotransferase (AST), alanine aminotransferase (ALT), alkaline phosphatase (AKP), total bilirubin (TBIL), total protein (TP), albumin (ALB), blood urea nitrogen (BUN), and creatinine (Cr), uric acid (UA).
3. Urinalysis: Indicators measured include glucose (GLU), pH, protein (PRO), erythrocytes (BLD), and leukocytes (LEU).
4. Electrolytes: Indicators measured include potassium (K), sodium (Na), and magnesium (Mg).
5. Thoracoabdominal and pelvic examination: Thoracoabdominal and pelvic CT scans until disease progression (PD); if no measurable lesions are present, follow-up with chest X-ray, abdominal, and pelvic ultrasound examinations may be conducted.
6. Tumor markers: Indicators measured include carcinoembryonic antigen (CEA) and carbohydrate antigen 19-9 (CA19-9).
7. Immunogenicity testing: For subjects receiving anti-EGFR monoclonal antibody, blood samples are drawn before the first monoclonal antibody administration and at weeks 4, 8, 16, 32, and the end-of-study visit for anti-drug antibody (ADA) testing, totaling six blood collections.
8. Electrocardiogram (ECG): Electrocardiogram examinations are conducted once every 2 weeks for the first three cycles, then once every 4 weeks thereafter. (On the day of administration or within 3 days prior to administration)
9. Other: Other examinations such as head CT or MRI, bone ECT, etc., may be performed if deemed necessary by the investigator. The frequency of laboratory test indicators may be appropriately increased if deemed necessary by the investigator.

## 7.3 Efficacy Assessment

#### 7.3.1 Documentation of Tumor Assessment

Only subjects with RAS/BRAF wild-type status and measurable metastatic lesions are eligible for enrollment in the study. Measurable lesions must have at least one lesion that can be measured. Tumor assessments are conducted every 8 weeks (with a window of ±3 days), and comparisons should always be made with the initial dosing time without deferral or adjustment; if there is an overlap between the dosing visit window and the tumor assessment window, tumor assessment should be conducted first, and if effective, continue with the study treatment. If disease progression is suspected for any reason between the 8-week assessment visits, imaging confirmation is required unless there is a planned imaging examination within 14 days, and the last tumor assessment visit must include a CT or MRI scan. At the 8-week assessment visits, investigators should exclude symptom deterioration indicating disease progression (such as jaundice, skin metastases, etc.).

At baseline (within 4 weeks prior to the first dosing), **thoracoabdominal imaging** (CT or MRI) must be performed to assess eligibility for enrollment and serve as the baseline tumor assessment for subjects. If there is suspicion of cerebral metastasis in subjects, CT or MRI may be performed at discretion. The number of organs involved with metastatic lesions should be determined at baseline. The same assessment methods and techniques should be used to evaluate lesions during follow-up as at baseline. The only acceptable change in assessment technology is from CT to MRI for liver examination, which should not affect tumor assessment if the radiologist considers this change in assessment modality to be insignificant. Investigators or radiologists should maintain consistency in tumor assessment techniques whenever possible unless necessary.

CT and MRI are currently the most applicable and reproducible methods for detecting the efficacy of selected target lesions. Thoracoabdominal CT or MRI must be performed at baseline. Subsequent imaging examinations should include abdominal/pelvic CT or MRI. If baseline chest CT or MRI shows metastasis, chest CT or MRI should also be performed at each subsequent imaging examination. If baseline chest CT or MRI does not show metastasis, chest X-ray may be used for follow-up at each subsequent imaging examination. If suspicious metastatic lesions are indicated on chest X-ray during the study, chest CT or MRI should be performed for confirmation. Chest CT or MRI scans should be performed using spiral technology with a 5mm continuous reconstruction system. For non-spiral CT, the recommended reconstruction system or slice thickness ≤10mm is allowed, and intravenous or oral contrast should be given unless contraindicated.

Subjects who have undergone radical surgery for metastatic lesions are no longer suitable for efficacy assessment post-surgery. The best efficacy for these subjects is the best efficacy obtained pre-surgery.

All measured records should indicate the unit (in millimeters).

In assessing the primary endpoint PFS, the imaging data will be reviewed by a specially authorized IRaC in a blinded manner. Efficacy assessments and related treatment decisions during the study are jointly determined by the investigators and radiologists at the respective centers.

#### 7.3.2 Efficacy Evaluation Criteria

Investigators and IRaC will use the Response Evaluation Criteria in Solid Tumors (RECIST 1.1) (see Appendix 4) for efficacy assessment of subjects.

##### 7.3.2.1 Definition of Measurable and Non-measurable Lesions

To assess disease progression, the tumor burden measurement at baseline will be used for comparison with subsequent measurements. At baseline, lesions are defined as measurable and non-measurable.

**Measurable Lesions**

Tumor lesions: Must have at least one dimension that can be accurately measured (recorded as the longest diameter), with the minimum lengths as follows:

- CT scan ≥10 mm (CT scan slice thickness not greater than 5mm)
- Clinical routine examination instrument ≥10 mm (Tumor lesions that cannot be accurately measured with a measuring instrument should be recorded as non-measurable)
- Chest X-ray ≥20 mm

Malignant lymph nodes: Pathologically enlarged and measurable, the short diameter of a single lymph node on CT scan must be ≥15 mm (CT scan slice thickness is recommended not to exceed 5 mm). At baseline and follow-up, only the short diameter is measured and followed up.

**Non-measurable Lesions**

All other lesions, including small lesions (longest diameter <10 mm or pathological lymph node short diameter ≥10 mm to <15 mm) and lesions that cannot be measured. Non-measurable lesions include: meningeal disease, ascites, pleural or pericardial effusion, inflammatory breast cancer, cutaneous/pulmonary carcinomatous lymphangitis, abdominal masses that cannot be confirmed and followed up by imaging, and cystic lesions.

##### 7.3.2.2 Criteria for Target Lesions and Non-target Lesions

**Target Lesions**

When there are more than one measurable lesions at baseline assessment, all lesions should be recorded and measured, with a total number not exceeding 5 (no more than 2 per organ), as target lesions representing all involved organs (that is, patients with only one or two involved organs should select up to two or four target lesions as baseline measurement lesions at most).

Target lesions must be selected based on size (longest diameter), represent all involved organs, and measurements must have good repeatability. Sometimes, when the largest lesion cannot be repeatedly measured, another largest lesion that can be repeatedly measured may be reselected.

The pathological lymph nodes of target lesions must meet the following criteria: short diameter measured by CT ≥15 mm. Only the short diameter needs to be detected at baseline. Radiologists usually use the short diameter of the nodule to determine whether the nodule has tumor metastasis. The size of the nodule is generally represented by the two-dimensional data detected by imaging (the axial plane for CT, and one plane from the axial, sagittal, or coronal plane for MRI). The minimum value is the short diameter. For example, a 20 mm×30 mm abdominal nodule has a short diameter of 20 mm, which can be considered a malignant, measurable nodule. In this example, 20 mm is the measurement value of the nodule. Nodes with a diameter ≥10 mm but <15 mm should not be considered target lesions. Nodes <10 mm do not fall into the category of pathological nodes and do not need to be recorded or further observed.

**The sum of all target lesion diameters calculated (including the longest diameter of non-nodule lesions and the short diameter of nodule lesions) will be followed up as the baseline diameter sum.** If lymph node diameters are included, as mentioned above, only the short diameter is counted. The baseline diameter sum will serve as a reference value for the baseline level of disease.

**Non-target Lesions**

All other lesions, including pathological lymph nodes, can be considered non-target lesions, which do not need to be measured but should be recorded at baseline assessment. They should be noted as "present," "absent," and during follow-up, it should be indicated whether they are "present" or "disappeared."

##### 7.3.2.3 Efficacy Evaluation Criteria at Each Imaging Assessment Time Point

The overall efficacy will be confirmed based on the assessment results of target and non-target lesions, as well as the appearance of new lesions.

**The criteria for relief are shown in Tables 7.1 and 7.2.**

**Table 7.1 Evaluation of Target Lesions**

| Complete Response (CR) | Disappearance of all target lesions. Any pathological lymph nodes (whether target or non-target) must have reduction in short axis to < 10 mm |
| --- | --- |
| Partial Response (PR) | At least a 30% decrease in the sum of diameters of target lesions, taking as reference the baseline sum diameters |
| Progressive Disease (PD) | At least a 20% increase in the sum of diameters of target lesions, taking as reference the smallest sum on study (this includes the baseline sum if that is the smallest on study). In addition to the relative increase of 20%, the sum must also demonstrate an absolute increase of at least 5 mm. (Note: the appearance of one or more new lesions is also considered progression) |
| Stable Disease (SD) | Neither sufficient shrinkage to qualify for PR nor sufficient increase to qualify for PD, taking as reference the smallest sum diameters while on study |

**Table 7.2 Evaluation of non-target lesions**

| Complete Response (CR) | Disappearance of all non-target lesions and normalisation of tumor marker level. All lymph nodes must be non-pathological in size (<10 mm short axis) |
| --- | --- |
| Non-CR/Non-PD | Persistence of one or more non-target lesion(s) and/or maintenance of tumor marker level above the normal limits |
| Progressive Disease (PD) | Unequivocal progression of existing non-target lesions |

If a subject discontinues treatment due to deterioration of overall health status but lacks objective evidence of disease progression at that time, it should be classified as "symptomatic deterioration." Despite discontinuation of treatment, efforts should still be made to obtain objective evidence of disease progression.

All possible combinations of overall tumor efficacy evaluations are presented in Table 7.3. A mid-overall efficacy evaluation is required each time a planned continuation of efficacy assessment is scheduled. The possible best overall efficacies at various time points are Complete Response (CR), followed by Partial Response (PR), Stable Disease (SD), and Progressive Disease (PD).

**Table 7.3 Time point response: patients with target (+/–non-target) disease**

| **Target lesions** | **Non-target lesions** | **New lesions** | **Overall response** |
| --- | --- | --- | --- |
| CR | CR | No | CR |
| CR | Non-CR/Non-PD | No | PR |
| CR | Not evaluated | No | PR |
| PR | Non-PD or not all evaluated | No | PR |
| SD | Non-PD or not all evaluated | No | SD |
| Not all evaluated | Non-PD | No | NE（inevaluable）* |
| PD | Any | Yes or No | PD |
| Any | PD | Yes or No | PD |
| Any | Any | Yes | PD |

* If subsequent assessment results are PD, then the overall efficacy is PD

##### 7.3.2.4 Confirmation of Efficacy

To confirm Complete Response (CR) or Partial Response (PR), the change in tumor size should be reassessed using the same imaging technology at least 4 weeks (28 days) after the initial achievement of the efficacy criteria. Confirmatory imaging for CR or PR is permitted during a planned every-8-weeks imaging visit following the achievement of CR or PR. If the assessment result is Stable Disease (SD), the tumor size must have met the SD criteria on at least one occasion during the follow-up period and should be conducted at least 6 weeks (42 days) after the baseline assessment.

According to the RECIST 1.1 criteria, there is no need for a repeat imaging confirmation for Progressive Disease (PD) recorded in imaging. For subjects with borderline assessment results, one additional cycle of study treatment may be administered followed by a repeat imaging assessment. If the subsequent assessment confirms disease progression, the date of progression should be the date when suspected progression was first noted. Study treatment should not be discontinued based on a clinical diagnosis (unconfirmed) of PD.

##### 7.3.2.5 Assessment of Best Overall Efficacy

The definition of the best overall efficacy for a subject is the best confirmed efficacy observed at all time points of efficacy assessment. The possible best overall efficacy results are CR, followed by PR, SD, and PD.

The confirmation steps for the potential best efficacy at two time points are summarized in Table 7.4. Except for CR, the two time points do not need to be consecutive.

**Table 7.4 Best overall response when confirmation of CR and PR required**

| **Overall response First time point** | **Overall response Subsequent time point** | **BEST overall response** |
| --- | --- | --- |
| CR | CR | CR |
| CR | PR | SD, PD or PR ^a^ |
| CR | SD | SD provided minimum criteria for SD duration met, otherwise, PD |
| CR | PD | SD provided minimum criteria for SD duration met, otherwise, PD |
| CR | NE | SD provided minimum criteria for SD duration met, otherwise NE |
| PR | CR | PR |
| PR | PR | PR |
| PR | SD | SD |
| PR | PD | SD provided minimum criteria for SD duration met, otherwise, PD |
| PR | NE | SD provided minimum criteria for SD duration met, otherwise NE |
| NE | NE | NE |

CR = complete response, PR = partial response, SD = stable disease, PD = progressive disease, and NE = inevaluable.

a If a CR is truly met at first time point, then any disease seen at a subsequent time point, even disease meeting PR criteria relative to baseline, makes the disease PD at that point (since disease must have reappeared after CR). Best response would depend on whether minimum duration for SD was met. However, sometimes ‘CR’ may be claimed when subsequent scans suggest small lesions were likely still present and in fact the patient had PR, not CR at the first time point. Under these circumstances, the original CR should be changed to PR and the best response is PR

##### 7.3.2.6 Independent Radiology Review Committee

During the study, the investigators will determine the best efficacy for all subjects based on the RECIST criteria, version 1.1. An Independent Radiology Review Committee (IRaC) will conduct a blinded review of all subjects' imaging results using the same criteria, following an independent audit procedure throughout the review process.

#### 7.3.3 Efficacy Assessment

##### 7.3.3.1 Primary Efficacy Indicators

**Progression-Free Survival (PFS)**

The PFS duration (in months) is determined by a specially authorized Independent Radiology Review Committee (IRaC) through blinded review of imaging data. It is defined as the time from randomization to the first confirmed progression of disease by imaging or death from any cause within 90 days after the last tumor assessment or randomization, whichever is later (equivalent to 1.5 times the interval between two consecutive tumor assessments).

Tumor progression and response are assessed according to RECIST criteria version 1.1. Tumor assessments are conducted every 8 weeks after randomization until the end of the study.

If a subject has neither tumor progression nor death after the last tumor assessment in the study or within 90 days after randomization, they are censored at the date of the last tumor assessment. If a subject has not received study treatment and has not progressed or died, they are censored at the date of randomization (the first day of the study). Information defining PFS will be detailed in the Statistical Analysis Plan (SAP).

##### 7.3.3.2 Secondary Efficacy Indicators

1. **Objective Response Rate (ORR)**

Investigators will use the RECIST 1.1 criteria to assess the objective response rate. Detailed information on target and non-target lesions will be collected in the Case Report Form (CRF) to calculate tumor objective response. Post-baseline tumor assessments should be conducted using the same evaluation methods (CT, MRI) as at baseline and preferably at the same hospital.

The objective response rate is calculated as the percentage of evaluable subjects with complete response (CR) and partial response (PR) (ORR = CR + PR).

1. **One-year Overall Survival Rate, Two-year Overall Survival Rate, and Overall Survival Time**

One-year overall survival rate definition: The proportion of subjects alive at one year from randomization.

Two-year overall survival rate definition: The proportion of subjects alive at two years from randomization.

Overall survival time: Defined as the time from randomization to death (in months). For subjects still alive or lost to follow-up as of the data analysis cutoff date, survival is censored at the subject's last known alive time.

1. **Disease Control Rate (DCR)**

Refers to the percentage of subjects with the best response of complete response (CR), partial response (PR), and stable disease (SD) according to RECIST 1.1 criteria (DCR = CR + PR + SD).

1. **Clinical Benefit Rate (CBR)**

The proportion of subjects with the best tumor assessment of complete response (CR), partial response (PR), and stable disease (SD) that lasts for 24 weeks or more according to RECIST 1.1 criteria.

1. **Duration of Response (DOR)**

For subjects with the best response of CR or PR according to RECIST 1.1 criteria, the time from the first occurrence of (CR or PR) to the first occurrence of disease progression or death. If a subject has not experienced an endpoint event, they are censored, with the censored date being the last imaging assessment date.

1. **Time to Response (TTR)**

For subjects with the best response of CR or PR according to RECIST 1.1 criteria, the time from randomization to the first occurrence of response (CR or PR) according to RECIST 1.1.

1. **Quality of Life Assessment Indicators**

Quality of life assessment (QOL) is conducted using the EORTC QLQ-C30 questionnaire (Appendix 5), with individual item categorical scores linearly transformed to a 0-100 scale. The questionnaire is self-reported, consisting of 30 items, and subjects are asked to complete it carefully. Assessments are conducted at baseline and subsequent visits every 8 weeks until withdrawal from the study and entry into the follow-up period.

1. **Resection Rate of Hepatic Metastasis**

The resection rate of hepatic metastasis is calculated as the number of subjects achieving complete resection (R0 resection) divided by the total number of subjects.

## 7.4 Safety Evaluation Criteria

#### 7.4.1 Safety Evaluation Criteria

The Common Terminology Criteria for Adverse Events (CTCAE) version 4.03 is adopted as the standard for defining adverse events and grading their severity.

#### 7.4.2 Safety Evaluation Indicators

The following changes occurring during the trial period, after signing the informed consent form:

- Vital signs: Clinically significant changes in heart rate/pulse, respiration, body temperature, and blood pressure;
- Laboratory parameters: Clinically significant changes in routine blood and urine tests, hepatic and renal function, and chest X-rays;
- Any other clinically significant deterioration in pre-existing conditions.

## 7.5 Adverse Events

#### 7.5.1 Definition of Adverse Events

##### 7.5.1.1 Adverse Events

An Adverse Event (AE) is any undesirable medical event that occurs in the course of a clinical study that is not necessarily related to the investigational product, including but not limited to:

- Any clinically significant worsening of a pre-existing condition;
- AEs resulting from overdose of the investigational product, whether intentional or unintentional. Overdose is defined as the administration of a dose exceeding that specified in the protocol;
- AEs resulting from abuse (e.g., non-clinical use) of the investigational product;
- AEs associated with the discontinuation of the investigational product;
- Unanticipated medical conditions that may be symptoms (e.g., nausea, fever, etc.), signs (e.g., tachycardia, etc.), or abnormal results of diagnostic tests (e.g., laboratory tests, electrocardiogram, etc.).

Note: The progression of metastatic colorectal cancer as the underlying disease will be evaluated as an efficacy measure and not recorded as an adverse event.

##### 7.5.1.2 Serious Adverse Events

Serious Adverse Events are any adverse events that occur and result in targeted medical interventions (such as drug withdrawal, dose reduction, and symptomatic treatment) and significant abnormalities in hematological or other laboratory tests, excluding Serious Adverse Events.

##### 7.5.1.3 Serious Adverse Events (SAEs)

Serious Adverse Events (SAEs) are adverse events that occur at any stage of the study (including the screening or washout period, treatment period, and follow-up period), with any dose of the investigational product and control product, and meet one or more of the following criteria:

1. Results in death;
2. Is life-threatening;
3. Requires hospitalization or prolongs hospital stay;
4. Results in persistent or significant disability/function loss;
5. Is a congenital anomaly/birth defect;
6. Is an event of significant medical importance.

Events of significant medical importance may not result in death, be life-threatening, or require hospitalization, but are considered SAEs if they result in harm to the subject and require medical or surgical intervention to prevent one of the outcomes listed above, based on medical judgment.

Some events that require hospitalization or prolong hospital stay are not considered SAEs, including: 1. Hospitalization for social reasons rather than adverse events; 2. Hospitalization for elective surgery, tests, or other treatments that were scheduled prior to entering the trial.

Note: Death is considered the end outcome of an event. If death occurs, the primary cause of death (the main reason for death) will be recorded and reported as an SAE. Death is not recorded as a separate event. Only when the cause of death cannot be determined (e.g., sudden death, death from unknown causes) should death itself be reported as an SAE.

##### 7.5.1.4 Adverse Reactions

For drugs that have not yet been approved for marketing, an adverse reaction is any discomfort and unexpected reaction associated with the use of the drug at any dose. That is, there is a causal relationship between the adverse reaction and the use of the drug, at least a reasonable possibility. The severity of adverse reactions can be judged by the investigators according to the safety evaluation criteria.

##### 7.5.1.5 Suspected Unexpected Serious Adverse Reactions (SUSARs)

Suspected Unexpected Serious Adverse Reactions (SUSARs) refer to adverse drug reactions that have a nature or severity of clinical presentation not included in the Investigator's Brochure, the product information for marketed drugs, or the summary of product characteristics.

#### 7.5.2 Assessment of the Severity of Adverse Events

The severity of adverse events is judged according to the safety evaluation criteria and severity grading of CTCAE version 4.03.

Investigators should assess the severity of adverse events through clinical judgment, rather than directly through the subject's experience (e.g., abnormal laboratory test results).

#### 7.5.3 Assessment of Causality between Adverse Events and Medication

In China and internationally, the causality between adverse events and medication is usually divided into five situations:

1. Definite Related: The occurrence of AE is highly likely to be caused by the drug;
2. Probably Related: The occurrence of AE may be caused by the use of the drug;
3. Possibly Related: The occurrence of AE may be caused by the drug;
4. Unlikely to be Related: The occurrence of AE is more likely related to another factor;
5. Not Related: The AE is caused by another significant factor.

The above items ① to ③ should be recorded as adverse reactions to the drug. The incidence rate of adverse reactions = (①+②+③) the sum of the number of cases / the number of cases in the safety data set × 100%.

**Causal Relationship of the Adverse Event to the Study Treatment**

|  | ①  Definite Related | ②  Probable Related | Possibly Related | ④  Unlikely to be Related | ⑤  Not Related |
| --- | --- | --- | --- | --- | --- |
| Temporal Relationship: There exists a plausible temporal association between the administration of the medication and the occurrence of the reaction/event | + | + | + | + | － |
| Consistency with Known Adverse Reactions: The reaction/event is consistent with the known adverse reaction profile of the medication | + | + | + | － | － |
| Dose-Response Relationship: The reaction/event subsides or diminishes upon discontinuation or reduction of the medication | + | + | ± | ± | － |
| Rechallenge: The reaction/event reoccurs upon re-administration of the medication | + | ? | ? | ? | － |
| Unexplained by Alternative Theories: The reaction/event cannot be explained by the pharmacological action of the medication, the progression of the patient's condition, or the influence of other treatments | + | + | － | ± | － |

#### 7.5.4 Events Not Meeting the Definition of SAE

Hospitalizations for study treatment or elective study procedures are not considered adverse events. However, any prolongation of an elective hospital stay that is not planned (such as discomfort due to any treatment) must be reported as an SAE.

Events not considered AEs/SAEs:

Diseases present at the study screening visit, which do not increase in severity or frequency during the study period, should be defined as baseline medical conditions rather than adverse events.

The progression of the primary disease (tumor) is not an AE or SAE but an efficacy endpoint. However, there are two exceptions: first, when the disease (tumor) progression is accompanied by symptoms and signs that meet the criteria for AE/SAE, it should be reported as an AE/SAE; second, any death due to tumor progression during the reporting period should be reported as an SAE.

#### 7.5.5 Recording of Adverse Events

All AEs/SAEs must be recorded in the corresponding section of the CRF. If an SAE occurs, a Serious Adverse Event Report form (initial or follow-up) should also be completed. Specific guidelines can be found in the SAE Report Form instructions.

The following information for each AE should be recorded in the CRF:

- AEs should be described using medical terminology, not the subject's complaints;
- Date of onset (start date);
- Date of recovery (end date);
- Grade assessed and severity judged by the investigator according to NCI-CTCAE version 4.03 (Yes/No);
- Causality assessment of the adverse event in relation to the medication (separate assessments for anti-EGFR and chemotherapy drugs);
- Impact on treatment (No change/Continue treatment, Dose reduction, Temporary hold/Dose delay, Permanent discontinuation, Not applicable);
- Measures taken (None, Concurrent medication, Concurrent medical procedures, leading to study discontinuation);
- Outcome to be recorded with the following information:

1. Resolution of symptoms without sequelae;
2. Resolution of symptoms with sequelae;
3. Ongoing symptoms;
4. Death (AE resulted in death);
5. Unknown.

#### 7.5.6 Definition of the Adverse Event Reporting Period

For safety monitoring, the AE reporting period begins when a subject is enrolled in the clinical study (the date of signing the informed consent form) and continues until the end of the post-study follow-up period, up to and including the EOT visit. For AEs still present at the EOT visit, refer to section 7.5.8 for details.

#### 7.5.7 SAE Reporting Process

In the event of an SAE during the trial, the investigator should first ensure timely and appropriate management or emergency treatment, and second, immediately report the SAE to the sponsor in writing, followed by a timely and detailed written follow-up report. For reports involving death, the investigator should provide any additional required information to the sponsor and the ethics committee, such as autopsy and final medical reports.

The sponsor is responsible for safety monitoring throughout the entire study conduct and must ensure that the study centers comply with regulatory agency and local regulations for reporting all SAEs. Additionally, the sponsor must report SAEs to the appropriate regulatory agencies as required by regulatory authorities and local regulations.

#### 7.5.8 Reporting Process for Suspected Unexpected Serious Adverse Reactions (SUSARs)

Investigators should report to the ethics committee any SUSARs provided by the sponsor.

The sponsor should rapidly report SUSARs to all investigators and clinical trial institutions, ethics committees involved in the clinical trial; at the same time, the sponsor should report SUSARs to the drug regulatory authority and the health authority.

#### 7.5.9 Development Safety Update Report (DSUR) Reporting Process

During the clinical trial period, the sponsor should conduct a comprehensive and in-depth annual review, summary, and evaluation of all drug-related safety information collected during the reporting period and submit the Development Safety Update Report (DSUR) to the drug regulatory authority on time.

The DSUR provided by the sponsor should include a risk-benefit assessment of the clinical trial, and relevant information should be communicated to all investigators and clinical trial institutions, ethics committees involved in the clinical trial.

#### 7.5.10 Monitoring of Adverse Events

For skin reactions still present at the EOT visit, continued monitoring should be conducted to determine their final outcome and the resolution date of these adverse events (not to exceed six months). For any AE that occurs during the trial and is considered related to at least one study drug, it must be monitored and followed up by the investigator until the condition is stable or the outcome is known, unless the subject is "lost to follow-up." Investigators should make reasonable efforts to obtain this information and record it. Investigators must ensure that necessary additional treatments and follow-up measures are taken. SAEs must be followed up until they are stable or the outcome is known.

#### 7.5.11 Pregnancy and In Utero Drug Exposure

Only pregnancies deemed by the investigator to be related to study treatment (such as those resulting from interactions with contraceptives) are considered adverse events. However, if the estimated date of conception is within the adverse event reporting period, it must be recorded in the AE page/section of the CRF as routine. The above applies to pregnancies in female subjects and the pregnancies of male subjects' female partners. Investigators must use the pregnancy report form to inform the sponsor and ethics committee quickly, following the steps for reporting SAEs.

Investigators must actively follow up, document, and report the outcome of these pregnancy events, even if the subject has withdrawn from the study.

Investigators must use the pregnancy report form to report the outcome of the event to the sponsor. In cases of abnormal outcomes, such as abnormalities in the subject, the Serious Adverse Event Report form should be used, and for abnormalities in the child/fetus, the Maternal-Child/Fetal Adverse Event Report form should be used. Abnormal outcomes must be reported within 45 days after delivery.

If a female subject becomes pregnant during the study, the subject must immediately discontinue trial medication. The sponsor should be notified immediately, and the subject should be followed up as required above.

## 7.6 Pharmacokinetics

The PK population consists of subjects who receive anti-EGFR monoclonal antibody treatment and sign the ICF agreeing to participate in the PK study. A minimum of 8 subjects is required to participate in the PK study, with a maximum of no more than 24. These subjects will be selected in advance to avoid bias. At the time of signing the informed consent form, subjects must be explained that only those selected in advance will undergo these tests. In addition, subjects must be informed that this PK analysis is a necessary part of this clinical study. Subjects who agree to participate in the study and are within the selected range must agree to participate in the PK study. If a selected subject cannot complete the PK assessment, they should be replaced by another subject.

Serum concentration collection of anti-EGFR monoclonal antibody will be conducted at the designated center. If a selected subject cannot complete the PK assessment, they should be replaced by another subject.

#### 7.6.1 Pharmacokinetic Analysis Blood Collection Schedule

The blood collection schedule is as shown in the table below, and the time of each blood collection must be recorded in detail on the blood collection form.

**First dose administration**

|  | Day 1 | | | | | Day 2 | Day 3 | Day 4 | Day 5 | Day 8 |
| --- | --- | --- | --- | --- | --- | --- | --- | --- | --- | --- |
| Time | Pre Dose | 0h* | 3h | 6h | 12h | 24h | 48h | 72h | 96h | 168** |
| Sample ID | PK000 | PK001 | PK002 | PK003 | PK004 | PK005 | PK006 | PK007 | PK008 | PK009 |

**Weekly dosing**

|  | Day 15 | Day 22 | Day 29 | Day 36 | Day 43 | Day 50 |
| --- | --- | --- | --- | --- | --- | --- |
| Time | Pre Dose | Pre Dose | Pre Dose | Pre Dose | Pre Dose | Pre Dose |
| Sample ID | PK0010 | PK0011 | PK0012 | PK0013 | PK0014 | PK0015 |

**Dosing starting from week 4**

|  | Day 22 | | | | | Day 23 | Day 24 | Day 25 | Day 26 | Day 29 |
| --- | --- | --- | --- | --- | --- | --- | --- | --- | --- | --- |
| Time | Pre Dose | 0h* | 3h | 6h | 12h | 24h | 48h | 72h | 96h | 168 |
| Sample ID | PK0011 | PK101 | PK102 | PK103 | PK104 | PK105 | PK106 | PK107 | PK108 | PK0012 |

Note: * "0h" refers to the time at the end of the infusion; ** "168h" refers to 168 hours after the initial administration of the drug, but before the administration of the anti-EGFR monoclonal antibody on the same day.

#### 7.6.2 Pharmacokinetic Calculations

The pharmacokinetic (PK) parameters of the anti-EGFR monoclonal antibody are calculated using the non-compartmental analysis method with WinNonlin software. PK parameters include: C_min_, T_max_, C_T_, t_1/2_, AUC_0-t_, CL, and MRT, among others.

## 7.7 Immunogenicity

Blood samples are collected at the following time points: before dosing in Week 1, and before dosing in Weeks 4, 8, 16, and 32, as well as at the end-of-study visit, totaling six blood collections.

Each clinical trial center collects 2 to 5 milliliters of whole blood, from which serum is separated by centrifugation. The collected serum is stored in sealed, capped serum tubes, labeled with: (1) subject identification number; (2) blood collection sequence number (e.g., T0=baseline, T1=Week 4, T2=Week 8, T3=Week 16, T4=Week 32, T5=EOT); (3) collection tube identification number. The samples are stored at a temperature of ≤-20°C and shipped to the designated central laboratory for unified testing in accordance with the "Central Laboratory Manual." Blood collection time points may change due to alterations in the treatment schedule (e.g., dosing delays and/or interruptions).

Blood samples are used to determine the changes in the production of anti-CMAB009 antibodies in subjects after treatment. For subjects who test positive for anti-drug antibodies (ADA), the levels of neutralizing antibodies (Nab) will be further assessed.

# 8 Data Quality Assurance

To ensure the integrity, accuracy, and reliability of data, the following measures are taken in this study:

1. Select qualified and experienced research units and investigators;
2. Provide a detailed introduction to the content of the trial protocol to researchers before the start of the study through training sessions and written materials, and collectively develop solutions for potential issues;
3. Regularly verify the authenticity, accuracy, and completeness of data by monitors;
4. Communicate with researchers in a timely manner and confirm or correct data if any questions arise;
5. Check data during the statistical phase. If discrepancies are found, researchers should confirm or correct them.

## 8.1 Data Recording

#### 8.1.1 Medical Record Completion

During the study, medical records are kept using the format provided by each research center. The medical record should include: the subject's real name, subject number, gender, age, contact information, etc., and should detail the subject's demographic information, medical and medication history, vital signs, physical examination, clinical indicators, adverse reactions and their management, concomitant medication, etc.; the back cover is generally for pasting all laboratory test reports.

The subject's medical record, as the original document of the clinical trial, should be kept complete. The medical record is the responsibility of the investigator to fill in and keep, and the investigator should check the subject's name on the medical record before each entry. The writing should be neat and legible, facilitating the monitor's data verification with the CRF during each visit.

#### 8.1.2 eCRF Completion and Review

Data recorded during the study needs to be recorded on the eCRF, and these anonymized data should be processed, evaluated, and stored in accordance with data confidentiality regulations. The investigator or their designated personnel are responsible for entering data into the electronic case report form (eCRF). Ensuring the accuracy of the data entered into the eCRF is the responsibility of the investigator.

These data will be entered into a confirmed database. The data management department of the sponsor or the designated CRO will be responsible for data processing under the supervision of the sponsor, following the designated data management procedures. Once the quality assurance procedures are completed, the database will be closed. A PDF file of the eCRF will be provided to the investigator upon completion of the study.

## 8.2 Data Monitoring

Throughout the clinical trial process, the sponsor designates qualified monitors to regularly conduct on-site monitoring at each clinical trial center to ensure that all aspects of the trial protocol are strictly followed. Each trial center should assist and cooperate with the monitor's work.

Implementers: Monitors, Data Managers.

The content of the monitoring mainly includes: adherence to the trial protocol; whether clinical trial materials, equipment, personnel, and sites are adequate, normally used, and whether there have been any changes; whether the signing of informed consent forms meets the requirements; whether adverse events are correctly recorded and serious adverse events are reported in a timely manner; the storage and management of trial medication; the recording and storage of related documents, etc. Whether all CRF forms are filled out correctly and completely, and whether they are consistent with the original documents such as medical records and laboratory test reports, and whether there are any errors or omissions in the data. Since most of the data in the CRF forms are transcribed from original data such as medical records and laboratory test reports, most of the content is second-hand information. Therefore, during monitoring, the content in the CRF forms needs to be repeatedly checked against the original documents to ensure that the data in the CRF forms are completely consistent with the original data. This process is also known as source data verification (SDV).

#### 8.2.1 Audits and Inspections

Throughout or after the clinical trial, the sponsor may conduct audits, and the government drug regulatory authority may conduct inspections, and each clinical trial center should cooperate.

The content of the audit mainly includes: whether there are all the files required by GCP; verification of original data and case report forms (CRF); inspection of equipment; inspection of drug storage and management; inspection of the fulfillment of monitor responsibilities; meeting with relevant personnel involved in the clinical trial, etc.

#### 8.2.2 Data Management

The most commonly used form of data collection and transmission in clinical trials is EDC. The investigator fills in, the monitor checks, the study leader reviews, and the data manager verifies. Finally, the data manager establishes a database based on all the indicators and values in the submitted CRF forms.

The data manager formulates data checks according to the range and interrelationship of the indicator values in the CRF form, such as range checks and logic checks. If any problems are found, the monitor should be notified in a timely manner, and the investigator should be asked to respond. The exchange of questions and answers between the investigator, monitor, and data manager should be recorded using a query form and kept for reference. Data is handed over to the statistical party for data analysis.

After all trial studies are completed, each center's clinical institution retains some research materials, and the sponsor archives all research materials for reference.

The preservation and management of trial materials must be carried out in accordance with regulations. The investigator should retain the clinical trial materials for at least five years after the termination of the clinical trial, and the sponsor should retain the clinical trial materials for at least five years after the trial drug is approved for marketing.

# 9 Statistical Analysis

## 9.1 Sample Size

Based on updated data from the CRYSTAL study in the literature, the median PFS for RAS wild-type subjects treated with (Cetuximab combined with FOLFIRI) and FOLFIRI monotherapy are 11.4 months and 8.4 months, respectively. This study is expected to enroll for 20 months, with a total study period of 36 months, a significance level of α=0.025 (one-sided), and requires observation of 339 events (PD) to detect a risk ratio of 0.7368 with 80% power. With a 1:1 ratio, the sample size is calculated to be 410 cases using PASS 13 sample size estimation software, considering a 20% dropout rate, the planned number of cases is 256 for Group A and 256 for Group B, totaling 512 cases.

## 9.2 Endpoint Indicators

#### 9.2.1 Primary Efficacy Endpoint

Primary endpoint indicator: PFS time (months)

#### 9.2.2 Secondary Efficacy Endpoints

Secondary efficacy indicators include:

- Objective Response Rate (ORR)
- One-year overall survival rate, two-year overall survival rate, and overall survival time (OS)
- Disease Control Rate (DCR)
- Clinical Benefit Rate (CBR)
- Duration of Response (DOR)
- Time to Response (TTR)
- Quality of Life Evaluation (QOL)
- Resection rate of hepatic metastatic lesions

#### 9.2.3 Safety Endpoint Indicators

- Drug exposure
- All types of AEs and incidence rates
- Mortality rate and causes of death
- Safety laboratory tests
- Vital signs

#### 9.2.4 Other Study Endpoints

- Immunogenicity (ADA)
- Pharmacokinetics

The study will also analyze concomitant medications taken at any time during the study or within 30 days after the last study drug administration.

## 9.3 Statistical Analysis Plan

Drafted by the person in charge of statistical analysis and agreed upon with the principal investigator, the plan is formulated after the protocol and CRF are established. It may be modified, supplemented, and improved during the trial process but must be confirmed in writing before unblinding and not changed afterward. The Statistical Analysis Plan should determine the selection of analysis datasets, statistical analysis methods, primary and secondary indicators, efficacy and safety evaluation methods, etc., and list statistical analysis tables for expected results.

## 9.4 Analysis Datasets

#### 9.4.1 Full Analysis Set and Population

According to the basic principles of Intention-To-Treat (ITT) analysis, the analysis of primary efficacy indicators should include all randomized subjects, that is, all randomized subjects should have complete follow-up of study results, which is often difficult in practice. Therefore, this trial uses the Full Analysis Set (FAS) for analysis.

The Full Analysis Set refers to an ideal subject set that is as close as possible to the ITT principle. This dataset is derived from all randomized subjects, with the least and reasonable method to exclude certain cases.

The ITT population will include all subjects who are randomly entered into the study and have received at least one dose of the study treatment. Subjects randomized will be analyzed.

#### 9.4.2 Per-Protocol Set and Population

The Per-Protocol Set (PPS) refers to a collection of cases that meet the trial protocol provisions, have good compliance, and have completed all required data collection, also known as "evaluable cases" or "qualified cases" samples. It is a subset of the FAS. Subjects in this dataset are more compliant with the protocol, should have completed the predetermined minimum amount of treatment, have measurement results for primary variables, and have no major violations of the trial protocol. Cases that violate the trial protocol, such as those who have not reached the specified treatment amount, have missing primary variables, or have used explicitly prohibited drugs, are not included in this dataset, and the reasons for excluding cases from this dataset should be explained.

The Per-Protocol population will not include all ITT populations that meet one or more of the following criteria:

- Randomization failure (e.g., receiving incorrect treatment)
- No evidence of mCRC at baseline (e.g., no target lesions with the longest diameter ≥1cm at baseline)
- Receiving study treatment for ≤8 weeks, excluding subjects who die or have PD within the first 8 weeks after starting treatment
- Receiving unauthorized chemotherapy or other treatments during the study period

#### 9.4.3 Safety Dataset and Population

The Safety Dataset (SS) refers to all subjects who have received at least one dose of treatment after randomization, that is, as long as the subject has used the prescribed group of drugs once or more, regardless of whether the subject is included in the Per-Protocol Set, their adverse events and reactions should be analyzed. Cases that have not taken any study medication or have no follow-up observation data after enrollment are excluded.

#### 9.4.4 Responder Evaluable Population

The Responder Evaluable Set (RES) is a subset of the FAS, referring to subjects in the Full Analysis Set who have at least one post-baseline tumor assessment;

#### 9.4.5 PK Population

The PK Parameter Set (PKPS): Subjects who provide PK blood collection according to the protocol, a designated population of Group A subjects. Subjects who have received at least one dose of the study drug and have at least one pharmacokinetic parameter. The PKPS is used to descriptively statistically analyze the pharmacokinetic parameter data of subjects.

#### 9.4.6 Immunogenicity Analysis Set

The Immunogenicity (ADA) Analysis Set: All enrolled patients who have used the study drug at least once and have baseline and at least one post-baseline ADA evaluation data.

## 9.5 Statistical Analysis Methods

Statistical analysis will be conducted using SAS 9.4 or later version statistical analysis software. All statistical tests in this study will be two-sided, and a P-value less than 0.05 will be considered to indicate a statistically significant difference. Descriptive statistics will be the primary method of analysis, utilizing measures such as mean, standard deviation, median, minimum, and maximum values for continuous variables; counts and percentages for categorical variables.

1. Baseline Analysis

Baseline analysis will utilize the Full Analysis Set (FAS) dataset. Inter-group comparisons of baseline indicators such as demographic characteristics and medical history will be performed using t-tests or Wilcoxon rank-sum tests for continuous data, and Fisher's exact test for categorical data. Adverse events will be coded using Medical Dictionary for Regulatory Activities (MedDRA) terminology. Comparisons with screening baseline values will be made using paired t-tests to assess within-group differences before and after.

1. Analysis of Enrollment and Excluded Cases

The analysis will primarily be descriptive, detailing the enrollment and completion of cases at each center, as well as the distribution of dropouts and excluded cases. The demographic distribution of the study population at each center will also be described in tables.

1. Efficacy Analysis

**The primary endpoint is PFS time (months).**

A superiority design will be employed, with hypothesis testing as follows:

H0: HT/HC ≤ 1

H1: HT/HC > 1

α = 0.025 (one-sided)

Analysis of PFS between the two groups will utilize the Logrank test, stratified by center and ECOG score as covariates. The Cox proportional hazards model will be used to estimate the hazard ratio (HR) and its 95% confidence interval (CI) between the experimental and control groups. If the lower limit of the 95% CI is greater than 1, the combination therapy of anti-EGFR monoclonal antibody + FOLFIRI is considered superior to FOLFIRI monotherapy. Kaplan-Meier curves of PFS will be plotted for each treatment group.

**Secondary efficacy indicators include**

Objective Response Rate (ORR), one-year overall survival rate, two-year overall survival rate, overall survival time (OS), Disease Control Rate (DCR), Clinical Benefit Rate (CBR), Duration of Response (DOR), Time to Response (TTR), and Quality of Life Evaluation (QOL).

For ORR, one-year overall survival rate, two-year overall survival rate, DCR, and CBR, a Logistic regression model will be used for analysis, with covariates (center, ECOG score) adjusted to calculate the odds ratio and its 95% confidence interval for each group.

For DOR and OS, the survival function will be estimated using the Kaplan-Meier method, and survival curves will be plotted. The Logrank test will be performed between the two groups. The Cox proportional hazards model will be used to calculate the hazard ratio and its 95% confidence interval between treatment groups, with covariates (center, ECOG score) specified.

For TTR, only subjects who have achieved a response and have no censored data will be included. The analysis will be conducted as a continuous variable using a linear regression model, with covariates (center, ECOG score) adjusted to calculate the difference and its 95% confidence interval between the two groups.

For Quality of Life Evaluation scores, comparisons between the two groups will be made using t-tests or Wilcoxon rank-sum tests.

Efficacy analysis should be performed separately for the FAS and Per-Protocol Set (PPS) datasets. Inconsistencies between the two will require further analysis and explanation.

1. Safety Analysis

The Safety Set (SS) dataset will be used, with a focus on descriptive statistical analysis. Adverse events, laboratory tests, and vital signs will be listed and described, along with the determination of their association with the trial medication. Where necessary, inter-group comparisons will be made using t-tests or Fisher's exact test. Adverse events will be coded according to the Medical Dictionary for Regulatory Activities (MedDRA).

1. Other Analyses

**Pharmacokinetic (PK) Analysis**

Concentration-time (c-t) data analysis: Individual and average c-t curves, and semi-logarithmic c-t curves will be plotted using the PK Parameter Set (PKPS). Concentration data will be statistically described according to the planned sampling time points, listing the mean, standard deviation, median, maximum, minimum, and coefficient of variation of drug concentrations at each time point.

PK parameter calculations will be completed using Phoenix WinNonlin version 8.1 or higher, using the PKPS. Non-compartmental models will be used to calculate pharmacokinetic parameters for each subject, and the arithmetic mean, standard deviation, coefficient of variation, median, maximum, minimum, geometric mean, and geometric mean coefficient of variation for each parameter will be calculated. If a subject's AUC__%Extrap_ > 20%, AUC_0-∞_, t_1/2_, λ_z_, and AUC__%Extrap_ will not be included in descriptive statistical analysis.

**Immunogenicity (ADA) Analysis**

Antibody concentrations will be analyzed using descriptive statistical measures.

## 9.6 Number of Clinical Trial Cases per Disease and the Rationale for Determination

The trial protocol includes strict criteria for patient enrollment and exclusion, indicating that the current study is conducted solely for patients with a single indication. All patients mentioned belong to the same disease category and will not be further divided.

## 9.7 Statistical Procedures for All Data, Including Missing, Unused, or Erroneous Data (Including Dropouts and Withdrawals) and Unreasonable Data

For missing data, processing will be conducted according to the dataset classification. For primary efficacy data, since it is survival analysis data, imputation is not required. During the data management process, data in the database will be logically checked, and unreasonable data will be queried using a query form. Adjustments to unreasonable data will be made according to the written responses from the investigators until all unreasonable data are resolved, and the database can be locked.

During the data management process, data quality management will be conducted, and erroneous data will be queried using a query form. Corrections to erroneous data will be made according to the written responses from the investigators until all erroneous data are corrected, and the database can be locked.

## 9.8 Procedures for Reporting Deviations from the Original Statistical Plan

Any deviations from the original statistical plan will be explained in the final clinical trial summary report.

## 9.9 Exclusion of Special Information and the Rationale When Verifying Assumptions (If Applicable)

Not applicable.

# 10 Ethical Principles

## 10.1 Investigator's Responsibilities

Investigators are responsible for ensuring compliance with the Declaration of Helsinki and the Chinese Good Clinical Practice (GCP) guidelines, as well as relevant laws and regulations, in the execution of this trial protocol.

## 10.2 Ethical Committee Approval

Prior to the commencement of the trial, investigators should provide the ethical committee with copies of the following documents:

1. The clinical trial approval issued by the National Medical Products Administration;
2. The clinical trial protocol revised after discussion by the principal investigator;
3. A sample of the informed consent form;
4. Case report forms;
5. The investigator's brochure;
6. Investigators' resumes;
7. Any other documents required by the ethical committee.

This trial may only begin after obtaining written approval from the ethical committee. Should the trial protocol require amendment during the course of the study, the revised protocol must be submitted again for record/approval by the responsible ethical committee before implementation. If significant new information is discovered regarding the trial medication, the relevant information must be submitted in writing for approval by the responsible ethical committee, and informed consent must be obtained from the subjects once more. Upon completion of the trial, the sponsor and investigators should notify the ethical committee of the trial's conclusion.

## 10.3 Informed Consent

Before subjects are enrolled in the trial, investigators must explain to them the purpose of the trial, methods, potential benefits, possible risks, and discomforts that may occur. Subjects should be informed that participation in the trial is voluntary and that they may withdraw at any time without affecting their treatment for the condition, and that their privacy will be protected.

Subjects or their guardians should be given sufficient time to read the informed consent form and raise questions. Prior to enrollment, subjects or their guardians must sign the informed consent form, and subjects should retain a copy of the signed informed consent document.

## 10.4 Confidentiality of Subject Information

Only information necessary for the study of the drug's efficacy and safety will be collected in this trial. The collection and use of this data will adhere to relevant laws and regulations for the protection of privacy rights.

# 11 Study Management

## 11.1 Protocol Amendments

Neither the investigators nor the sponsor may alter the trial protocol without the other party's consent. All protocol amendments will be issued by the sponsor. Significant amendments typically require submission to health regulatory authorities and the leading unit for their approval and endorsement. In such cases, the protocol may only be executed with approval or consent.

Significant protocol modifications, including administrative changes, will be archived by the sponsor at the research centers. They will only be submitted to the appropriate health regulatory authorities when required by relevant regulations.

Any amendments that might affect a subject's willingness to participate in this clinical study must be consented to by the subject before execution.

## 11.2 Registration Documents

#### 11.2.1 Registration Approval/Notification

This clinical trial protocol will be registered online with the National Medical Products Administration.

#### 11.2.2 Documents Required Before the Trial

Before the trial commences, the sponsor should obtain the following documents:

1. Clinical trial approval from the National Medical Products Administration;
2. The clinical trial protocol approved by the principal investigator;
3. Ethical committee approval;
4. Resumes of the investigators and each research personnel involved in the trial;
5. Signed clinical trial agreements;
6. Documents certifying laboratory qualifications and normal values of laboratory test results;
7. Publicly available financial information of the research institution (e.g., financial accounts).

## 11.3 Trial-Related Items

The following items will be provided to the investigators at each clinical trial unit by the clinical monitor:

1. The clinical trial protocol approved by the principal investigator;
2. The informed consent form approved by the ethical committee;
3. Case report forms;
4. Investigator's brochure;
5. Trial medication;
6. Other trial supplies.

Upon receipt of the above items, the investigator must sign and date the receipt form.

## 11.4 Case Report Form Management

Data recorded during the study must be entered into the eCRF, and data handling, evaluation, and storage should be in accordance with data confidentiality regulations. The investigator or their designated personnel will be responsible for entering data into the eCRF provided by the sponsor. Ensuring the accuracy of the data entered into the eCRF is the responsibility of the investigator. eCRF management will be conducted by the sponsor and their designated responsible party. The data will be entered into a confirmed database. The sponsor's data management department or designated CRO will handle the data under the supervision of the sponsor, following specified data management procedures. The database will be closed once the quality assurance procedures are completed. A PDF file of the eCRF will be provided to the investigator upon completion of the study.

## 11.5 Original Data and Subject Documentation

For each subject enrolled in the study, the investigator must maintain every written or electronic subject file (medical records, source documents). These documents must include demographic or medical information of the subject, as complete as possible. The file should particularly include the following information:

- Subject's full name
- Date of birth
- Gender
- Height
- Weight
- Medical history and concomitant diseases
- Previous and concomitant treatments (including changes during treatment)
- Treatment number
- Date of subject's enrollment in the study (e.g., date of informed consent signature)
- Dates of subject's visits to the research center
- Any medical examinations and clinical findings defined in the study protocol
- All adverse events experienced by the subject
- Date of subject's discontinuation of the study
- If applicable, the date and reason for the subject's early withdrawal from the study or discontinuation of the study medication

These subject documents will allow for the identification of each subject.

Additionally, any other documents containing original data must be archived. This includes, but is not limited to, CT and MRI scan images, X-ray films, ECG records, and laboratory test result lists. These should be labeled with the subject's number and the date of the examination. Where possible, this information should be printed by the instrument used for assessment or measurement; information that cannot be printed automatically may be entered manually. The investigator should archive these records with medical evaluations, signed, and dated.

## 11.6 Research Center Documentation and Archiving

At the initiation of the study, a research center document will be provided to the investigator. This document will include all necessary documents for conducting the clinical trial and will be continuously updated throughout the study. This document will be available for inspection by auditors, sponsor's monitoring, and regulatory inspections, and must be properly stored for at least 5 years after the study's completion. Documents that must be archived also include: subject identification forms and signed informed consent forms. If the research center can no longer continue to preserve the center documents, the investigator must notify the sponsor.

All original subject documents (medical records) must be preserved at the research center (hospital, research institute, or clinical unit) for as long as possible, in accordance with regulations or GCP guidelines. Under no circumstances should the investigator ensure the destruction of medical records without the sponsor's written approval.

## 11.7 Use and Publication of Information

All information and data regarding this trial are confidential and proprietary to the sponsor. They cannot be used for other purposes without the sponsor's written consent.

Investigators should provide all data obtained from the trial to the sponsor. The sponsor has the right to publish this information and data without the investigator's consent and owns the copyright and reproduction rights for works and publications based on the content of this trial. If investigators wish to publish information from the trial, they should provide the manuscript to the sponsor for review and obtain written consent 60 days before publication. This is not to restrict or prevent the normal publication of research results but to protect legitimate trade secrets.

# 12 References

[1] Torre L A, Bray F, Siegel R L, *et al*. Global cancer statistics, 2012[J]. CA: a cancer journal for clinicians, 2015, 65(2): 87-108.

[2] Chen W, Zheng R, Baade P D, *et al*. Cancer statistics in China, 2015[J]. CA: a cancer journal for clinicians, 2016, 66(2): 115-132.

[3] Siegel R L, Miller K D, Jemal A. Cancer statistics, 2015[J]. CA: a cancer journal for clinicians, 2015, 65(1): 5-29.

[4] Harari P M, Allen G W, Bonner J A. Biology of interactions: antiepidermal growth factor receptor agents[J]. J Clin Oncol, 2007, 25(26):4057-4065.

[5] Mitsudomi T, Yatabe Y. Epidermal growth factor receptor in relation to tumor development: EGFR gene and cancer[J]. FEBS journal, 2010, 277(2): 301-308.

[6] Aaronson S. Growth Factor and Receptor Tyrosine Kinases[J]. Annual Review of Biochemistry, 1988, 57(4):443-478.

[7] Saleh M N, Raisch K P, Stackhouse M A, *et al*. Combined modality therapy of A431 human epidermoid cancer using anti-EGFr antibody C225 and radiation.[J]. Cancer Biotherapy & Radiopharmaceuticals, 1999, 14(6):451-463.

[8] Chung C H, Ely K, Mcgavran L, *et al*. Increased epidermal growth factor receptor gene copy number is associated with poor prognosis in head and neck squamous cell carcinomas[J]. J Clin Oncol, 2006, 24(25):4170-4176.

[9] Liang K, Ang K K, Milas L, *et al*. The epidermal growth factor receptor mediates radioresistance[J]. Int J Radiat Oncol Biol Phys, 2003, 57(1):246-254.

[10] Huang S M, Harari P M. Epidermal growth factor receptor inhibition in cancer therapy: biology, rationale and preliminary clinical results[J]. Invest New Drugs, 1999, 17(3):259-269.

[11] Kimura H, Sakai K, Arao T, *et al.* Antibody‐dependent cellular cytotoxicity of cetuximab against tumor cells with wild‐type or mutant epidermal growth factor receptor[J]. Cancer Science, 2007, 98(8): 1275-1280.

[12] Lenz H-J, Mayer RJ, Mirtsching B, *et al.* Consistent response to treatment with cetuximab monotherapy in patients with metastatic colorectal cancer[J]. J Clin Oncol, 2005, 23: 16S (Abstr 3536).

[13] Astrid Lievre, Bachet J B, Delphine LC, *et al.* KRAS mutation status is predictive of response to cetuximab therapy in colorectal cancer[J]. Cancer Res, 2006, 66:3992-3995.

[14] Schirripa M, Cremolini C, Loupakis F, *et al*. Role of NRAS mutations as prognostic and predictive markers in metastatic colorectal cancer[J]. Int J Cancer, 2015, 136(1):83-90.

[15] Van C E, Köhne C H, Hitre E, *et al*. Cetuximab and chemotherapy as initial treatment for metastatic colorectal cancer[J]. New England Journal of Medicine, 2009, 360(14):1408-1417.

[16] De R W, Piessevaux H, De S J, *et al*. KRAS wild-type state predicts survival and is associated to early radiological response in metastatic colorectal cancer treated with cetuximab[J]. Annals of Oncology, 2008, 19(3):508-515.

[17] Lièvre A, Bachet J B, Corre D L, *et al.* KRAS mutation status is predictive of response to cetuximab therapy in colorectal cancer[J]. Cancer Research, 2006, 66(8):3992-3995.

[18] Van C E, Köhne C H, Láng I, *et al*. Cetuximab plus irinotecan, fluorouracil, and leucovorin as first-line treatment for metastatic colorectal cancer: updated analysis of overall survival according to tumor KRAS and BRAF mutation status[J]. Journal of Clinical Oncology, 2011, 29(29):2011-2019.

[19] Van C E, Köhne C H, Lenz H j, *et al*. Fluorouracil, Leucovorin, and Irinotecan Plus Cetuximab Treatment and RAS Mutations in Colorectal Cancer[J]. Journal of Clinical Oncology Official Journal of the American Society of Clinical Oncology, 2015, 33(7):692-700.

[20] Van C E, Köhne C H, Láng I, *et al*. Cetuximab plus irinotecan, fluorouracil, and leucovorin as first-line treatment for metastatic colorectal cancer: updated analysis of overall survival according to tumor KRAS and BRAF mutation status[J]. Journal of Clinical Oncology, 2011, 29(15):2011-2019.

[21] Laurentpuig P, Cayre A, Manceau G, *et al.* Analysis of PTEN, BRAF, and EGFR status in determining benefit from cetuximab therapy in wild-type KRAS metastatic colon cancer[J]. Journal of Clinical Oncology Official Journal of the American Society of Clinical Oncology, 2009, 27(35):5924-5930.

[22] Therkildsen C, Bergmann T K, Henrichsen-Schnack T, *et al*. The predictive value of KRAS, NRAS, BRAF, PIK3CA and PTEN for anti-EGFR treatment in metastatic colorectal cancer: A systematic review and meta-analysis[J]. Acta Radiologica: Oncology, Radiation, Physics, Biology, 2014, 53(7):852-864.

[23] Vecchione L. Optimization of Anti-EGFR Treatment of Advanced Colorectal Cancer[J]. Current Colorectal Cancer Reports, 2014, 10(3):263-271.

[24] Baselga J, Pfister D, Cooper M R, *et al.* Phase I studies of anti-epidermal growth factor receptor chimeric antibody C225 alone and in combination with cisplatin.[J]. Journal of Clinical Oncology, 2000, 18(18):904-914.

[25] Chong W, He X, Bo Z, *et al*. Phase 1 study of anti-epidermal growth factor receptor monoclonal antibody in patients with solid tumors[J]. Mabs, 2011, 3(1):67-75.

[26] Mosteller R D. Simplified calculation of body surface area[J]. N Engl J Med, 1987, 317:1098.

[27] Bokemeyer C, Bondarenko I, Makhson A, *et al.* Fluorouracil, Leucovorin, and Oxaliplatin With and Without Cetuximab in the First-Line Treatment of Metastatic Colorectal Cancer[J]. J Clin Oncol, 2009, 27:663-671.

[28] Coppa G F. Surgical resection for colorectal hepatic metastases[J]. Bull NY Acad Med, 1990, 66(3):211.

[29] Kavolius J, Fong Y, Blumgart L H. Surgical resection of metastatic liver tumors[J]. Surg Oncol Clin North Am, 1996, 5(2):337.

# Appendix

# Appendix 1 Staging of Rectal and Colon Cancer (TNM Staging System)

The universally recognized standard for the classification of colorectal cancer, both domestically and internationally, is the 7th edition of the TNM staging system for colorectal cancer jointly established by the American Joint Committee on Cancer (AJCC) and the International Union for Cancer Control (UICC), revised in 2010.

**Primary Tumor (T)**

Tx: Primary tumor cannot be assessed

T0: No evidence of primary tumor

Tis: Carcinoma in situ: confined to the epithelium or invading the lamina propria mucosae

T1: Tumor invades the submucosa

T2: Tumor invades the muscularis propria

T3: Tumor penetrates through the muscularis propria to the subserosal connective tissue, or invades pericolic or perirectal tissues without serosal covering

T4a: Tumor perforates the visceral peritoneum

T4b: Tumor directly invades or is adherent to other organs or structures

**Regional Lymph Nodes (N)**

Nx: Regional lymph nodes cannot be assessed

N0: No regional lymph node metastasis

N1: Metastasis in 1 to 3 regional lymph nodes

N1a: Metastasis in 1 regional lymph node

N1b: Metastasis in 2 to 3 regional lymph nodes

N1c: Tumor deposit (TD) in subserosal, mesenteric, or non-peritonealized pericolic or perirectal tissues without regional lymph node metastasis

N2: Metastasis in more than 4 regional lymph nodes

N2a: Metastasis in 4 to 6 regional lymph nodes

N2b: Metastasis in 7 or more regional lymph nodes

**Distant Metastasis (M)**

M0: No distant metastasis

M1: Distant metastasis

M1a: Distant metastasis confined to one organ or site (such as liver, lung, ovary, non-regional lymph nodes)

M1b: Distant metastasis in more than one organ/site or peritoneal metastasis

**Anatomical Staging and Prognostic Categories**

| **Prognostic Categories** | **T** | **N** | **M** | **Dukes Staging** | **MAC Staging** |
| --- | --- | --- | --- | --- | --- |
| 0 | Tis | N_0_ | M_0_ | - | - |
| Ⅰ | T_1_ | N_0_ | M_0_ | A | A |
|  | T_2_ | N_0_ | M_0_ | A | B_1_ |
| ⅡA | T_3_ | N_0_ | M_0_ | B | B_2_ |
| ⅡB | T_4a_ | N_0_ | M_0_ | B | B_2_ |
| ⅡC | T_4b_ | N_0_ | M_0_ | B | B_3_ |
| ⅢA | T_1_～T_2_ | N_1_/N_1c_ | M_0_ | C | C_1_ |
|  | T_1_ | N_2a_ | M_0_ | C | C_1_ |
| ⅢB | T_3_～T_4a_ | N_1_/N_1c_ | M_0_ | C | C_2_ |
|  | T_2_～T_3_ | N_2a_ | M_0_ | C | C_1_/C_2_ |
|  | T_1_～T_2_ | N_2b_ | M_0_ | C | C_1_ |
| ⅢC | T_4a_ | N_2a_ | M_0_ | C | C_2_ |
|  | T_3_～T_4a_ | N_2b_ | M_0_ | C | C_2_ |
|  | T_4b_ | N_1_～N_2_ | M_0_ | C | C_3_ |
| ⅣA | Any T | Any N | M_1a_ | - | - |
| ⅣB | Any T | Any N | M_1b_ | - | - |

# Appendix 2 The Common Terminology Criteria for Adverse Events (CTCAE) Version 4.03 by the National Cancer Institute (NCI)

| **Term** | **Grade 1** | **Grade 2** | **Grade 3** | **Grade 4** | **Grade 5** |
| --- | --- | --- | --- | --- | --- |
| Nausea | Loss of appetite without alteration in eating habits | Oral intake decreased without significant weight loss, dehydration or malnutrition | Inadequate oral caloric or fluid intake; tube feeding, TPN, or hospitalization indicated | - | - |
| Oral pain | Mild pain | Moderate pain; limiting instrumental ADL | Severe pain; limiting self care ADL | - | - |
| Stomach pain | Mild pain | Moderate pain; limiting instrumental ADL | Severe pain; limiting self care ADL | - | - |
| Vomiting | 1 - 2 episodes (separated by 5 minutes) in 24 hrs | 3 - 5 episodes (separated by 5 minutes) in 24 hrs | >=6 episodes (separated by 5 minutes) in 24 hrs; tube feeding, TPN or hospitalization indicated | Life-threatening consequences; urgent intervention indicated | Death |
| Edema limbs | 5 - 10% inter-limb discrepancy in volume or circumference at point of greatest visible difference; swelling or obscuration of anatomic architecture on close inspection | >10 - 30% inter-limb discrepancy in volume or circumference at point of greatest visible difference; readily apparent obscuration of anatomic architecture; obliteration of skin folds; readily apparent deviation from normal anatomic contour; limiting instrumental ADL | >30% inter-limb discrepancy in volume; gross deviation from normal anatomic contour; limiting self care ADL | - | - |
| Fatigue | Fatigue relieved by rest | Fatigue not relieved by rest; limiting instrumental ADL | Fatigue not relieved by rest, limiting self care ADL | - | - |
| Fever | 38.0 - 39.0 degrees C (100.4 - 102.2 degrees F) | >39.0 - 40.0 degrees C (102.3 - 104.0 degrees F) | >40.0 degrees C (>104.0 degrees F) for <=24 hrs | >40.0 degrees C (>104.0 degrees F) for >24 hrs | Death |
| Gait disturbance | Mild change in gait (e.g., wide-based, limping or hobbling) | Moderate change in gait (e.g., wide-based, limping or hobbling); assistive device indicated; limiting instrumental ADL | Disabling; limiting self care ADL | - | - |
| Injection site reaction | Tenderness with or without associated symptoms (e.g., warmth, erythema, itching) | Pain; lipodystrophy; edema; phlebitis | Ulceration or necrosis; severe tissue damage; operative intervention indicated | Life-threatening consequences; urgent intervention indicated | Death |
| Localized edema | Localized to dependent areas, no disability or functional impairment | Moderate localized edema and intervention indicated; limiting instrumental ADL | Severe localized edema and intervention indicated; limiting self care ADL | - | - |
| Pain | Mild pain | Moderate pain; limiting instrumental ADL | Severe pain; limiting self care ADL | - | - |
| Lung infection | - | Moderate symptoms; oral intervention indicated (e.g., antibiotic, antifungal, antiviral) | IV antibiotic, antifungal, or antiviral intervention indicated; radiologic, endoscopic, or operative intervention indicated | Life-threatening consequences; urgent intervention indicated | Death |
| Skin infection | Localized, local intervention indicated | Oral intervention indicated (e.g., antibiotic, antifungal, antiviral) | IV antibiotic, antifungal, or antiviral intervention indicated; radiologic or operative intervention indicated | Life-threatening consequences; urgent intervention indicated | Death |
| Alanine aminotransferase increased | >ULN - 3.0 x ULN | >3.0 - 5.0 x ULN | >5.0 - 20.0 x ULN | >20.0 x ULN | - |
| Alkaline phosphatase increased | >ULN - 2.5 x ULN | >2.5 - 5.0 x ULN | >5.0 - 20.0 x ULN | >20.0 x ULN | - |
| Aspartate aminotransferase increased | >ULN - 3.0 x ULN | >3.0 - 5.0 x ULN | >5.0 - 20.0 x ULN | >20.0 x ULN | - |
| Blood bilirubin increased | >ULN - 1.5 x ULN | >1.5 - 3.0 x ULN | >3.0 - 10.0 x ULN | >10.0 x ULN | - |
| Creatinine increased | >1 - 1.5 x baseline; >ULN - 1.5 x ULN | >1.5 - 3.0 x baseline; >1.5 - 3.0 x ULN | >3.0 baseline; >3.0 - 6.0 x ULN | >6.0 x ULN | - |
| Hemoglobin increased | Increase in >0 - 2 gm/dL above ULN or above baseline if baseline is above ULN | Increase in >2 - 4 gm/dL above ULN or above baseline if baseline is above ULN | Increase in >4 gm/dL above ULN or above baseline if baseline is above ULN | - | - |
| Neutrophil count decreased | <LLN - 1500/mm3; <LLN - 1.5 x 10e9 /L | <1500 - 1000/mm3; <1.5 - 1.0 x 10e9 /L | <1000 - 500/mm3; <1.0 - 0.5 x 10e9 /L | <500/mm3; <0.5 x 10e9 /L | - |
| Platelet count decreased | <LLN - 75,000/mm3; <LLN - 75.0 x 10e9 /L | <75,000 - 50,000/mm3; <75.0 - 50.0 x 10e9 /L | <50,000 - 25,000/mm3; <50.0 - 25.0 x 10e9 /L | <25,000/mm3; <25.0 x 10e9 /L | - |
| White blood cell decreased | <LLN - 3000/mm3; <LLN - 3.0 x 10e9 /L | <3000 - 2000/mm3; <3.0 - 2.0 x 10e9 /L | <2000 - 1000/mm3; <2.0 - 1.0 x 10e9 /L | <1000/mm3; <1.0 x 10e9 /L | - |
| Hypokalemia | <LLN - 3.0 mmol/L | <LLN - 3.0 mmol/L; symptomatic; intervention indicated | <3.0 - 2.5 mmol/L; hospitalization indicated | <2.5 mmol/L; life-threatening consequences | Death |
| Hypomagnesemia | <LLN - 1.2 mg/dL; <LLN - 0.5 mmol/L | <1.2 - 0.9 mg/dL; <0.5 - 0.4 mmol/L | <0.9 - 0.7 mg/dL; <0.4 - 0.3 mmol/L | <0.7 mg/dL; <0.3 mmol/L; life-threatening consequences | Death |

# Appendix 3 Performance Status Scale (Eastern Cooperative Oncology Group)

| **Activity Status** | **Description** |
| --- | --- |
| 0 | Asymptomatic, fully active, and able to carry out all pre-illness activities without restriction |
| 1 | Symptomatic, fully ambulatory, but limited to heavy activities，able to perform light or sedentary work, such as light housework or office work |
| 2 | Symptomatic, ambulatory, self-care capable, but unable to perform any strenuous physical activity, more than 50% of waking hours is free of bed rest: that is, daytime bed rest time is less than 50% |
| 3 | Symptomatic, with limited self-care capability, more than 50% of waking hours in bed or chair, but not bedbound |
| 4 | Completely disabled, unable to perform any self-care, bedbound |
| 5 | Death |

# Appendix 4 New response evaluation criteria in solid tumors: Revised RECIST guideline (version 1.1)

**I. Measurable Lesions**

Measurable lesions include:

- Tumors with a longest diameter of ≥10 mm or lymph nodes with a shortest diameter of ≥15 mm on 5 mm thin-section CT scans
- Tumors with a longest diameter of ≥20 mm on well-contrasted chest X-ray films
- Superficial lesions, such as skin nodules measurable with a caliper, ≥10 mm in size can also be considered measurable lesions.

**II. Non-measurable Lesions**

Non-measurable lesions include:

- Tumors with a longest diameter of <10 mm
- Pathologically positive lymph nodes with a shortest diameter of ≥10 mm but <15 mm
- Leptomeningeal lesions
- Ascites
- Pleural or pericardial effusions
- Inflammatory breast cancer
- Carcinomatous lymphangitis of the skin or lung
- Abdominal masses or lumps detected on physical examination but not reproducible by imaging techniques

**III. Selection of Target Lesions**

When there is more than one measurable lesion, a maximum of 2 per organ and a total of up to 5 throughout the body should be chosen as target lesions.

The principles for selecting target lesions are:

- Typically having the largest diameter
- Each involved organ should be selected
- Having reproducibility on imaging

When the largest lesion in an organ is not reproducible on imaging, the next largest lesion should be chosen.

Only pathologically positive lymph nodes with a shortest diameter of ≥15 mm are counted as target lesions.

**IV. Lesion Assessment Methods**

The same assessment method should be used for the evaluation of the same lesion at baseline and during treatment follow-up. Based on the objectivity and retrospective nature of imaging, efficacy assessment should be performed through imaging rather than physical examination, unless the lesion cannot indeed be presented by imaging techniques but can be assessed by physical examination. For superficial lesions measurable with a caliper, their size should be clearly marked with a ruler in color photographs; imaging techniques should be the first choice for assessment if they can evaluate.

CT is currently the most commonly used method for efficacy assessment and a relatively reproducible anatomical imaging technique in RECIST. When performing scans of the chest, abdomen, and pelvis, all areas of interest should be covered. Variations in the thickness of CT scans can affect the measurement of lesions and the detection of new lesions; the standard should be 5 mm thin-section CT, as it can display all measurable lesions of 10 mm. If the CT scan slice thickness is >5 mm, the longest diameter of measurable lesions should be >10 mm. Lesions that were not scanned at baseline and then appear are considered new lesions, indicating disease progression; therefore, the baseline CT scan range should include the primary lesion, all common metastatic sites, and all areas where signs and symptoms appear.

In some cases, MRI may be used for whole-body scanning. MRI has good contrast, stereo, and temporal resolution, but image acquisition includes various variables that greatly affect image quality, lesion visibility, and measurement. The same lesion should use the same brand and model of the machine and set the same variable parameters each time it is scanned with MRI.

Chest X-rays can only clearly present lesions surrounded by lung parenchyma and are not suitable for assessing other parts of the tumor.

Ultrasonography, due to its operator dependence, is inevitably subjective, and it cannot guarantee that the same techniques and measurement methods will be used each time, and it cannot be reproduced during later independent assessments, so it cannot be used to measure the size of lesions. If new lesions are detected by ultrasound, they are recommended to be confirmed by CT or MRI.

**V. Efficacy Assessment Criteria**

- Complete Response (CR): All target lesions disappear. The shortest diameter of all pathologically positive lymph nodes (whether target or non-target lesions) must be reduced to <10 mm.
- Partial Response (PR): Compared with the sum of the longest diameter (LD) of the baseline lesions, the sum of the LD of target lesions decreases by at least 30%.
- Progression (PD): The sum of the LD of target lesions increases by at least 20% compared with the smallest sum of LD recorded since the start of treatment, and the absolute value of the increase in the sum of LD is at least 5 mm. The appearance of one or more new lesions is also considered PD.
- Stable Disease (SD): Compared with the smallest sum of LD recorded since the start of treatment, the sum of LD of lesions has not reached the reduction amount of partial remission, nor has it reached the increase amount of tumor progression.

**VI. Confirmation of Efficacy**

For clinical studies with ORR as the main endpoint, efficacy must be confirmed. However, randomized controlled phase III clinical studies with Overall Survival (OS) as the main endpoint do not require efficacy confirmation.

# Appendix 5 EORTC Quality of Life Questionnaire - Core Questionnaire (QLQ-C30)

We are very interested in understanding your health status and quality of life. Please answer the following questions by circling the response that best applies to you. There are no "right" or "wrong" answers to these questions. The information you provide will be kept in strict confidence.

|  | **None** | **Mild** | **Moderate** | **Severe** |
| --- | --- | --- | --- | --- |
| 1. 1. Do you have difficulty when performing physically demanding tasks, such as carrying heavy shopping bags or suitcases? | 1 | 2 | 3 | 4 |
| 1. 2. Do you have difficulty with long-distance walking? | 1 | 2 | 3 | 4 |
| 1. 3. Do you have difficulty with short walks outside? | 1 | 2 | 3 | 4 |
| 1. 4. Do you spend most of the day in bed or sitting in a chair? | 1 | 2 | 3 | 4 |
| 1. 5. Do you require assistance with eating, dressing, bathing, or using the toilet? | 1 | 2 | 3 | 4 |
| (Within the past week) | | | | |
| 1. 6. Are your work or daily activities limited? | 1 | 2 | 3 | 4 |
| 1. 7. Do you feel restricted in your favorite or other leisure activities? | 1 | 2 | 3 | 4 |
| 1. 8. Have you experienced shortness of breath? | 1 | 2 | 3 | 4 |
| 1. 9. Do you have pain? | 1 | 2 | 3 | 4 |
| 1. 10. Have you needed to rest? | 1 | 2 | 3 | 4 |
| 1. 11. Do you have difficulty sleeping? | 1 | 2 | 3 | 4 |
| 1. 12. Have you felt weak? | 1 | 2 | 3 | 4 |
| 1. 13. Have you lost your appetite? | 1 | 2 | 3 | 4 |
| 1. 14. Have you felt nauseous? | 1 | 2 | 3 | 4 |
| 1. 15. Have you vomited? | 1 | 2 | 3 | 4 |
| 1. 16. Have you had constipation? | 1 | 2 | 3 | 4 |
| 1. 17. Have you had diarrhea? | 1 | 2 | 3 | 4 |
| 1. 18. Do you feel fatigued? | 1 | 2 | 3 | 4 |
| 1. 19. Does pain interfere with your daily activities? | 1 | 2 | 3 | 4 |
| 1. 20. Do you have difficulty concentrating on tasks, such as reading the newspaper or watching television? | 1 | 2 | 3 | 4 |
| 1. 21. Have you felt nervous? | 1 | 2 | 3 | 4 |
| 1. 22. Do you have worries? | 1 | 2 | 3 | 4 |
| 1. 23. Do you feel irritable? | 1 | 2 | 3 | 4 |
| 1. 24. Do you feel depressed? | 1 | 2 | 3 | 4 |
| 1. 25. Do you have difficulty remembering things? | 1 | 2 | 3 | 4 |
| 1. 26. Has your physical condition or treatment interfered with your family life? | 1 | 2 | 3 | 4 |
| 1. 27. Has your physical condition or treatment interfered with your social activities? | 1 | 2 | 3 | 4 |
| 1. 28. Has your physical condition or treatment caused you financial difficulties? | 1 | 2 | 3 | 4 |
| 1. (For the following questions, please circle the number from 1 to 7 that best applies to you) | | | | |
| 1. How would you rate your overall health during the past week? | 1. 1 2 3 4 5 6 7 2. Extremely Poor Excellent | | | |
| 1. How would you rate your overall quality of life during the past week? | 1. 1 2 3 4 5 6 7   Extremely Poor Excellent | | | |
