# Supplementary material for: First-line treatment of anti-EGFR monoclonal antibody cetuximab β plus FOLFIRI versus FOLFIRI alone in Chinese patients with RAS/BRAF wild-type metastatic colorectal cancer: a randomized, phase 3 trial
Source: Signal Transduct Target Ther. 2025 May 7;10:147. doi: 10.1038/s41392-025-02229-4 (PMC12056184; doi:10.1038/s41392-025-02229-4)
Supplement: Supplementary file 3 — Statistical Analysis Plan [file 41392_2025_2229_MOESM3_ESM.docx]

|  |
| --- |
| **A Phase III Open-Label, Randomized Controlled, Multicenter, Prospective Clinical Study of Recombinant Anti-EGFR Human-Mouse Chimeric Monoclonal Antibody Injection (CMAB009) Combined with FOLFIRI Chemotherapy Regimen versus FOLFIRI Chemotherapy Alone as First-Line Treatment for RAS/BRAF Wild-Type Metastatic Colorectal Cancer** |
| **Statistical Analysis Plan**   \| **Version.** \| **V1.0** \| \| --- \| --- \| \| **Version Date.** \| **2022-08-07** \|  \| **Lead Units** \| **Cancer Hospital, Chinese Academy of Medical Sciences**  **Tianjin Medical University Cancer Institute** \| \| --- \| --- \| \| **Sponsor** \| **Taizhou Mabtech Pharmaceutical Co., Ltd.** \| \| **Statistical Unit** \| **Department of Medical Statistics, Peking University First Hospital** \| |
|  |

**Version Update Record**

| **releases** | **dates** | **Updates** |
| --- | --- | --- |
|  |  |  |
|  |  |  |

**Table of Content**

[1. Summary of the Statistical Analysis Plan 4](#_Toc178600336)

[2. Overview of the Study Design and Protocol 10](#_Toc178600337)

[2.1 Study Title 10](#_Toc178600338)

[2.2 Study Objective 10](#_Toc178600339)

[2.3 Study Design 10](#_Toc178600340)

[2.4 Estimand 10](#_Toc178600341)

[2.4.1 Population 10](#_Toc178600342)

[2.4.2 Treatment 10](#_Toc178600343)

[2.4.3 Variable 11](#_Toc178600344)

[2.4.4 Intercurrent Events and Handling Strategies 11](#_Toc178600345)

[2.4.5 Population-level Summary 12](#_Toc178600346)

[2.5 Sample Size 12](#_Toc178600347)

[2.6 Research Interventions 12](#_Toc178600348)

[2.7 Randomization 14](#_Toc178600349)

[2.8 Blind Design and Bias Control 14](#_Toc178600350)

[2.9 Interim 15](#_Toc178600351)

[2.10 Follow Up 15](#_Toc178600352)

[2.11 Study Flowchart 16](#_Toc178600353)

[3. Evaluation Indicators 19](#_Toc178600354)

[3.1 Efficacy Indicators 19](#_Toc178600355)

[3.1.1 Primary Efficacy Indicators. 19](#_Toc178600356)

[3.1.2 Secondary Efficacy Indicators 19](#_Toc178600357)

[3.2 Safety Evaluation Indicators 22](#_Toc178600358)

[3.3 Other Indicators 22](#_Toc178600359)

[4. Statistical Analysis 23](#_Toc178600360)

[4.1 Analysis Datasets 23](#_Toc178600361)

[4.2 General Principles of Statistics 24](#_Toc178600362)

[4.2.1 Software and Data Transfer 24](#_Toc178600363)

[4.2.2 Reporting of Results 24](#_Toc178600364)

[4.2.3 Rules for Handling Missing Data 25](#_Toc178600365)

[4.2.4 Rules for Handling Relevant Outcomes and Dates During Survival Aanalysis 27](#_Toc178600366)

[4.2.5 Baseline Definitions 28](#_Toc178600367)

[4.2.6 Data Derivation and Conversion 28](#_Toc178600368)

[4.3 Trial Completion and Subject Distribution 28](#_Toc178600369)

[4.4 Protocol Deviations 28](#_Toc178600370)

[4.5 Treatment Compliance 29](#_Toc178600371)

[4.6 Concomitant Medication and Concomitant Treatment 29](#_Toc178600372)

[4.7 Demographic and Baseline Characteristics 30](#_Toc178600373)

[4.8 Statistical Analysis of Primary Estimand 30](#_Toc178600374)

[4.8.1 Analysis Sets 30](#_Toc178600375)

[4.8.2 Main Statistical Analysis of Primary Estimand 30](#_Toc178600376)

[4.8.3 Sensitivity Analysis of the Primary Estimand 31](#_Toc178600377)

[4.8.4 Stratified Analysis of the Primary Estimand 31](#_Toc178600378)

[4.8.5 Subgroup Analysis of the Primary Estimand 32](#_Toc178600379)

[4.9 Secondary Estimand 32](#_Toc178600380)

[4.10 Safety Analysis 35](#_Toc178600381)

[4.10.1 Drug Exposure 35](#_Toc178600382)

[4.10.2 Adverse Event 35](#_Toc178600383)

[4.10.3 Vital Signs, Body Weight and ECOG Scores 38](#_Toc178600384)

[4.10.4 Physical Examination 39](#_Toc178600385)

[4.10.5 Laboratory Tests 39](#_Toc178600386)

[4.10.6 Electrocardiogram (ECG) 39](#_Toc178600387)

[4.11 Other Indicator Analysis 39](#_Toc178600388)

[4.11.1 Pharmacokinetic Evaluation 39](#_Toc178600389)

[4.11.2 Immunogenicity Evaluation 40](#_Toc178600390)

[5. Statistical Tables and Figures 40](#_Toc178600391)

[Attachments 41](#_Toc178600392)

# Summary of the Statistical Analysis Plan

| **SAP Purpose** | To evaluate the efficacy and safety of the combination of recombinant anti-EGFR human-mouse chimeric monoclonal antibody injection (CMAB009) with the FOLFIRI (irinotecan, 5-fluorouracil, leucovorin) chemotherapy regimen as a first-line treatment for patients with RAS/BRAF wild-type, metastatic colorectal cancer, compared to the FOLFIRI regimen alone. |
| --- | --- |
| **Clinical Research Protocol** | This SAP is based on the clinical research protocol version 2.4 (protocol number: 009mCRCIIIP, version date: 2022-02-24). |
| **Study Objectives** | (1) To evaluate the efficacy of the combination of recombinant anti-EGFR human-mouse chimeric monoclonal antibody injection (CMAB009) with the FOLFIRI (irinotecan, 5-fluorouracil, leucovorin) chemotherapy regimen as a first-line treatment for patients with RAS/BRAF wild-type, metastatic colorectal cancer, compared to the FOLFIRI regimen alone.  (2) To evaluate the safety of the combination of recombinant anti-EGFR human-mouse chimeric monoclonal antibody injection (CMAB009) with the FOLFIRI (irinotecan, 5-fluorouracil, leucovorin) chemotherapy regimen as a first-line treatment for patients with RAS/BRAF wild-type, metastatic colorectal cancer, compared to the FOLFIRI regimen alone. |
| **Endpoint Indicators** | **Primary Efficacy Endpoint**  Progression-Free Survival (PFS): The PFS duration (in months) is determined by a specially authorized Independent Radiology Review Committee (IRaC) through blinded review of imaging data. It is defined as the time from randomization to the first confirmed progression of disease by imaging or death from any cause within 90 days after the last tumor assessment or randomization, whichever is later (equivalent to 1.5 times the interval between two consecutive tumor assessments). If a subject has neither tumor progression nor death after the last tumor assessment in the study or within 90 days after randomization, they are censored at the date of the last tumor assessment.  Tumor progression and response are assessed according to RECIST criteria version 1.1. Tumor assessments are conducted every 8 weeks after randomization until the end of the study.  **Secondary Efficacy Endpoint**  (1) Objective Response Rate (ORR)  The objective response rate is calculated as the percentage of evaluable subjects with complete response (CR) and partial response (PR) (ORR = CR + PR).  (2) One-year Overall Survival Rate, Two-year Overall Survival Rate, and Overall Survival Time  One-year overall survival rate definition: The proportion of subjects alive at one year from randomization.  Two-year overall survival rate definition: The proportion of subjects alive at two years from randomization.  Overall survival time: Defined as the time from randomization to death (in months). For subjects still alive or lost to follow-up as of the data analysis cutoff date, survival is censored at the subject's last known alive time.  (3) Disease Control Rate (DCR)  Refers to the percentage of subjects with the best response of complete response (CR), partial response (PR), and stable disease (SD) according to RECIST 1.1 criteria (DCR = CR + PR + SD).  (4) Clinical Benefit Rate (CBR)  The proportion of subjects with the best tumor assessment of complete response (CR), partial response (PR), and stable disease (SD) that lasts for 24 weeks or more according to RECIST 1.1 criteria.  (5) Duration of Response (DOR)  For subjects with the best response of CR or PR according to RECIST 1.1 criteria, the time from the first occurrence of (CR or PR) to the first occurrence of disease progression or death. If a subject has not experienced an endpoint event, they are censored, with the censored date being the last imaging assessment date.  (6) Time to Response (TTR)  For subjects with the best response of CR or PR according to RECIST 1.1 criteria, the time from randomization to the first occurrence of response (CR or PR) according to RECIST 1.1.  (7) Quality of Life Assessment Indicators  Quality of life assessment (QOL) is conducted using the EORTC QLQ-C30 questionnaire (Appendix 5), with individual item categorical scores linearly transformed to a 0-100 scale. The questionnaire is self-reported, consisting of 30 items, and subjects are asked to complete it carefully. Assessments are conducted at baseline and subsequent visits every 8 weeks until withdrawal from the study and entry into the follow-up period.  (8) Resection Rate of Hepatic Metastasis  The resection rate of hepatic metastasis is calculated as the number of subjects achieving complete resection (R0 resection) divided by the total number of subjects.  **Safety Evaluation Indicators**   1. Drug exposure 2. Adverse events and incidence rates as defined using CTC AE version 4.03 3. Physical examination 4. Vital signs and weight: Vital signs include heart rate/pulse, respiration, temperature, blood pressure 5. ECOG 6. 12-lead electrocardiogram. 7. Laboratory parameters: Clinically significant changes in routine blood and urine tests, hepatic and renal function, and chest X-rays   **Other indicators**   1. Immunogenicity (ADA) 2. Pharmacokinetics 3. Concomitant taken at any time during the pre-existing and study periods or within 30 days of final study dosing. |
| **Study Design** | An open-label, randomized controlled, multicenter, prospective Phase III clinical trial |
| **Planning Analysis** | This study included only one final analysis, not a midterm analysis |
| **Analysis Datasets** | Full Analysis Set (FAS): According to the basic principle of Intention-To-Treat (ITT), the main efficacy indexes should include all randomized subjects, i.e., it is necessary to follow up the results of all randomized subjects completely, but it is often difficult to achieve in practice. Therefore, this trial was conducted using the full set. The full set refers to the set of subjects as close as possible to the principle of intentionality, which is derived from all randomized subjects with minimal and reasonable exclusion of certain cases.The FAS population will include all subjects randomized to the study and receiving at least 1 study treatment. Subjects after randomization will be, but if patients who are not wild-type for the RAS/BRAF gene are randomized, these patients will not be entered into the full set.  Per Protocol Set (PPS): A sample of cases that meets the requirements of the trial protocol, has good compliance, and has completed all the requirements of the trial, also known as "evaluable cases" or "eligible cases". A sample that is a subset of the full set. It is a subset of the full set, in which subjects are more adherent to the protocol, have completed a pre-determined minimum amount of treatment, have measurements on key variables, and have not committed major violations of the protocol. This dataset will be reviewed after the completion of the trial and finalized before the database is locked. Cases that violate the trial protocol, such as failure to achieve the prescribed treatment volume, absence of a primary variable, or use of an explicitly prohibited medication that affects the evaluation of the primary endpoint, will not be included, and justification will be provided for exclusion from this dataset.  The Per-Protocol population will not include all ITT populations that meet one or more of the following criteria:   - Randomization failure (e.g., receiving incorrect treatment) - No evidence of mCRC at baseline (e.g., no target lesions with the longest diameter ≥1cm at baseline) - Receiving study treatment for ≤8 weeks, excluding subjects who die or have PD within the first 8 weeks after starting treatment - Receiving unauthorized chemotherapy or other treatments during the study period - Significant protocol deviations affecting the evaluation of the primary endpoint occurred.   Safety Analysis Set (SAS): refers to all subjects who received at least one treatment after randomization, i.e., adverse events and adverse reactions should be reported whenever a subject was administered one or more drugs from the specified group, regardless of whether or not the subject was included in the compliant set.  The Response evaluable Set (RES) is a subset of the FAS and refers to subjects in the full set who had at least one tumor assessment at post-baseline follow-up.  PK Parameter Set (PKPS): Subjects who provided PK blood collection according to the protocol, the designated population of subjects in Group A. Subjects who received at least one study drug and had at least one pharmacokinetic parameter. Subjects who received at least one dose of study drug and had at least one pharmacokinetic parameter.PKPS was used for descriptive statistics of pharmacokinetic parameter data for subjects.  Immunogenicity (ADA) set: all patients enrolled and those who used the study at least once and had data from baseline and at least one post-baseline ADA evaluation.  Demographic and baseline characteristics will be performed using FAS; primary efficacy endpoint, secondary efficacy endpoints will be performed using both FAS and PPS; safety endpoints will be performed using SS; RES sets will also be utilized for objective response rate (ORR), disease control rate (DCR), clinical benefit rate (CBR), time to remission (DOR), and time to remission (TTR), which have tumor remission assessment metrics were performed; PKPS for pharmacokinetic parameters; ADA for immunogenicity metrics. |
| **Test Hypothesis** | Superiority testing hypothesis.  The PFS of the two groups would be stratified by center and ECOG score as covariates using Logrank test. The COX proportional risk model was used to estimate the risk ratio (HR) and its 95% confidence interval (CI) of the test group compared with the control group, assuming that the anti-EGFR monoclonal antibody + FOLFIRI combination treatment group was superior to the FOLFIRI monotherapy group, and the superiority cut-off value was HR=1, and the level of the test a was taken as one-sided 0.025. |
| **Main Analysis Content** | The content includes: subject screening, enrollment, withdrawal, dropout and exclusion, completion of the trial, demographic information and other baseline characteristics, evaluation of efficacy, laboratory results, vital signs, electrocardiogram, physical examination, occurrence of adverse events, etc. Safety evaluation, PK and immunogenicity.  SAS 9.4 or above version software will be used for statistics, qualitative indicators will be described by frequency, percentage or composition ratio; quantitative indicators will be described by mean, difference, median, quartile, maximum and minimum values. Comparisons between the two groups of relevant indicators will be made according to the type of indicators, and comparisons between groups of quantitative data will be made according to the distribution of data using group t-test (chi-square, normal distribution) or Wilcoxon rank-sum test, categorical data using chi-square test or exact probability method (if chi-square test is not applicable), and hierarchical data using Wilcoxon rank-sum test or CMH test.  For time-dependent variables such as PFS, DOR, OS, 1-year \2-year cumulative survival, Kaplan-Meier was used to estimate the survival function and plot the survival curves, and the Logrank test was performed between the two groups and the Cox proportional risk model was used as well as the covariates between the designated groups (center, ECOG scores) to calculate the risk ratios and their 95% confidence intervals between the treatment groups. For categorical indicators such as ORR, 1-year overall survival, 2-year overall survival, DCR, and CBR, logistic regression models were performed, in which covariates (centers, ECOG scores) were used for correction, and ratio ratios and their 95% confidence intervals were calculated between groups. For TTR, only subjects in remission were included and there was no censored data, it was performed according to the quantitative index, and the covariance model was used, in which the covariates (center, ECOG score) were used for correction, and the corrected mean, the difference of the corrected mean, and their 95% confidence intervals were calculated for the two groups. Life scores were performed according to quantitative indicators, and comparisons between the two groups were performed using the paired t-test (chi-square, normal distribution) or Wilcoxon rank-sum test. Proportion of subjects who underwent radical surgery for liver metastases and the rate of radical resection of liver metastases: rates were calculated separately for the two groups, while comparisons between groups were made using the chi-square test or Fisher's exact probability method. |

# Overview of the Study Design and Protocol

This is an open, randomized, controlled, multicenter, phase III clinical study evaluating the combination of anti-EGFR monoclonal antibody + FOLFIRI (group A) versus FOLFIRI alone (group B) for the first-line treatment of metastatic colorectal cancer with wild-type RAS/BRAF. The relevant elements of the statistical plan were developed based on the following study protocol: Clinical Research Protocol Version 2.4 (Protocol Number: 009mCRC IIIP, Version Date: 2022-02-24).

## Study Title

A phase III open-label, randomized controlled, multicenter, prospective clinical study of recombinant anti-EGFR human-mouse chimeric monoclonal antibody injection (CMAB009) combined with FOLFIRI chemotherapy regimen versus FOLFIRI chemotherapy alone as first-line treatment for RAS/BRAF wild-type metastatic colorectal cancer

## Study Objective

To evaluate the efficacy and safety of the combination of recombinant anti-EGFR human-mouse chimeric monoclonal antibody injection (CMAB009) with the FOLFIRI (irinotecan, 5-fluorouracil, leucovorin) chemotherapy regimen as a first-line treatment for patients with RAS/BRAF wild-type, metastatic colorectal cancer, compared to the FOLFIRI regimen alone

## Study Design

An open-label, randomized controlled, multicenter, prospective Phase III clinical trial

## Estimand

### Population

First-line treatment of patients with RAS/BRAF gene wild-type, metastatic colorectal cancer.

Definition of the first-line treatment population: subjects with a first metastatic lesion (not amenable to radical resection) who have not received chemotherapy or have received prior adjuvant or neoadjuvant chemotherapy for CRC, and whose chemotherapy was completed ≥12 months before the discovery of recurrent or metastatic disease.

### Treatment

One treatment cycle in this study was 14 days, determined by the chemotherapy interval.

The experimental group was administered anti-EGFR monoclonal antibody injections on the 1st and 8th day of a treatment cycle (14 days) and combined with chemotherapy, which consisted of anti-EGFR monoclonal antibody and irinotecan/calcium folinate (LV) on day 1 and 5-fluorouracil (5-FU) on days 1 and 2, and anti-EGFR monoclonal antibody on day 8, and was followed up until the 14th day of the cycle. The control group was treated with chemotherapy only, which consisted of irinotecan/calcium folinate (LV) on day 1 and 5-fluorouracil (5-FU) on days 1 and 2, followed by follow-up until day 14 of the cycle.

### Variable

Progression-free survival (PFS) of the disease.

The PFS duration (in months) is determined by a specially authorized Independent Radiology Review Committee (IRaC) through blinded review of imaging data. It is defined as the time from randomization to the first confirmed progression of disease by imaging or death from any cause within 90 days after the last tumor assessment or randomization, whichever is later (equivalent to 1.5 times the interval between two consecutive tumor assessments).

### Intercurrent Events and Handling Strategies

| **intercurrent events** | **processing strategy** | **note** |
| --- | --- | --- |
| Received other anti-tumor therapy prior to disease progression | **Hypothetical strategy** Data after the occurrence of a intercurrent event were not used when judged by sponsors and investigators to affect the evaluation of the primary endpoint indicator. | If other unplanned antitumor treatments were taken prior to the failure of the assigned regimen, it will be difficult to interpret the results as reflecting the effect of that treatment, and can therefore be handled with a hypothetical strategy. |
| Subjects withdrew from the experimental group due to anti-EGFR monoclonal antibody toxicity resulting in cancellation of 4 consecutive weeks of EGFR | **Treatment policy strategy** Data continued to be collected and used even when patients experienced concomitant events. | Reflects clinical practice. |
| Delayed chemotherapy due to toxicity of chemotherapeutic agents or discontinued due to chemotherapy overdose, but continued to receive anti-EGFR monoclonal antibody until progression of disease | **Treatment policy strategy** Data continued to be collected and used even when patients experienced concomitant events. | Reflects clinical practice. |
| Control group delayed chemotherapy due to toxicity of chemotherapeutic agents, or discontinued due to chemotherapy dosing | **Treatment policy strategy** Data continued to be collected and used even when patients experienced concomitant events. | Reflects clinical practice |
| undergo radical surgery for metastases | **While on treatment strategies** Data inclusion before subjects underwent radical surgery for metastases | Radical surgery can lead to an inability to judge efficacy. |

### Population-level Summary

Comparison of PFS (months) between the two groups of subjects and comparison of risk ratios between the two groups of subjects.

## Sample Size

Based on updated data from the CRYSTAL study in the literature, the median PFS for RAS wild-type subjects treated with (Cetuximab combined with FOLFIRI) and FOLFIRI monotherapy are 11.4 months and 8.4 months, respectively. This study is expected to enroll for 20 months, with a total study period of 36 months, a significance level of α=0.025 (one-sided), and requires observation of 339 events (PD) to detect a risk ratio of 0.7368 with 80% power. With a 1:1 ratio, the sample size is calculated to be 410 cases using PASS 13 sample size estimation software, considering a 20% dropout rate, the planned number of cases is 256 for Group A and 256 for Group B, totaling 512 cases.

## Research Interventions

**Experimental group: Recombinant anti-EGFR human-mouse chimeric monoclonal antibody (CMAB009) combined with FOLFIRI chemotherapy regimen.**

A treatment cycle consisting of anti-EGFR monoclonal antibody and irinotecan/calcium folinate (LV) on day 1 and 5-fluorouracil (5-FU) on days 1 and 2, and anti-EGFR monoclonal antibody on day 8, followed by follow-up until day 14 of the cycle.

**Control group: FOLFIRI (irinotecan, 5-fluorouracil, calcium folinate) chemotherapy regimen.**

A treatment cycle consisted of irinotecan/calcium folinate (LV) on day 1 and 5-fluorouracil (5-FU) on days 1 and 2, followed by follow-up until day 14 of the cycle.

Either anti-EGFR monotherapy or chemotherapy may be delayed due to toxicity. If treatment is delayed due to anti-EGFR monotherapy-related toxicity, the 14-day cycle of chemotherapy remains unchanged. Up to four consecutive weeks of anti-EGFR monotherapy infusion (not to exceed 28 days) may be canceled. If this time is exceeded, the subject should be withdrawn from anti-EGFR monotherapy, but may continue to receive chemotherapy until disease progression.

If chemotherapy is delayed due to toxicity of either chemotherapeutic agent (5-FU/LV or irinotecan), the other chemotherapy should be continued. If the subject develops toxicity to both chemotherapeutic agents, the chemotherapy should be postponed in its entirety. In all cases, however, the anti-EGFR monoclonal antibody infusion should be maintained on a 7-day weekly basis. Up to two consecutive cycles of chemotherapy (5-FU/LV or irinotecan) administration (not to exceed 28 days) may be canceled. The above treatment delays must not exceed 28 days during the study period, otherwise subjects must withdraw from chemotherapy but may continue to receive anti-EGFR monotherapy until disease progression.

In the event of permanent discontinuation of anti-EGFR monotherapy and chemotherapy due to toxic effects of both treatments, subjects will remain in the study (i.e., will continue to be evaluated for efficacy every 8 weeks) until disease progression.

Once further tumor assessment visits are not possible due to withdrawal of informed consent by the subject or for other reasons, the investigator should make every effort to finalize the data on tumor measurements; if possible, the subject should also undergo a Final Tumor Assessment (FTA) to collect additional information as needed.

No additional antitumor therapy should be administered to the subject prior to disease progression. After disease progression, all treatment with the investigational agent should be discontinued (if still receiving investigational therapy).

Treatment in both arms will be until disease progression confirmed by computed tomography (CT) or magnetic resonance imaging (MRI), or until the subject develops an intolerable AE or the subject withdraws informed consent on his/her own. If treatment with anti-EGFR monoclonal antibody is discontinued prior to PD (see above), subjects should continue chemotherapy until PD and undergo evaluation visits once every 8 weeks to assess efficacy by CT or MRI scan. If treatment with one component of chemotherapy is discontinued prior to PD (see above), subjects may continue to receive the other components of chemotherapy until PD and receive evaluation visits every 8 weeks to assess efficacy by CT or MRI scan.

No other antitumor therapy should be received prior to confirmation of PD. Once other antitumor therapy has been received prior to PD, a final tumor assessment visit is mandatory.

**Study cycle:** four main phases: pre-screening (>-14 days), screening period (-14 days), treatment period (chemotherapy, 14 days per cycle), complete treatment and follow-up period (follow-up at the end of the study, every 3 months), refer to the figure below.


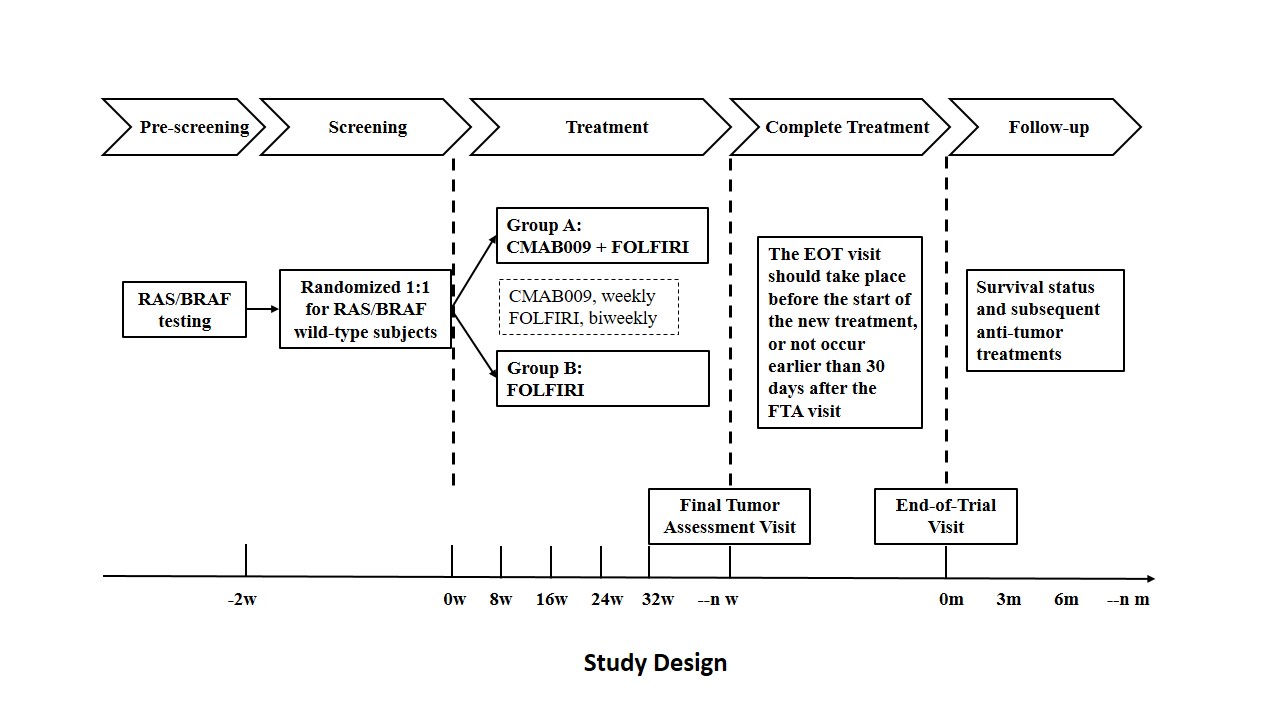


## Randomization

Only subjects with wild-type RAS/BRAF status in tumor tissue are eligible for randomization. Subjects who withdraw informed consent prior to randomization are not permitted to participate in the randomization process. Subjects who do not meet the inclusion criteria or meet the exclusion criteria after signing the informed consent form are also ineligible for randomization. Once a patient has signed the informed consent form, the reasons for not participating in randomization must be documented in the Case Report Form (CRF).

Upon completion of the screening period assessments, all eligible subjects will be randomly assigned to Group A and Group B in a 1:1 ratio. Randomization will be conducted based on a central randomization system, with the stratification factors being the study site and ECOG performance status (two levels, ECOG performance status of 0 and 1). The designated person in charge at the participating center logs into the randomization system, enters the subject's relevant information, and the system will then display the group assignment for that case. Subsequently, this person will notify the investigator of the group to which the subject has been assigned for the study treatment. The randomization number of the enrolled case must be recorded in the electronic Case Report Form (eCRF), and each subject can only participate in one randomization assignment.

## Blind Design and Bias Control

In the Phase II/III clinical trial, the most notable adverse reaction associated with CMAB009 was skin toxicity, with the incidence of rash in the experimental group being 4-5 times higher than that in the control group (71.6% vs 15.3%). Skin toxicity is the most commonly reported adverse reaction in the safety information for Erbitux^®^, and the severity of rash has been positively correlated with treatment efficacy. Skin toxicity may even serve as a clinical surrogate marker for the efficacy of cetuximab, although the exact mechanism of this relationship remains unclear. Given the differences in skin toxicity between treatment groups, this study is designed as an open-label trial. The primary endpoint, progression-free survival (PFS), and the secondary endpoint, objective response rate (ORR), will be assessed through independent imaging with blinded review to control for potential bias in efficacy evaluation.

## Interim

This study set a final analysis, not involving a midterm analysis.

## Follow Up

The final on the primary endpoint of PFS will be given this time. This study will remain open to collect overall survival (OS) until at least 80% of randomized subjects die or are lost to follow-up.

## Study Flowchart

|  | **Pre-treatment** | | **Chemotherapy (14 days per cycle)** | | **Evaluation Visit (very 8 weeks) ^2^** | **FTA ^3^** | **EOT ^4^** | **Follow-up (every 3 months)** |
| --- | --- | --- | --- | --- | --- | --- | --- | --- |
|  | **Pre-screening ^1^** | **Screen** |  |  |  |  |  |  |
|  | **>-14 d** | **—14 d** | **D1** | **D8** |  |  |  |  |
| **Informed consent** | **√** |  |  |  |  |  |  |  |
| **RAS/BRAF testing** | **√** |  |  |  |  |  |  |  |
| **Diagnosis of tumor** | **√** |  |  |  |  |  |  |  |
| **Subject number assignment** | **√** |  |  |  |  |  |  |  |
| **Demographic data** |  | **√** |  |  |  |  |  |  |
| **Inclusion and exclusion criteria** |  | **√** |  |  |  |  |  |  |
| **Medical history and previous treatments** |  | **√** |  |  |  |  |  |  |
| **Physical examination** |  | **√** |  |  | **√** | **√** |  |  |
| **Vital signs** |  | **√** |  |  | **√** | **√** |  |  |
| **ECOG score** |  | **√** |  |  | **√** | **√** |  |  |
| **Routine blood test ^5^** |  | **√** | **√** | **√** | **√** | **√** |  |  |
| **Routine urine test ^5^** |  | **√** | **√** |  | **√** | **√** |  |  |
| **Hepatic and renal function ^5^** |  | **√** | **√** |  | **√** | **√** |  |  |
| **Hepatitis B panel test and/or HBV-DNA test** |  | **√** |  |  |  |  |  |  |
| **HIV antibody test** |  | **√** |  |  |  |  |  |  |
| **Electrolyte test** **^5^** |  | **√** | **√** |  | **√** | **√** |  |  |
| **ECG ^6^** |  | **√** | **√** |  | **√** | **√** |  |  |
| **Pregnancy test (if applicable)** |  | **√** |  |  |  |  |  |  |
| **Tumor assessment (radiography)** |  | **√** |  |  | **√** | **√** |  | **√^16^** |
| **EORTCQLQ-C30 questionnaire** |  | **√** |  |  | **√** | **√** |  |  |
| **Randomization** |  | **√** |  |  |  |  |  |  |
| **Weight and BSA ^7^** |  |  | **√** |  | **√** |  |  |  |
| **Administration of anti-EGFR monoclonal antibody ^8^** |  |  | **√** | **√** |  |  |  |  |
| **Administration of FOLFIRI chemotherapy ^9^** |  |  | **√** |  |  |  |  |  |
| **CEA and CA19-9 test ^10^** |  | **√** |  |  | **√** | **√** |  |  |
| **Blood samples for ADA ^11^** |  |  | **√** |  |  |  | **√** |  |
| **Blood samples for PK ^12^** |  |  | **√** | **√** |  |  |  |  |
| **Assessment of adverse events ^13^** |  | **√** | **√** | **√** | **√** | **√** | **√** |  |
| **Concurrent medications and current medical interventions ^14^** |  | **√** | **√** | **√** | **√** | **√** | **√** |  |
| **Survival status** |  |  |  |  |  |  |  | **√** |
| **Subsequent anti-tumor treatments** |  |  |  |  |  |  |  | **√** |
| **Skin toxicity still present at the EOT visit ^15^** |  |  |  |  |  |  |  | **√** |

Note: Cranial CT or MRI, and bone ECT should be performed in subjects suspected of having brain or bone metastases; routine blood tests, hepatic and renal function tests, and imaging examinations of the thorax, abdomen, and pelvis may be appropriately increased if deemed necessary by the investigator.

The treatment start window for each cycle is ±3 days; the visit start window for each visit cycle is ±3 days.

Recent results of routine blood tests, routine urine tests, hepatic and renal function tests, electrolytes, and ECG within 7 days prior to randomization are acceptable for baseline evaluation.

CT or MRI imaging within 4 weeks prior to the first administration of medication is acceptable for baseline tumor evaluation.

1: In the pre-screening process, tumors must be confirmed as metastatic colorectal cancer and both RAS/BRAF genotypes must be wild-type to proceed to the screening phase.

2: Evaluation visit intervals are conducted every 8 weeks until study withdrawal.

3: The final tumor assessment (FTA) visit is conducted after all study treatments have been completed.

4: The end-of-trial (EOT) visit must be conducted before the initiation of new anti-tumor treatment or at least 30 days after the final tumor assessment.

5: Within 3 days prior to administration.

6: Electrocardiogram (ECG) examinations are conducted once every 2 weeks during the first 3 cycles and once every 4 weeks thereafter (on the day of administration or within 3 days prior to administration).

7: Body weight is measured and BSA (Body Surface Area) is calculated on the first day of each treatment cycle. If a subject's body weight increases or decreases by more than 5% compared to the weight at the last dose adjustment, drug dosage adjustment is required; otherwise, no dosage adjustment is needed (within 3 days prior to administration).

8: Only applicable to Group A, the investigator must closely monitor the subject's condition during and for at least 1 hour after the infusion process.

9: Chemotherapy continues on the second day of the treatment cycle.

10: Tumor marker tests are conducted in conjunction with the evaluation visit, i.e., once every 8 weeks.

11: Only applicable to Group A, blood samples are collected before research treatment on the first week, and at weeks 4, 8, 16, 32, and the end-of-study visit, totaling 6 blood collections.

12: Only applicable to specific subjects, the flowchart does not indicate a clear blood collection time point. For detailed information, refer to Section 7.6.

13: Adverse events need to be continuously recorded until symptom resolution or outcome is predictable.

14: Concurrent medications and current medical interventions need to be continuously recorded until the end of the study.

15: Skin toxicity present at the EOT visit will be followed up for a maximum of 6 months.

16: During the follow-up period, if a subject discontinues treatment for reasons other than disease progression or withdrawal of informed consent, efforts should be made to collect tumor imaging data for tumor assessment until disease progression or initiation of other anti-tumor treatments (whichever occurs first).

# Evaluation Indicators

## Efficacy Indicators

### Primary Efficacy Indicators.

The PFS duration (in months) is determined by a specially authorized Independent Radiology Review Committee (IRaC) through blinded review of imaging data. It is defined as the time from randomization to the first confirmed progression of disease by imaging or death from any cause within 90 days after the last tumor assessment or randomization, whichever is later (equivalent to 1.5 times the interval between two consecutive tumor assessments). Tumor progression and response are assessed according to RECIST criteria version 1.1. Tumor assessments are conducted every 8 weeks after randomization until the end of the study.

If a subject has neither tumor progression nor death after the last tumor assessment in the study or within 90 days after randomization, they are censored at the date of the last tumor assessment.

If subjects underwent radical surgery for metastases during the course of the study, they were judged according to the while on treatment strategies by tumor assessment on the last preoperative day: if tumor progression or death did not occur at any time prior to the surgery, the day prior to the day of radical surgery was used as the cut-off date; if tumor progression or death occurred prior to undergoing radical surgery, PFS was performed according to the definition.

The research hypothesis of this study was whether the risk ratio for the treatment of RAS/BRAF gene wild-type, metastatic colorectal cancer with anti-EGFR human-mouse chimeric monoclonal antibody injection (CMAB009) in combination with FOLFIRI as compared to FOLFIRI monotherapy would be less than 1.

H_0_: The risk ratio of anti-EGFR human-mouse chimeric monoclonal antibody injection (CMAB009) in combination with FOLFIRI versus FOLFIRI monotherapy was ≥1.

H_1_: The risk ratio of anti-EGFR human-mouse chimeric monoclonal antibody injection (CMAB009) in combination with FOLFIRI versus FOLFIRI monotherapy was <1.

α= 0.025 (one-sided).

### Secondary Efficacy Indicators

1. Objective Response Rate (ORR).

The investigators and IRaC will use RECIST 1.1 to evaluate objective response rate. ORR will be calculated according to the details of target or non-target lesions collected in the case report form (CRF) (overall response will be confirmed based on the assessment of target and non-target lesions and the presence or absence of new lesions; remission is shown in the table below). Post-baseline tumor assessment should be performed using the same evaluation tools (CT, MRI) as at baseline and preferably in one hospital.

Table 1 Evaluation of Target Lesions

| Complete Response (CR) | Disappearance of all target lesions. Any pathological lymph nodes (whether target or non-target) must have reduction in short axis to < 10 mm |
| --- | --- |
| Partial Response (PR) | At least a 30% decrease in the sum of diameters of target lesions, taking as reference the baseline sum diameters |
| Progressive Disease (PD) | At least a 20% increase in the sum of diameters of target lesions, taking as reference the smallest sum on study (this includes the baseline sum if that is the smallest on study). In addition to the relative increase of 20%, the sum must also demonstrate an absolute increase of at least 5 mm. (Note: the appearance of one or more new lesions is also considered progression) |
| Stable Disease (SD) | Neither sufficient shrinkage to qualify for PR nor sufficient increase to qualify for PD, taking as reference the smallest sum diameters while on study |

Table 2 Evaluation of non-target lesions

| Complete Response (CR) | Disappearance of all non-target lesions and normalisation of tumor marker level. All lymph nodes must be non-pathological in size (<10 mm short axis) |
| --- | --- |
| Non-CR/Non-PD | Persistence of one or more non-target lesion(s) and/or maintenance of tumor marker level above the normal limits |
| Progressive Disease (PD) | Unequivocal progression of existing non-target lesions |

The objective response rate was calculated based on the percentage of evaluable subjects with complete response (CR) and partial response (PR) in the subject's overall efficacy (ORR=CR+PR). The definition of the best overall efficacy for a subject is the best confirmed efficacy observed at all time points of efficacy assessment. The possible best overall efficacy results are CR, followed by PR, SD, and PD.

The confirmation steps for the potential best efficacy at two time points are summarized in Table 3. Except for CR, the two time points do not need to be consecutive.

Table 3 Best overall response when confirmation of CR and PR required

| **Overall response First time point** | **Overall response Subsequent time point** | **BEST overall response** |
| --- | --- | --- |
| CR | CR | CR |
| CR | PR | SD, PD or PR ^a^ |
| CR | SD | SD provided minimum criteria for SD duration met ^b^, otherwise, PD |
| CR | PD | SD provided minimum criteria for SD duration met ^b^, otherwise, PD |
| CR | NE | SD provided minimum criteria for SD duration met ^b^, otherwise NE |
| PR | CR | PR |
| PR | PR | PR |
| PR | SD | SD |
| PR | PD | SD provided minimum criteria for SD duration met ^b^, otherwise, PD |
| PR | NE | SD provided minimum criteria for SD duration met ^b^, otherwise NE |
| NE | NE | NE |

Note 1: CR is complete response, PR is partial response, SD is stable disease, PD is progressive disease, and NE is not evaluable. Superscript "a": If a CR is truly met at first time point, then any disease seen at a subsequent time point, even disease meeting PR criteria relative to baseline, makes the disease PD at that point (since disease must have reappeared after CR). Best response would depend on whether minimum duration for SD was met. However, sometimes ‘CR’ may be claimed when subsequent scans suggest small lesions were likely still present and in fact the patient had PR, not CR at the first time point. Under these circumstances, the original CR should be changed to PR and the best response is PR.

Note 2: In column 2 of the table, "Subsequent time point" refers to the time of the next tumor assessment, and there is no need to limit the length of time between two tumor assessments for the time being.

Note 3: Superscript "b" defines sufficient time as defined in RECIST 1.1, at least 6 weeks inclusive. The start time is defined as the beginning of baseline, and the end time is defined as the closest SD to the first time point of total response (if multiple SDs are present).

Note 4: If CR or PR occurs only at the last evaluation time point collected during the course of each evaluation time point, it is SD if the time point is SD and sufficient time is available, otherwise it should be NE.

1. One-year Overall Survival Rate, Two-year Overall Survival Rate, and Overall Survival Time

One-year overall survival rate definition: The proportion of subjects alive at one year from randomization.

Two-year overall survival rate definition: The proportion of subjects alive at two years from randomization.

Overall survival time: Defined as the time from randomization to death (in months). For subjects still alive or lost to follow-up as of the data analysis cutoff date, survival is censored at the subject's last known alive time.

1. Disease Control Rate (DCR).

Percentage of subjects responding with CR, PR, and SD (DCR=CR+PR+SD) using data assessed by IRaC, according to RECIST 1.1 in Tables 1 and 2 above.

1. Clinical Benefit Rate (CBR).

Proportion of subjects whose tumors were assessed to be in CR, PR, and SD for 24 weeks or more according to RECIST 1.1 in Tables 1 and 2 above, using data assessed by IRaC.

1. Duration of Response (DOR).

Data assessed using IRaC, in accordance with RECIST 1.1 in Tables 1 and 2 above, for subjects with a response of CR or PR, from the date of first presentation (CR or PR) to the date of first disease progression or death. Subjects were censored if they did not have an endpoint event, and the date of censoring was the date of the last imaging evaluation.

1. Time to Response (TTR).

Data assessed using IRaC were performed according to RECIST 1.1 in Tables 1 and 2 above for subjects with a response of CR or PR.TTR was defined as the time from the date of randomization up to the date of the first appearance of response (CR or PR) according to RECIST version 1.1.

1. Quality of Life Assessment Indicators (QOL).

Quality of life assessment (QOL) is conducted using the EORTC QLQ-C30 questionnaire, with individual item categorical scores linearly transformed to a 0-100 scale. The questionnaire is self-reported, consisting of 30 items, and subjects are asked to complete it carefully. Assessments are conducted at baseline and subsequent visits every 8 weeks until withdrawal from the study and entry into the follow-up period.

1. Resection Rate of Hepatic Metastasis

The resection rate of hepatic metastasis = number of subjects undergoing radical surgery for liver metastases divided by the number of subjects;

Radical hepatic metastases resection rate = number of subjects with complete resection (R0 resection) divided by the number of subjects.

## Safety Evaluation Indicators

- Drug exposure and treatment compliance
- Adverse events and incidence rates as defined using CTC AE version 4.03
- Physical examination
- Vital signs and weight: Vital signs include heart rate/pulse, respiration, temperature, blood pressure
- ECOG
- 12-lead electrocardiogram.
- Laboratory parameters: Clinically significant changes in routine blood and urine tests, hepatic and renal function, and chest X-rays

## Other Indicators

- Immunogenicity (ADA)
- Pharmacokinetics

Concomitant taken at any time during the pre-existing and study periods or within 30 days of final study dosing.

# Statistical Analysis

## Analysis Datasets

The following populations will be used as data for the study.

Full Analysis Set (FAS): According to the basic principle of Intention-To-Treat (ITT), the main efficacy indexes should include all randomized subjects, i.e., it is necessary to follow up the results of all randomized subjects completely, but it is often difficult to achieve in practice. Therefore, this trial was conducted using the full set. The full set refers to the set of subjects as close as possible to the principle of intentionality, which is derived from all randomized subjects with minimal and reasonable exclusion of certain cases.The FAS population will include all subjects randomized to the study and receiving at least 1 study treatment. Subjects after randomization will be, but if patients who are not wild-type for the RAS/BRAF gene are randomized, these patients will not be entered into the full set.

Per Protocol Set (PPS): A sample of cases that meets the requirements of the trial protocol, has good compliance, and has completed all the requirements of the trial, also known as "evaluable cases" or "eligible cases". A sample that is a subset of the full set. It is a subset of the full set, in which subjects are more adherent to the protocol, have completed a pre-determined minimum amount of treatment, have measurements on key variables, and have not committed major violations of the protocol. This dataset will be reviewed after the completion of the trial and finalized before the database is locked. Cases that violate the trial protocol, such as failure to achieve the prescribed treatment volume, absence of a primary variable, or use of an explicitly prohibited medication that affects the evaluation of the primary endpoint, will not be included, and justification will be provided for exclusion from this dataset.

The Per-Protocol population will not include all ITT populations that meet one or more of the following criteria:

- Randomization failure (e.g., receiving incorrect treatment)
- No evidence of mCRC at baseline (e.g., no target lesions with the longest diameter ≥1cm at baseline)
- Receiving study treatment for ≤8 weeks, excluding subjects who die or have PD within the first 8 weeks after starting treatment
- Receiving unauthorized chemotherapy or other treatments during the study period
- Significant protocol deviations affecting the evaluation of the primary endpoint occurred.

Safety Analysis Set (SAS): refers to all subjects who received at least one treatment after randomization, i.e., adverse events and adverse reactions should be reported whenever a subject was administered one or more drugs from the specified group, regardless of whether or not the subject was included in the compliant set.

The Response evaluable Set (RES) is a subset of the FAS and refers to subjects in the full set who had at least one tumor assessment at post-baseline follow-up.

PK Parameter Set (PKPS): Subjects who provided PK blood collection according to the protocol, the designated population of subjects in Group A. Subjects who received at least one study drug and had at least one pharmacokinetic parameter. Subjects who received at least one dose of study drug and had at least one pharmacokinetic parameter.PKPS was used for descriptive statistics of pharmacokinetic parameter data for subjects.

Immunogenicity (ADA) set: all patients enrolled and those who used the study at least once and had data from baseline and at least one post-baseline ADA evaluation.

Demographic and baseline characteristics will be performed using FAS; primary efficacy endpoint, secondary efficacy endpoints will be performed using both FAS and PPS; safety endpoints will be performed using SS; RES sets will also be utilized for objective response rate (ORR), disease control rate (DCR), clinical benefit rate (CBR), time to remission (DOR), and time to remission (TTR), which have tumor remission assessment metrics were performed; PKPS for pharmacokinetic parameters; ADA for immunogenicity metrics.

## General Principles of Statistics

### Software and Data Transfer

Statistics will be performed using SAS 9.4 (or above).

Statistical tables and lists will be generated as RTF files and statistical graphs as PNG files.

### Reporting of Results

FAS, PPS, and RES were performed according to the study subgroups assigned by randomization, and SS was performed according to the actual study intervention received.

Descriptive statistics were mainly performed as descriptive statistics. Continuous variables were described by means, differences, medians, quartiles, minimum and maximum values, while count and rank information was described by frequencies and percentages.

The statistics and number of decimal places are shown in the following table.

| **name** | **clarification** | **Number of decimal places (dp)** |
| --- | --- | --- |
| quantitative information | | |
| N | Number of subjects with no missing results | 0 dp |
| Mean | arithmetic mean (math.) | Decimal digits of variables +1dp |
| SD | differ from | Decimal digits of variables +1dp |
| Median | upper quartile | Consistent with the number of decimal places in the variable |
| Q1 | 25th percentile | Consistent with the number of decimal places in the variable |
| Q3 | 75th percentile | Consistent with the number of decimal places in the variable |
| Min | minimum value | Consistent with the number of decimal places in the variable |
| Max | maximum values | Consistent with the number of decimal places in the variable |
| Missing | Number of missing cases | 0 dp |
| Qualitative (classification and ranking) information | | |
| n | Number of subjects with an evaluation indicator of a given value | 0 dp |
| % | Percentage (0-100%) | 2 dp |
| statistic | | |
| 95% CI | 95% confidence interval | Quantitative indicators are the number of decimal places of the variable + 1 dp, and qualitative indicators are 2 dp. |

Note:The maximum number of decimal places is 4 decimal places; PFS and OS time (months) related metrics are described using 3 decimal places.

Comparisons of the general conditions of the two groups will be made using the appropriate method depending on the type of indicator. Comparisons of quantitative data between groups will be made using the group t-test (normality and chi-square) or the Wilcoxon rank-sum test (normality or chi-square from time to time) in a parallel-group design, and comparisons with the baseline values at screening will be made using the paired t-test (normality and chi-square) or the Wilcoxon rank-sum test (normality or chi-square from time to time) to compare differences within groups; dichotomous data will be compared using chi-square tests (smallest expected frequency ≥ 5 in all grids) or exact probability methods. or variance chi-square from time to time) to compare the before-and-after differences within groups; for dichotomous data, the chi-square test (smallest expected frequency ≥5 in all grids) or the exact probability method (presence of grids with expected frequency <5) was used; and for hierarchical data, the Wilcoxon rank-sum test (no center effect) or the CMH test (center effect required) was used.

Unless otherwise stated, all statistical tests were performed using a two-sided test with a test level of α=0.05 and confidence intervals of 95%. p-values are retained to 3 decimal places and will be denoted as "p<0.001" for p<0.001.

### Rules for Handling Missing Data

Missing data will be handled according to the division of the data set in which they are located, and for primary efficacy data, since they are survival data, they do not need to be filled in, but the handling of missing dates in special cases is described in section 4.2.4 below. During the data management process, the data in the database will be logically verified and unreasonable/missing data will be found and queries will be sent to the investigator in the form of a query (Query), and the unreasonable/missing data will be processed on the basis of the investigator's written response until all unreasonable/missing data have been resolved before the database can be locked.

For safety data, where the date is missing, in general, when only part of the date is known, the following rules are used to calculate the time:

- If the start time is partially missing, and only the day is missing, then replace it with the first day of that month.
- If the end date is partially missing and only the day is missing, the last day of the month is substituted.
- If both the day and month of a date are missing, the date is treated as missing.

For concomitant therapy data, the relevant missing dates are treated as follows:

- If the start time is partially missing, and only the day is missing, then replace it with the first day of that month.
- If the end date is partially missing and only the day is missing, the last day of the month is substituted.
- If both the day and month of a date are missing, the date is treated as missing.

Missing dates related to medical history, surgical treatment of the primary tumor, radiation/chemotherapy, and other treatments are not filled in.

Rules for PK data or outlier data:

- If PK concentration data were not collected or were missing, the missing data were not filled in, and the missing data were not included in the statistical summaries but were tabulated only.
- The PK parameter is calculated as the actual sampling time, and when the actual sampling time of a PK sample exceeds the sampling time window specified by the protocol, the concentration data at that time point need to be excluded and then descriptive statistics are performed again.
- If the PK parameter cannot be calculated from the PK concentration, it will be shown as "Not calculated (NC)" in the list and will be treated as a missing value in the statistical summary table. The value of the PK parameter for "NC" will also be treated as missing in the statistical modeling.

In general, all measurements will be used for statistical purposes. If there are clear special reasons for outliers that affect the statistics, they will be treated as missing and labeled in the statistical results/reports and will be characterized if necessary.

Missing text data is shown in the list as ""; missing numeric data is shown in the list as "." in the list. If the data are recorded as "Not Applicable"/"NA" and "Unable to Evaluate"/"NK", they will be shown as original records in the description of the list and treated as missing data in the statistics.

### Rules for Handling Relevant Outcomes and Dates During Survival Aanalysis

Multiple dates are involved in the tumor assessments of each visit according to RECIST 1.1, including the dates of imaging used to evaluate target lesions, non-target lesions, new lesions, and the date of the overall response assessment. These dates often do not fall on the same day, and the date of the overall tumor efficacy assessment may be later than the date of the imaging study.

Therefore, the rules that need to be harmonized in calculating the overall efficacy date for each tumor are as follows:

- When the overall efficacy is "PD", the date of the overall tumor efficacy is the earliest of the dates of the current imaging used to assess the target lesion, non-target lesion, new lesion, and the date of the tumor efficacy assessment.
- When the overall tumor outcome was "PD" and the date of disease progression was within the interval of at least 2 consecutive tumor assessments (missing), the outcome was defined as the occurrence of an event, and the date of the event was defined as the date of disease progression collected.
- The outcome was defined as censored when the overall tumor efficacy was "PD" and the date of disease progression was after an interval of at least 2 consecutive (missing) tumor assessments, with the date of censoring defined as the date of the last validated tumor assessment prior to disease progression; if there was no validated tumor assessment prior to disease progression, the censoring was made on the date of the first dose of the drug.
- If the overall tumor outcome is "non-PD", the date of the overall tumor outcome is the latest of the dates of imaging of the target lesion, the non-target lesion, the new lesion, and the date of the evaluation of the tumor's efficacy, when it is used to assess the overall tumor outcome.
- Disease progression was not documented prior to initiation of new anti-tumor therapy, the outcome was defined as censored, and the date of censoring was defined as the date of the last valid tumor assessment prior to initiation of new anti-tumor therapy; if there was no valid tumor assessment prior to initiation of new anti-tumor therapy, censoring was on the date of the first dose.
- Missing baseline tumor assessment, outcome defined as censored, date of censoring defined as date of first dosing.
- Missing tumor assessment after baseline, with the outcome defined as censored and the date of censoring defined as the date of the first dose.
- Baseline investigator assessment of having a target lesion was eligible for enrollment, but specifically authorized IRaC assessment of no target lesion resulted in missing baseline and post-baseline tumor assessments, with the outcome defined as censored and the date of censoring defined as the date of the first dose.

### Baseline Definitions

Baseline values were defined as the last value measured prior to administration for statistical purposes, unless otherwise stated.

### Data Derivation and Conversion

- Age (years) = (date of informed consent - date of birth + 1)/365.25, rounded down.
- Determination of relevance to the experimental drug: Definitely related, probable related, possibly related should be determined as drug-related adverse events.
- The actual follow-up date was used to calculate PFS time, overall survival time (OS), duration of response (DOR), and time to response (TTR). The calculation rules were based on the definition of endpoint indicators in the protocol. The calculation formula based on date is expressed as:

PFS time = date of PD - date of randomization + 1.

Overall survival time (OS) = date of death - date of randomization + 1.

Duration of response (DOR) = first occurrence of PD or death - first occurrence of CR or PR + 1;

Time to response (TTR) = first occurrence of CR or PR - date of randomization + 1.

Note: The above time indicators are expressed in "days" and are converted to "months" by dividing by 30.

## Trial Completion and Subject Distribution

Calculate the number and proportion of subjects screened, enrolled, completed, and discontinued from the trial for the overall and each group, and the number and proportion of subjects in each treatment group with each reason for discontinuation. Calculate the number and proportion of subjects in the full analysis set, the safety set, the per-protocol set, the responder evaluable set, the PK parameter set (PKPS), and the immunogenicity analysis set.

Flowchart the distribution of subjects.

## Protocol Deviations

The number and incidence of subjects with protocol deviations were summarized by group, severity of protocol deviation, and type of protocol deviation, and a list of subjects with protocol deviations was made. The number and incidence of subjects with protocol deviations due to the COVID-19 epidemic were also summarized by group, severity of protocol deviation, and type of protocol deviation.

List subjects who deviated from the protocol.

Protocol deviations were performed in all SS populations.

## Treatment Compliance

Statistical description of compliance according to qualitative indicators. Subjects with poor compliance were described using a list of specific reasons of poor compliance.

Poor compliance was defined as subjects missing >2 consecutive infusions of anti-EGFR monotherapy or chemotherapy for non-medical reasons. Poor compliance usually results in discontinuation of the anti-EGFR monotherapy or chemotherapy, which will require a case-by-case negotiation between the investigator and sponsor as to whether to discontinue the subject's treatment.

Compliance was performed in all SS populations.

## Concomitant Medication and Concomitant Treatment

In the full analysis set population, summarize the frequency and proportion of subjects for each concomitant medication or concomitant treatment by treatment group, and summarize the frequency and proportion of concomitant medications or concomitant treatments according to the ATC or International Medical Dictionary coding results.

Concomitant medication refers to drug treatment received at least once after the use of the study drug. If the discontinuation date of the concomitant medication is the same as the first date of use of the study drug, or if the start date or end date of the concomitant medication is partially missing, it is considered a concomitant medication unless there is a sufficient explanation. Concomitant medications are divided into medications for accompanying diseases, AE medications, preventive medications, and other reasons for medication use, and will be coded using the WHODrug Global Chinese version of March 2022. Analyze the coded concomitant medications. Summarize the number and proportion of subjects with at least one concomitant medication, grouped by therapeutic classification (ATC) and preferred term (PN).

Concomitant treatment refers to non-drug treatment received at least once after the use of the study drug. If the discontinuation date of the concomitant treatment is the same as the first date of use of the study drug, or if the start date or end date of the concomitant treatment is partially missing, it is considered a concomitant treatment unless there is a sufficient explanation. Concomitant treatments are coded using the International Medical Dictionary MedDRA version 25.0 Chinese, and analyze the coded concomitant treatments. Summarize the number and proportion of subjects with at least one concomitant treatment, as well as the number and proportion of subjects with concomitant treatments grouped by system organ classification (SOC) and preferred term (PT).

List the concomitant medications and concomitant treatments for medications for accompanying diseases, AE medications, preventive medications, and other reasons for medication use. Concomitant medications and treatments will be conducted in all SS populations.

## Demographic and Baseline Characteristics

Statistical descriptions of demographic data (such as age, gender, etc.), primary tumor diagnosis, tumor metastasis sites, past medical history, past tumor treatment history, surgical treatment, and radiochemotherapy status at the baseline period of all enrolled subjects are provided. Appropriate statistical analysis methods are selected for intergroup comparisons based on the data type (quantitative, binary, or ordinal data). Quantitative data are analyzed using t-tests (for normally distributed and equal variance data) or Wilcoxon rank-sum tests, and count data are analyzed using chi-square tests or Fisher's exact probability method.

Demographic and baseline data analysis is conducted in the FAS (Full Analysis Set) population.

## Statistical Analysis of Primary Estimand

### Analysis Sets

The primary estimand will be conducted on the FAS (Full Analysis Set) and PPS (Per Protocol Set) populations.

### Main Statistical Analysis of Primary Estimand

A stratified (stratification factors include center and ECOG score) Cox proportional hazards regression model is used to estimate the hazard ratios between the treatment groups of anti-EGFR human-mouse chimeric monoclonal antibody injection (CMAB009) combined with FOLFIRI versus FOLFIRI alone in each stratum and the overall hazard ratio. A stratified (stratification factors include center and ECOG score) log-rank test (two-sided significance level of 0.05) is used to test the efficacy difference between the anti-EGFR human-mouse chimeric monoclonal antibody injection (CMAB009) combined with FOLFIRI and FOLFIRI alone treatment groups. The Kaplan-Meier method is used to estimate the median progression-free survival and its 95% confidence interval for both groups.

Due to the selection of the intercurrent events strategy, progression-free survival time is defined as the time from randomization to the first occurrence of disease progression confirmed by imaging based on IRaC (Independent Radiology Central) blinded independent review, or any cause of death within 90 days after the last tumor assessment or after randomization (whichever date is later, equivalent to 1.5 times the interval between two consecutive tumor assessments). The handling strategies for various intercurrent event strategies when missing determination conditions are described in Section 4.2.4 regarding the rules for handling relevant outcomes and dates during survival analysis.

### Sensitivity Analysis of the Primary Estimand

| Sensitivity Analysis 1 | To evaluate the robustness of the disease progression assessment based on the IRaC blinded independent review method, sensitivity analysis is conducted using data estimated from the assessments made by the central investigators and imaging experts. The progression-free survival time based on the investigators' judgment is defined as the time from randomization to the first occurrence of disease progression determined by the central investigators and imaging experts, or any death due to any cause within 90 days after the last tumor assessment or after randomization (whichever is later, equivalent to 1.5 times the interval between two consecutive tumor assessments). |
| --- | --- |
| Sensitivity Analysis 2 | Sensitivity analysis may also be required for cases where tumor assessment visits are missed before disease progression. This is because, if the bias introduced by the missed tumor assessment visits before disease progression is not considered, it may overall prolong the progression-free survival period. The potential bias has been controlled in the main analysis through censoring rules, and in the sensitivity analysis, event data occurring after two missed tumor assessments can be used (i.e., no censoring is applied, and progression is analyzed based on the determined disease progression date/death date). |
| Sensitivity Analysis 3 | For subjects who started new anti-tumor treatment before disease progression, or who withdrew from the study before disease progression and did not receive new anti-tumor treatment during the follow-up period but had imaging progression/death, censoring was performed in the main analysis according to the hypothetical strategy, and data after the occurrence of intercurrent events were no longer used. In the sensitivity analysis, PFS can be calculated using the date when new anti-tumor treatment was started, or the date of imaging progression/death after withdrawal from the study determined by the IRaC blinded independent review (whichever occurs first), and sensitivity analysis can be conducted. |
| Sensitivity Analysis 4 | Some subjects may have target lesions at baseline that meet the inclusion criteria according to the investigators' assessment, but no target lesions after special authorization by the IRaC, leading to missing baseline and post-baseline tumor assessments. For these subjects, the main analysis is conducted with censoring at the first dosing date, and to more objectively evaluate the situation where target lesions exist, these subjects can be excluded for sensitivity analysis. |
| Sensitivity Analysis 5 | Some subjects may have target lesions at baseline that meet the inclusion criteria according to the investigators' assessment, but no target lesions after special authorization by the IRaC, leading to missing baseline and post-baseline tumor assessments. For these subjects, the main analysis is conducted with censoring at the first dosing date. However, these subjects also have overall efficacy assessment results based on non-target lesions. Investigators suggest conducting sensitivity analysis of the main indicator based on the efficacy assessment results of non-target lesions, where the handling method for the outcome assessment of non-target lesions is consistent with the rules for handling relevant outcomes and dates during survival analysis described in Section 4.2.4. |

Additionally, the statistical analysis of the primary endpoint based on the Per Protocol Set (PPS) population can also serve as a form of sensitivity analysis.

### Stratified Analysis of the Primary Estimand

(1) Stratification will be conducted according to ECOG performance status (2 levels, i.e., ECOG score of 0 and 1), number of baseline tumor metastatic sites (2 levels, i.e., fewer than 3 and 3 or more sites), and center. Descriptive statistics will be performed within each stratum: If significant effects are found within the subgroups, an interaction analysis between treatment groups will be conducted.

(2) If applicable, stratification will be conducted based on ADA and Nab status, with stratified statistics for the efficacy of ADA-positive and -negative subjects, and Nab-positive and -negative subjects. ADA-positive subjects are defined as those with at least one positive ADA test result after administration, and Nab-positive subjects are those with at least one positive Nab test result after administration.

### Subgroup Analysis of the Primary Estimand

Based on clinical circumstances and research information, this study plans to conduct subgroup analyses for the following subgroups to further evaluate the efficacy differences between the two treatment plans in different subgroup populations. The specific subgroup definitions are as follows:

- A subgroup population composed of subjects excluding those with the primary tumor located in the right hemicolon (including ascending colon, cecum, ileocecal valve, right hemicolon, hepatic flexure, transverse colon, and splenic flexure);
- A subgroup population composed of subjects excluding those who have undergone curative surgery for liver metastases;
- A subgroup population composed of subjects excluding those affected by the COVID-19 pandemic (treatment interruptions/delays, tumor assessment interruptions/delays).

## Secondary Estimand

Secondary estimand will be conducted on the Full Analysis Set and Per Protocol Set populations. In addition, for indicators related to tumor relief assessment, such as Objective Response Rate (ORR), Disease Control Rate (DCR), Clinical Benefit Rate (CBR), Duration of Response (DOR), and Time to Response (TTR), supplementary analysis will also be conducted using the Response Evaluable Set (RES) population. For secondary estimand based on treatment policy strategy, general statistical analysis methods will be followed, without imputation of missing data and without sensitivity analysis or supplementary analysis (except for special instructions).

Secondary efficacy indicators include:

**Objective Tumor Response Rate (ORR):** Investigators and IRaC will use the RECIST 1.1 criteria to assess the objective tumor response rate. The Case Report Form (CRF) will collect detailed information on target or non-target lesions to calculate tumor objective response. Tumor assessments after baseline should use the same evaluation methods (CT, MRI) and preferably be conducted at the same hospital. For subjects still on medication at the data cutoff date who have not yet undergone the best overall efficacy evaluation during the study period, the ORR assessment will be processed as not evaluated according to actual circumstances.

The tumor objective response rate is calculated based on the percentage of evaluable subjects with complete response (CR) and partial response (PR) (ORR = CR + PR).

Additionally, the following subgroup and stratified analyses will be conducted for the tumor objective response rate (ORR):

(1) The definitions of specific subgroups are as follows:

- A subgroup population composed of subjects excluding those with the primary tumor located in the right hemicolon (including ascending colon, cecum, ileocecal valve, right hemicolon, hepatic flexure, transverse colon, and splenic flexure);
- A subgroup population composed of subjects excluding those affected by the COVID-19 pandemic (treatment interruptions/delays, tumor assessment interruptions/delays).

(2) The definitions of stratified analyses are as follows:

- Stratified analysis: If applicable, stratification will be conducted according to ADA and Nab, and the efficacy of ADA-positive and -negative subjects, and Nab-positive and -negative subjects will be statistically analyzed. ADA-positive subjects are defined as those with at least one positive ADA test result after administration, and Nab-positive subjects are those with at least one positive Nab test result after administration.

**One-year Overall Survival Rate, Two-year Overall Survival Rate, and Overall Survival Time:**

One-year overall survival rate definition: The proportion of subjects alive at one year from randomization.

Two-year overall survival rate definition: The proportion of subjects alive at two years from randomization.

Overall survival time: Defined as the time from randomization to death (in months). For subjects still alive or lost to follow-up as of the data analysis cutoff date, survival is censored at the subject's last known alive time.

**Disease Control Rate (DCR):** Percentage of subjects responding with CR, PR, and SD (DCR=CR+PR+SD) using data assessed by IRaC.

**Clinical Benefit Rate (CBR):** Proportion of subjects whose tumors were assessed to be in CR, PR, and SD for 24 weeks or more according to RECIST 1.1, using data assessed by IRaC.

**Duration of Response (DOR):** Data assessed using IRaC, in accordance with RECIST 1.1, for subjects with a response of CR or PR, from the date of first presentation (CR or PR) to the date of first disease progression or death. Subjects were censored if they did not have an endpoint event, and the date of censoring was the date of the last imaging evaluation.

**Time to Response (TTR):** Data assessed using IRaC were performed according to RECIST 1.1 for subjects with a response of CR or PR. TTR was defined as the time from the date of randomization up to the date of the first appearance of response (CR or PR) according to RECIST 1.1.

**Quality of Life Assessment Indicators (QOL):** Quality of life assessment (QOL) is conducted using the EORTC QLQ-C30 questionnaire, with individual item categorical scores linearly transformed to a 0-100 scale. The questionnaire is self-reported, consisting of 30 items, and subjects are asked to complete it carefully. Assessments are conducted at baseline and subsequent visits every 8 weeks until withdrawal from the study and entry into the follow-up period.

**Resection Rate of Hepatic Metastasis:** The resection rate of hepatic metastasis = number of subjects undergoing radical surgery for liver metastases divided by the number of subjects; Radical hepatic metastases resection rate = number of subjects with complete resection (R0 resection) divided by the number of subjects.

Objective Response Rate (ORR), 1-year Overall Survival Rate, 2-year Overall Survival Rate, Disease Control Rate (DCR), Clinical Benefit Rate (CBR): The rates for both groups are calculated respectively, and the between-group comparison is made using the CMH (Cochran-Mantel-Haenszel) test method, and the 95% confidence interval of the rate difference between the two groups is calculated. At the same time, a Logistic regression model is used for analysis, with covariates (center, ECOG score) adjusted in the analysis to calculate the odds ratio (OR value) and its 95% confidence interval for each group.

Overall Survival (OS), 1-year Cumulative Survival Rate, 2-year Cumulative Survival Rate, Duration of Response (DOR): Between-group comparisons are made using the Log-rank test, and Kaplan-Meier survival curves are plotted. The risk ratio and its 95% confidence interval between treatment groups are calculated using the Cox proportional hazards model and covariates (center, ECOG score) specified between groups.

For TTR, only subjects who achieve response are included, and there is no censored data. It is analyzed as a quantitative indicator, and the comparison between the two groups is made using t-tests or other quantitative data comparison methods; at the same time, analysis of covariance is used, with TTR as the dependent variable, and covariates (center, ECOG score) are adjusted in the analysis to calculate the adjusted means (Lsmean) of the two groups and the difference in the adjusted means of TTR between the experimental group and the control group and its 95% confidence interval.

Statistical description of the quality of life scale scores is conducted, and the comparison between the two groups is made using t-tests or Wilcoxon rank-sum tests or other quantitative data comparison methods. Comparisons with the baseline values at the screening period are made using paired t-tests or Wilcoxon signed-rank tests, depending on the data distribution.

Proportion of subjects who underwent radical surgery for liver metastases and the rate of radical resection of liver metastases: rates were calculated separately for the two groups, while comparisons between groups were made using the chi-square test or Fisher's exact probability method.

## Safety Analysis

The Safety Set (SS) dataset is used, with a focus on descriptive statistical analysis.

### Drug Exposure

Group descriptions of the subjects' cumulative drug exposure dose, number of administration cycles, number of administrations, and mean, standard deviation, median, maximum, minimum, lower quartile, and upper quartile of dose intensity and relative dose intensity are provided. The specific treatment drugs include the anti-EGFR monoclonal antibody therapy drug (CMAB009) and FOLFIRI chemotherapy drugs (including irinotecan, leucovorin, and 5-fluorouracil).

The actual cumulative drug exposure dose refers to the sum of all actual total doses from the first administration date to the last administration date at the end of treatment. Dose intensity is calculated as follows:

Dose intensity = Actual cumulative total dose / Exposure duration

Where exposure duration refers to the period from the first administration date of the treatment drug to the last administration date at the end of treatment. Exposure duration (days) is calculated as follows:

Exposure duration (days) = Last administration date - First administration date + 1

Relative dose intensity refers to the ratio of the actual administered dose intensity to the standard dose intensity, calculated as follows:

Relative dose intensity = (Actual cumulative drug exposure total dose / Planned cumulative drug exposure total dose) × 100%

In addition, a summary is made of the impact of the COVID-19 pandemic on the subjects' medication use, with a group summary of the number and proportion of subjects whose medication was delayed or discontinued due to the COVID-19 pandemic, and a list is provided.

### Adverse Event

All adverse event (AE) analyses focus on treatment-emergent adverse events (TEAEs). TEAEs are defined as any AEs occurring from the first dose of the study drug to the end of the study visit. Treatment-related adverse events (TRAEs) are defined as any AEs occurring from the first dose of the study drug to the end of the study visit that are related to the study drug. Serious adverse events (SAEs) not related to the drug are collected from the first dose to the end of the study visit; SAEs related to the drug are collected at any time before the database lock. For centers that grade AEs, the same AEs are combined and included in the analysis.

The number of cases, number of occurrences, and incidence rate of adverse events in the following categories are summarized, and the chi-square test or Fisher's exact probability is used to compare the incidence rates between the two groups. The specific categories include:

- All adverse events;
- All TEAEs, TRAEs, and TEAEs related to CMAB009, where TRAEs are defined as being related to any study drug (including CMAB009, irinotecan, 5-fluorouracil, leucovorin);
- All SAEs and SAEs related to CMAB009 and FOLFIRI chemotherapy, respectively;
- TEAEs/TRAEs that lead to dose reduction of CMAB009 or any chemotherapy drug, respectively;
- TEAEs related to CMAB009 that lead to dose reduction of CMAB009 or any chemotherapy drug, respectively;
- TEAEs/TRAEs that lead to suspension of CMAB009 or any chemotherapy drug, respectively;
- TEAEs related to CMAB009 that lead to suspension of CMAB009 or any chemotherapy drug, respectively;
- TEAEs/TRAEs that lead to permanent discontinuation of CMAB009 or any chemotherapy drug, respectively;
- TEAEs related to CMAB009 that lead to permanent discontinuation of CMAB009 or any chemotherapy drug, respectively;
- TEAEs/TRAEs/TEAEs related to CMAB009 that require intervention;
- TEAEs/TRAEs/TEAEs related to CMAB009 that lead to withdrawal from the trial;
- TEAEs/TRAEs/TEAEs related to CMAB009 that lead to death;
- Infusion-related reactions/ Infusion-related reactions related to CMAB009/ Infusion-related reactions related to any chemotherapy drug: Infusion-related reactions are defined as "allergic reactions, hypersensitivity reactions, infusion reactions" occurring at any time during the clinical study, or "sinus tachycardia, sweating, fever, hypertension, chills, dyspnea, palpitations, myocardial ischemia, low blood pressure, increased blood pressure" occurring on the first day of administration.

All AEs are coded according to the SOC and PT using MedDRA version 25.0 Chinese. The following specific information is summarized by SOC and PT and group:

- The number of occurrences, number of cases, and incidence rate of TEAEs/TRAEs/TEAEs related to CMAB009;
- The number of occurrences, number of cases, and incidence rate of SAEs;
- The number of occurrences, number of cases, and incidence rate of SAEs related to CMAB009;
- The number of occurrences, number of cases, and incidence rate of SAEs related to chemotherapy drugs;
- Summarize the number of occurrences, number of cases, and incidence rate of TEAEs by grade 1, 2, 3, 4, and 5;
- Summarize the number of occurrences, number of cases, and incidence rate of TEAEs grade 3 and above by grade 3, 4, and 5;
- Summarize the number of occurrences, number of cases, and incidence rate of TRAEs by grade 1, 2, 3, 4, and 5;
- Summarize the number of occurrences, number of cases, and incidence rate of TRAEs grade 3 and above by grade 3, 4, and 5;
- Summarize the number of occurrences, number of cases, and incidence rate of TEAEs related to CMAB009 by grade 1, 2, 3, 4, and 5;
- Summarize the number of occurrences, number of cases, and incidence rate of TEAEs related to CMAB009 grade 3 and above by grade 3, 4, and 5;
- Summarize the number of occurrences, number of cases, and incidence rate of SAEs by grade 1, 2, 3, 4, and 5;
- Summarize the number of occurrences, number of cases, and incidence rate of SAEs related to CMAB009 by grade 1, 2, 3, 4, and 5;
- Summarize the number of occurrences, number of cases, and incidence rate of SAEs related to chemotherapy drugs by grade 1, 2, 3, 4, and 5;
- The number of occurrences, number of cases, and incidence rate of TEAEs/TRAEs that lead to dose reduction of CMAB009;
- The number of occurrences, number of cases, and incidence rate of TEAEs/TRAEs that lead to dose reduction of any chemotherapy drug;
- The number of occurrences, number of cases, and incidence rate of TEAEs/TRAEs that lead to suspension of CMAB009;
- The number of occurrences, number of cases, and incidence rate of TEAEs/TRAEs that lead to suspension of any chemotherapy drug;
- The number of occurrences, number of cases, and incidence rate of TEAEs/TRAEs that lead to permanent discontinuation of CMAB009;
- The number of occurrences, number of cases, and incidence rate of TEAEs/TRAEs that lead to permanent discontinuation of any chemotherapy drug;
- The number of cases and incidence rate of TEAEs/TRAEs that lead to withdrawal from the trial;
- The number of occurrences, number of cases, and incidence rate of TEAEs/TRAEs that require intervention;
- The number of cases and incidence rate of TEAEs/TRAEs that lead to death;
- The number of occurrences, number of cases, and incidence rate of infusion-related reactions;
- Summarize the number of occurrences, number of cases, and incidence rate of infusion-related reactions by grade 1, 2, 3, 4, and 5;
- Summarize the number of occurrences, number of cases, and incidence rate of infusion-related reactions grade 3 and above by grade 3, 4, and 5;
- TEAEs and TRAEs with an incidence rate of ≥10%, 5% in either the experimental group or the control group;
- TEAEs and TRAEs with an incidence rate difference of ≥10%, 5% between the experimental group and the control group;
- The number of TEAEs/TRAEs and their incidence rate in ADA-positive and -negative subjects in the experimental group;
- The number of TEAEs/TRAEs and their incidence rate in Nab-positive and -negative subjects in the experimental group (if applicable).

A list of AEs and SAEs is provided, with detailed information including subject number, group, AE name, SOC, PT, whether it is a TEAE, start date, end date, severity, whether it is an SAE, relationship to the study drug, measures taken, outcome, and whether it leads to withdrawal from the trial, etc.

### Vital Signs, Body Weight and ECOG Scores

Descriptive statistics will be performed on the measurements of vital signs (temperature, heart rate/pulse, respiration, and blood pressure), body weight, and ECOG scores at each follow-up visit for each group, as well as the changes from baseline. Baseline is defined as the last measurement value before receiving study drug treatment.

### Physical Examination

A cross-tabulation table (recording the number of cases) will be used to describe the changes before and after administration. Baseline is defined as the last examination result before receiving study drug treatment, and post-treatment is defined as the worst examination result during the treatment period after receiving study drug treatment. A list will be used to describe subjects with clinically meaningful changes in examination results.

### Laboratory Tests

A cross-tabulation table will be used to describe the changes before and after administration. Baseline is defined as the last test value before receiving study drug treatment, and post-treatment is defined as the worst test result during the treatment period after receiving study drug treatment. A list will be used to describe subjects with clinically meaningful changes in test results.

### Electrocardiogram (ECG)

A cross-tabulation table will be used to describe the changes before and after administration. Baseline is defined as the last test value before receiving study drug treatment, and post-treatment is defined as the worst test result during the treatment period after receiving study drug treatment. A list will be used to describe subjects with abnormal ECG findings during the trial.

## Other Indicator Analysis

### Pharmacokinetic Evaluation

Pharmacokinetic analysis will be conducted using the PK parameter set.

Blood concentration (c)-time (t) data analysis: Individual and average c-t curves, semi-log c-t curves will be plotted using PKCS; average blood concentration-time curves (linear and semi-log) will be plotted according to the scheduled blood collection time points; individual blood concentration-time curves (linear and semi-log) will be plotted according to the actual blood collection time points. In addition, by plotting the blood concentration-time data before and after multiple administrations, the steady-state of the drug will be assessed.

When summarizing PK concentrations, provide the evaluable n, Mean, SD, %CV, Median, Min, and Max. For quantitative lower limits (BLQ) concentration data, it will be set to "0" in the statistical summary of blood concentration data.

PK parameter calculations will be completed using Phoenix WinNonlin 8.1 or higher, using PKPS, non-compartmental model calculations of each subject's pharmacokinetic parameters, calculating the arithmetic mean, standard deviation, coefficient of variation, median, maximum value, minimum value, geometric mean, and geometric mean coefficient of variation for each parameter. If a subject's AUC__%Extrap_＞20%, AUC_0-∞_, t_1/2_, λ_z_, and AUC__%Extrap_ will not be included in the descriptive statistical analysis. All PK parameter calculations will be based on actual PK sampling times. If data permits, the impact of immunogenicity status on drug PK parameters will be assessed, and PK parameters (AUC_tau,ss_, C_max,ss_, C_trough,ss_, CL_ss_, V_z,ss_) will be descriptively statistically analyzed according to the subject's detected ADA/Nab status (positive or negative).

For PK parameter calculations, data below the lower limit of quantification (LLOQ) are marked as BLQ. BLQ data before the first measurable concentration point and/or after the last measurable concentration point will be set to 0 in PK concentration summary and plotting (linear). Single or multiple consecutive BLQ data between measurable concentration values are defined as missing. Individual PK parameters will display the same number of decimal places or significant digits as the original concentration data, but percentages will be retained to one decimal place, and Tmax will be retained to two decimal places. If the precision of the parameter's original data is low, the precision of the original data will be used.

### Immunogenicity Evaluation

Statistical analysis will be conducted using the immunogenicity dataset.

Blood samples will be used to determine the changes in anti-CMAB009 antibody production after treatment in subjects. For subjects who are ADA-positive, the changes in neutralizing antibodies (Nab) will also be further determined.

Immunogenicity evaluation will use descriptive statistical analysis, respectively, statistical analysis of the ADA and Nab (if applicable) positive rates at baseline and each time point in the experimental group. At the same time, a list of the subject's immunogenicity data will be provided.

# Statistical Tables and Figures

The directory of statistical charts is in "Attachment 1: Directory of Statistical Charts".

# Attachments

**Attachment: Statistical Analysis Method Code**

**Survival Curve Models and Codes**

| **SAS program code for survival curve model** |
| --- |
| **proc** **lifetest** data=dataset;  time time*censor(0);  strata treat;  **quit**;  The above code parameters are defined as follows.  time:progress/survival time.  censor: progress/survival state and truncated state value, where the above (0) is defined as a truncated value of 0.  treat:Research Subgroup.  The above code specifies the calculation and output of the number of events, the cut-off number and the cumulative event rate of each group, and the calculation of the median progression-free/survival time and 95% confidence intervals of each group, the Logrank rank statistic and the P-value for the comparison between groups, and the output of the Kaplan-Meier survival curves of the two groups (with the number of people at risk at the beginning of the period and the censored information). |

**Cox scale models and codes**

| **SAS program code for Cox proportional risk models** |
| --- |
| **proc phreg** data = dataset;  class treat center ecog;  model time*censor(0) = treat center ecog/risklimits;  **quit**;   - time: time of progress. - censor: progress state and truncated state value, where the above (0) is defined as a truncated value of 0. - treat: research subgroup. - center:center. - ecog:ECOG layering.   The above code specifies the output model coefficients, error, chi-square statistic for between-group comparisons, p-value, risk ratio HR, and 95% confidence interval for the HR. |

**Logistic regression model and code**

| **SAS Program Code for Logistic Regression Models** |
| --- |
| **proc** **logistic** data=dataset;  class treat(param=reference ref="control").  class center(param=reference ref=first);  class ecog(param=reference ref=first);  model yvar=treat center ecog/aggregate scale=pearson rsquare risklimits;  **quit**;   - yvar: dependent variable, corresponding to the endpoint event of interest to the study, e.g., ORR - treat: research subgroup. - center: covariate, center. - ecog: covariates, ECOG stratification.   The above codes estimate and output the ratio ratio (OR value) and 95% confidence interval for the occurrence of the endpoint event in the test group relative to the control group after correction for center and ECOG scores. |

**Covariance model and code**

| **SAS program code for the covariance model** |
| --- |
| **proc glm** data = dataset;  class treat center ecog;  model ttr = treat center ecog/solution ss3;  lsmeans treat/ stderr pdiff tdiff cl;  **quit**;   - ttr: dependent variable, time to mitigation. - treat: research subgroup. - center: covariate, center. - ecog: covariates, ECOG stratification.   The above code specifies the statistics, p-values, and corrected mean comparisons for between-group comparisons of the output covariates (corrected mean and error for the test and control groups, difference in corrected means and 95% confidence intervals for the test and control groups). |
